# Supplementary material for: Examining the quality of care across the continuum of maternal care (antenatal, perinatal and postnatal care) under the expanded free maternity policy (Linda Mama Policy) in Kenya: a mixed-methods study
Source: BMJ Open. 2024 May 2;14(5):e082011. doi: 10.1136/bmjopen-2023-082011 (PMC11086406; doi:10.1136/bmjopen-2023-082011)
Supplement: Supplementary data [file bmjopen-2023-082011supp001.pdf]

Appendix 1: Maternal healthcare access characteristics

**Variable**		**Total Frequency (%) n=550**	**Hospital A n (%) n=42**	**Hospital B n (%) n=170**	**Hospital C n (%) n=338**	**p-value**
Facility visited during pregnancy	Yes	545 (99.09)	42 (100)	169 (99.41)	334 (98.82)	0.650
	No	5 (0.91)	0	1 (0.59)	4 (1.18)	
Type of facility visited	Public facility	506 (92.00)	38 (90.48)	149 (87.65)	319 (94.38)	**P<0.001***
	Private facility	28 (5.09)	1 (2.38)	17 (10.00)	10 (2.96)	
	Faith based organization (Mission)	7 (1.27)	-	3 (1.76)	4 (1.18)	
	Other	9 (1.64)	3 (7.14)	1 (0.59)	5 (1.48)	
Time taken to reach hospital	Below 30 minutes	137 (24.91)	12 (28.57)	45 (26.47)	80 (23.67)	0.309
	30 minutes-1 hour	264 (48.00)	19 (45.24)	87 (51.18)	158 (46.75)	
	1 hour-2 hours	121 (22.00)	7 (16.67)	35 (20.59)	79 (23.37)	
	More than 2 hours	20 (3.64)	4 (9.52)	2 (1.18)	14 (4.14)	
	Don’t know	8 (1.45)	-	1(0.59)	7 (1.96)	
Perception of the time take to reach the hospital	Very short	60 (10.91)	6 (14.29)	25 (14.71)	29 (8.58)	0.340
	Short	249 (45.27)	18 (42.86)	73 (42.94)	158 (46.75)	
	Normal	99 (18.00)	12 (28.57)	32 (18.82)	55 (16.27)	
	Long	107 (19.45)	5 (11.90)	29 (17.06)	73 (21.60)	
	Very long	32 (5.82)	1 (2.38)	11 (6.47)	20 (5.92)	
	Don’t know	3 (0.54)	-	-	3 (0.89)	
	Very near	69 (12.55)	16 (38.10)	22 (12.94)	31 (9.17)	**P<0.001**

**Variable**		**Total Frequency (%) n=550**	**Hospital A n (%) n=42**	**Hospital B n (%) n=170**	**Hospital C n (%) n=338**	**p-value**
Perception about distance to the facility	Normal	339 (61.64)	17 (40.48)	107 (62.94)	215 (63.61)	
	Far	110 (20.00)	8 (19.05)	33 (19.41)	69 (20.41)	
	Very far	28 (5.09)	-	8 (4.71)	20 (5.92)	
	Don't know	4 (0.73)	1 (2.38)	-	3 (0.89)	
Means of transport to the facility	Walking	27 (4.91)	7 (16.67)	14 (8.24)	6 (1.78)	**P<0.001**
	Bi/Motorcycle	60 (10.91)	1 (2.38)	20 (11.76)	39 (11.54)	
	Public transport (matatu/tuk tuk)	224 (40.73)	8 (19.05)	55 (32.35)	161 (47.63)	
	Private car/taxi	211 (38.36)	24 (57.14)	78 (45.88)	109 (32.25)	
	Ambulance	22 (4.00)	-	1 (0.59)	21 (6.21)	
	Combined modes	6 (1.09)	2 (4.76)	2 (1.18)	2 (0.59)	
Does opening hour suit your time?	Yes	431 (78.36)	41 (97.62)	152 (89.41)	238 (70.41)	**P<0.001**
	No	9 (1.64)	1 (2.38)	1 (0.59)	7 (2.07)	
	Don't know	76 (13.82)	-	15 (8.82)	61 (18.05)	
	N/A	34 (6.18)	-	2 (1.18)	32 (9.47)	
Waiting time at the facility	Very short	80 (14.55)	12 (28.57)	26 (15.29)	42 (12.43)	**P<0.001**
	Short	237 (43.09)	16 (38.10)	72 (42.35)	149 (44.08)	
	Normal	70 (12.73)	11 (26.19)	28 (16.47)	31 (9.17)	
	Long	80 (14.55)	1 (2.38)	22 (12.94)	57 (16.86)	
	Very long	43 (7.82)	2 (4.76)	22 (12.94)	19 (5.62)	
	N/A	40 (7.27)	-	-	40 (11.83)	

**Variable**		**Total Frequency (%) n=550**	**Hospital A n (%) n=42**	**Hospital B n (%) n=170**	**Hospital C n (%) n=338**	**p-value**
Hospital have a proper waiting area	Yes	422 (76.73)	40 (95.24)	134 (78.82)	248 (73.37)	**0.005**
	No	85 (15.45)	1 (2.38)	29 (17.06)	55 (16.27)	
	Don't know	28 (5.09)	1 (2.38)	7 (4.12)	20 (5.92)	
	N/A	15(2.73)	-	-	15 (4.44)	

Note: Chi square test of proportion was used to test difference in overall proportions of maternal health access characteristics.
*There is a statistical difference in the type of facilities that the mothers visited (majority visited public facilities).
Bold means **p-value <0.05**

Appendix 2: Referral characteristics

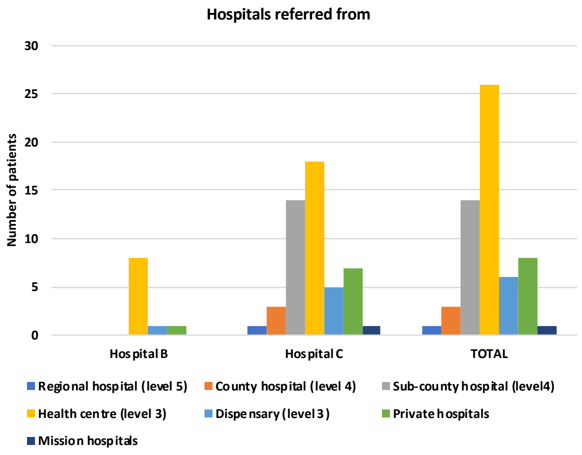

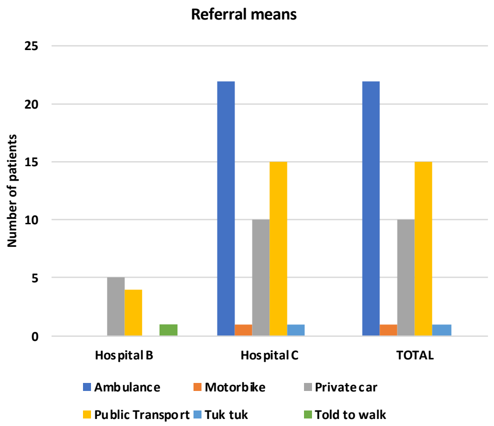

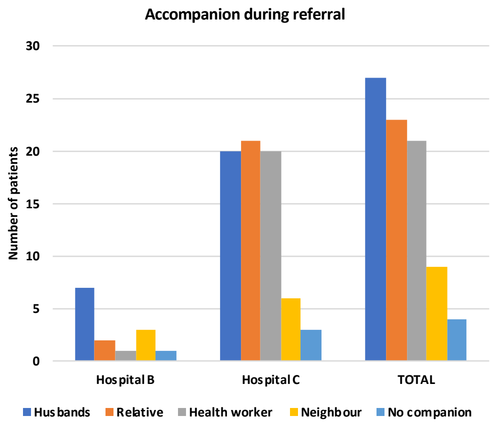

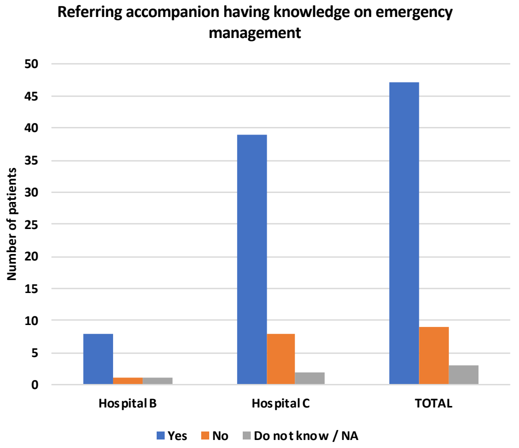

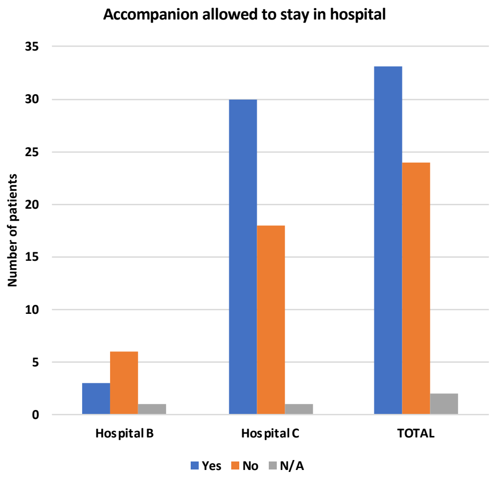

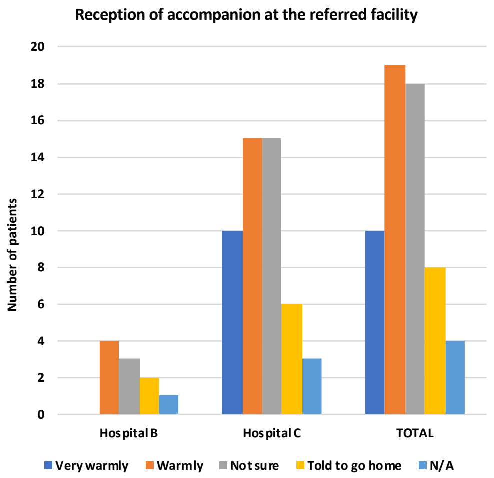

Appendix 3: Reasons for referral

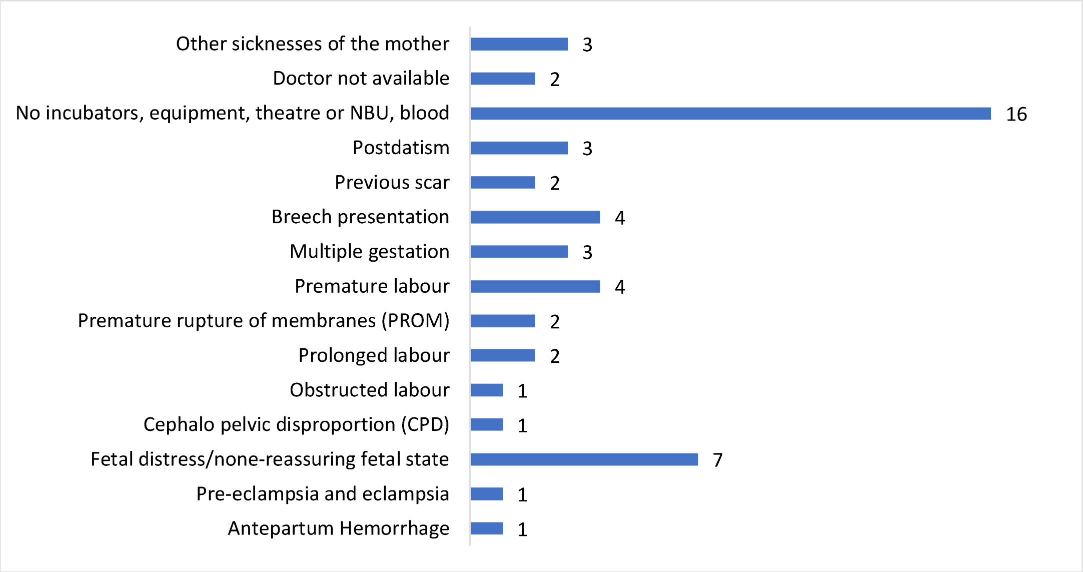

Appendix 4: Perception of quality of maternal care from the mothers exit interviews

**Health Facility**	**Completely Disagree**	**Disagree**	**Not Sure**	**Agree**	**Completely Agree**
Number of staff adequate	0.73%	19.09%	3.82%	57.82%	18.55%
Staff well suited to treat women	0.36%	1.82%	1.27%	70.73%	25.82%
Waiting and examination rooms adequate	5.82%	38.73%	3.82%	40.55%	11.05%
Provision of clean drinking water adequate	3.82%	38.36%	11.46%	34.00%	12.18%
Hand washing facilities adequate	1.45%	6.00%	0.73%	70.55%	21.27%
Bathing facilities adequate	3.82%	24.36%	4.36%	52.91%	14.55%
Toilet facilities adequate	2.91%	24.00%	1.64%	55.45%	16.00%
Overal facility environment very clean	0.91%	3.64%	2.00%	70.00%	23.45%
Well suited equipment for detecting women's problems	0.91%	3.45%	4.73%	69.82%	21.09%
Distance from home very far	8.55%	59.09%	2.18%	23.82%	6.36%
**Healthcare delivery**	**Completely Disagree**	**Disagree**	**Not Sure**	**Agree**	**Completely Agree**
Staff examine pregnant and post partum women well	0.91%	2.00%	1.82%	72.00%	23.27%
Staff very capable of finding out what is wrong with patients	0.73%	1.64%	2.18%	71.09%	24.36%
Staff prescribe drugs that are needed	0.00%	2.91%	37.45%	42.91%	16.73%
Drugs supplied by health facility are good	0.36%	1.45%	39.82%	42.55%	15.82%
Patients can obtain drugs from health facility easily	1.45%	5.64%	25.64%	52.00%	15.27%
Facility provided privacy very much during VE and delivery	3.82%	9.64%	6.73%	63.82%	16.00%
Felt very much of necessary procedure during ANC and delivery	3.83%	8.38%	3.10%	65.39%	19.31%
Adequate Information on danger signs of delivery and postpartum	1.45%	0.24%	3.27%	49.45%	21.82%
**Interpersonal Aspects**	**Completely Disagree**	**Disagree**	**Not Sure**	**Agree**	**Completely Agree**
Staff very open with the patients	0.18%	3.83%	1.64%	68.61%	25.73%
Staff very compasionate towards the patients	1.27%	5.45%	2.73%	66.00%	24.58%

Staff are respectful towards the patients	0.18%	2.73%	1.64%	69.64%	25.82%
Time staff devete to the patients is adequate	0.36%	4.36%	1.09%	67.27%	26.91%
Staff are very honest	0.00%	2.36%	5.64%	65.64%	26.36%
