# Supplementary material for: Examining the quality of care across the continuum of maternal care (antenatal, perinatal and postnatal care) under the expanded free maternity policy (Linda Mama Policy) in Kenya: a mixed-methods study
Source: BMJ Open. 2024 May 2;14(5):e082011. doi: 10.1136/bmjopen-2023-082011 (PMC11086406; doi:10.1136/bmjopen-2023-082011)
Supplement: Author's manuscript [file bmjopen-2023-082011.draft_revisions.pdf]

BMJ Open is committed to open peer review. As part of this commitment we make the peer review history of every article we publish publicly available.

When an article is published we post the peer reviewers' comments and the authors' responses online. We also post the versions of the paper that were used during peer review. These are the versions that the peer review comments apply to.

The versions of the paper that follow are the versions that were submitted during the peer review process. They are not the versions of record or the final published versions. They should not be cited or distributed as the published version of this manuscript.

BMJ Open is an open access journal and the full, final, typeset and author-corrected version of record of the manuscript is available on our site with no access controls, subscription charges or pay-per-view fees (http://bmjopen.bmj.com).

If you have any questions on BMJ Open's open peer review process please email info.bmjopen@bmj.com

BMJ Open

Examining the quality of care across the continuum of maternal care (antenatal, perinatal, and postnatal care) under the free maternity policy in Kenya.

Journal:	*BMJ Open*
Manuscript ID	bmjopen-2023-082011
Article Type:	Original research
Date Submitted by the Author:	16-Nov-2023

Complete List of Authors:	Oyugi, Boniface; M and E Advisory Group, The Mint, Western Heights, Karuna Road P.O. Box 6523 – 00200 Nairobi; University of Kent, Centre for Health Services Studies<br>Audi-Poquillon, Zilper; London School of Economics and Political Science, Department of Health Policy<br>Kendall, Sally; University of Kent, Centre for Health Services Studies<br>Peckham, Stephen; University of Kent, Centre for Health Services Studies

Keywords:	Health economics < HEALTH SERVICES ADMINISTRATION & MANAGEMENT, Health policy < HEALTH SERVICES ADMINISTRATION & MANAGEMENT, Maternal medicine < OBSTETRICS, Quality in health care < HEALTH SERVICES ADMINISTRATION & MANAGEMENT, Community child health < PAEDIATRICS

SCHOLARONE™
Manuscripts

I, the Submitting Author has the right to grant and does grant on behalf of all authors of the Work (as defined in the below author licence), an exclusive licence and/or a non-exclusive licence for contributions from authors who are: i) UK Crown employees; ii) where BMJ has agreed a CC-BY licence shall apply, and/or iii) in accordance with the terms applicable for US Federal Government officers or employees acting as part of their official duties; on a worldwide, perpetual, irrevocable, royalty-free basis to BMJ Publishing Group Ltd ("BMJ") its licensees and where the relevant Journal is co-owned by BMJ to the co-owners of the Journal, to publish the Work in this journal and any other BMJ products and to exploit all rights, as set out in our licence.

The Submitting Author accepts and understands that any supply made under these terms is made by BMJ to the Submitting Author unless you are acting as an employee on behalf of your employer or a postgraduate student of an affiliated institution which is paying any applicable article publishing charge ("APC") for Open Access articles. Where the Submitting Author wishes to make the Work available on an Open Access basis (and intends to pay the relevant APC), the terms of reuse of such Open Access shall be governed by a Creative Commons licence – details of these licences and which Creative Commons licence will apply to this Work are set out in our licence referred to above.

Other than as permitted in any relevant BMJ Author's Self Archiving Policies, I confirm this Work has not been accepted for publication elsewhere, is not being considered for publication elsewhere and does not duplicate material already published. I confirm all authors consent to publication of this Work and authorise the granting of this licence.

For peer review only

Examining the quality of care across the continuum of maternal care (antenatal, perinatal, and postnatal care) under the free maternity policy in Kenya.

Authors

Boniface Oyugi[1,2], **Zilper Audi-Poquillon**[3*], **Sally Kendall**[2], **Stephen Peckham**[2]

1. M and E Advisory Group, P.O. Box 6523 - 00200, Western Heights, The Mint Nairobi, Kenya.
2. Centre for Health Services Studies (CHSS), University of Kent, George Allen Wing, Canterbury CT2 7NF
3. Department of Health Policy, London School of Economics and Political Science

***Corresponding Author: Zilper Audi-Poquillo (ZAP)** – z.a.audi-poquillon@lse.ac.uk

Email addresses:
BO: b.oyugi@kent.ac.uk
SK: S.Kendall-608@kent.ac.uk
SP: S.Peckham@kent.ac.uk

ORCID IDs
Boniface Oyugi: https://orcid.org/0000-0002-9550-9138
Zilper Audi-Poquillon: https://orcid.org/0009-0002-6901-7348
Sally Kendall: https://orcid.org/0000-0002-2507-0350
Stephen Peckham: https://orcid.org/0000-0002-7002-2614

Qualification
BO: PhD (Dr)
ZAP: MSc (Ms)
SK: PhD (Professor)
SP: (Professor)

ABSTRACT

Background
While Kenya's maternal and child health status has significantly improved in the last decade, mothers and neonates are still dying from preventable pregnancy-related complications. Kenya addresses this challenge through a free maternity policy (FMP) implemented in 2013 and modified in 2017. This study examines the quality of care (QoC) across the continuum of maternal care under the FMP in Kenya.

Methods
We conducted a convergent parallel mixed-methods study, which involved key informant interviews (KIIs) with national stakeholders (n=15); in-depth interviews (IDIs) with County officials and health care workers (HCWS) (n=21); exit interview survey with mothers (n = 553) who utilised FMP delivery services, and nine focus group discussions (FGDs) with mothers who returned for postnatal visits (6,

For peer review only - http://bmjopen.bmj.com/site/about/guidelines.xhtml

10, and 14 weeks). Data were analysed using a framework approach guided by the QoC for Maternal and Newborn – a monitoring framework for network countries.

Results

The results showed that the FMP enhanced maternal care access: geographical, financial, and service utilisation. However, the facilities and HCWs bore the brunt of the increased workload and burnout. There was a longer waiting time for the initial visit by the pregnant women because of the enhanced ANC package of the FMP. The availability and standards of equipment, supplies, and infrastructure still pose challenges. Nurses were multitasking and were motivated despite the human resources challenge. Mothers were happy to have received care information; however, there were challenges regarding respect and dignity they received (inadequate food, over-crowding, bedsharing and lack of privacy), and they experienced physical, verbal, and emotional abuse and a lack of attention/care.

Conclusions

There is a need to address the negative aspects of QoC while strengthening the positives. This would help to achieve the SDG and UHC goals to ensure reduced maternal morbidities and mortalities through access to quality service for every woman.

Keywords: quality of care, maternal and childcare, free maternity policy **KEY QUESTIONS**

What is already known?

- Maternal deaths still make up approximately 15% of all deaths among women of reproductive age (approximated at 7,300 women dying yearly), with mothers and neonates still dying from preventable pregnancy-related complications.
- The Kenyan government instituted a free maternity policy to reduce catastrophic expenditure on maternity care and enhance the quality of healthcare service delivery.

What are the new findings?

- This paper examined the quality of care across the continuum of maternal care (antenatal, perinatal, and postnatal care) under the LM Policy in Kenya.
- The FMP policy provided positive results in the quality of maternal care across all the broad quality domains: access to care (equitable and timely), provision of care (safe and effective), management and organisation, and the experience of care.
- The policy helped to minimise access barriers (cultural, financial, geographic), enhanced some elements of timeliness of care, increased provider availability and created functional referral systems and safety, and improved the availability of essential physical resources and competent and motivated staff (though not in all hospitals).
- Women in the study had a good care experience, which included reception of prompt maternal services, good care for the baby after birth, teaching about birth procedures, breastfeeding, and family planning.
- There were cross-cutting poor experiences that the women faced such as overcrowding of the healthcare facilities, inadequate food supply, lack of communication of treatment plans and experiencing both physical and verbal abuse.

For peer review only

What do the new findings imply?

- There is a need to address the negative aspects of the study while strengthening the positives to achieve the SDG and UHC goals that seek to ensure reduced maternal morbidities and mortalities through access to quality service for every woman.

INTRODUCTION

There are nearly 287,000 maternal deaths due to preventable pregnancy and childbirth-related complications happening globally (translating to almost 800 maternal deaths every day or one every two minutes). [1] Low-and-middle-income countries (LMIC) and low-income countries (LIC), especially those in sub-Saharan Africa (SSA), such as Kenya, are the most affected because of barriers to accessing maternal services (such as low quality of care (QoC), poor socio-economic conditions, poor infrastructure, and lack of well-trained healthcare professionals). [2-4] While Kenya's maternal and child health status has significantly improved in the last decade, the current maternal mortality ratio (MMR) of 530 deaths per 100,00 live births is much higher than the global average of 223 maternal deaths per 100,000 live births, [1] as is the neonatal mortality rate (NMR) of 21 deaths per 1,000 live births which is higher than the global average of 18 deaths per 1,000. [5 6] Maternal deaths still make up approximately 15% of all deaths among women of reproductive age (approximated at 7,300 women dying every year), with both mothers and neonates dying from preventable pregnancyrelated complications. [7] One in 76 women in Kenya is at risk of dying from pregnancy complications. [8]

As such, reducing and eliminating pregnancy-related mortality remains a priority to progress towards achieving Sustainable Development Goals (SDGs) goals. There have been various reforms in the health sector in Kenya that seek to reduce catastrophic expenditure on maternity care and improve the quality of healthcare service delivery. [9-13] In June 2013, the government initiated a user fee waiver for maternity and primary health care (PHC) services. [9] However, its implementation was faced with challenges of poor service delivery due to inadequate preparation before the implementation and a lack of adequate systems to verify the QoC provided and the reimbursement claims from the hospitals to the government. [14] Subsequently, to overcome these challenges, the country transitioned to a new expanded free maternity policy (FMP) in 2017 to provide access to maternal services to all pregnant women in private, faith-based, and all level 3–6 public institutions. [15] The expanded policy was called Linda Mama (LM) (Swahili for "*caring for the mother*"), and was managed through the National Hospital Insurance Fund(NHIF).

The strategic objective of Universal Health Coverage (UHC) captures three facets: population, services and direct costs. It envisages 'all people having access to the full range of quality health services they need, when and where they need them, without financial hardship.' [16 17] The LM policy was mainly implemented to achieve the three facets. However, following the implementation of the FMP policy, researchers have focussed on understanding two of the three facets: population and cost, through studies focused on the policy's immediate and trend effect, [18] its impacts on mortality and utilisation of services, [19-22] out of pocket expenditure, [23] and the cost-benefit analysis. [22]

Researchers have attempted to evaluate the resulting quality of care aspects from the FMP, but this has not been conclusive. For instance, one study evaluated the satisfaction with the delivery services under FMP. [24] It showed that the mothers who benefited from the services were satisfied with different components such as communication by the healthcare workers, staff availability in the delivery rooms, availability of staff in the wards, and drug and supplies availability but were also

For peer review only - http://bmjopen.bmj.com/site/about/guidelines.xhtml

unsatisfied with consultation time, cleanliness, and privacy in the wards. Another study evaluating the utilisation of the free maternity services implemented in 2013 among women living in Kibera slums in Nairobi showed that mothers positively perceived the distance to the facility and shorter waiting time, in addition tofa patients facing ill providers' attitudes. [25] Yet, another study that evaluated disrespectful maternal care under the policy in Kisii and Kilifi counties showed that mothers experienced disrespectful maternal care throughout the maternity process, and it appeared even more significant among women who were poor, young, or had children with disabilities. [26] All three studies on quality have focused on one aspect of quality: the outcome (from the patient perspective), leaving out other quality dimensions that researchers[27 28] have discussed: structure, process, and outcome.

Therefore, as part of achieving the UHC agenda, the quality facet is yet to be fully explored. Increasing service coverage alone is unlikely to produce better health outcomes without attention to the quality of care provided. The LM policy seeks to be a high-quality health intervention, defined as one 'that optimises health care in a given context by consistently delivering care that improves or maintains health outcomes, by being valued and trusted by all people, and by responding to changing population needs' is imperative. [29] Maternal care under LM policy envisages enhancing the degree to which maternal services received by clients increase the likelihood of desired health outcomes consistent with current professional knowledge and are effective, safe, people-centred, timely, equitable, integrated and efficient. [30] Therefore, exploring the optimal quality of maternal care and outcomes from the LM policy would be imperative. This study examines the quality of care across the continuum of maternal care (antenatal, perinatal, and postnatal care) under the LM Policy in Kenya.

METHODS Study Design

We utilised the convergent mixed methods design, specifically the parallel-database variant in this study[31] using qualitative and quantitative data that were collected and analysed in tandem and then compared and combined to better understand the quality of care across the continuum of maternal care (antenatal, perinatal, and postnatal care) under the free maternity policy in Kenya.

Framework for analysis

As quality cannot be measured by itself, [32] in this study, we conceptualised quality from the Donabedian perspective, broadly classifying quality as structure, process, and outcome dimensions[27 28], which can be identified, measured, and attributed to healthcare. Akachi and Kruk[33] provide more details on measuring changes in the QoC and bring attention to including user experience as a measure of outcomes in the quality assessment. With these two refined aspects, we broadly defined the structure indicators as pointers which are inputs to or characteristics of health; process indicators as gauges to either appropriate or inappropriate care in a targeted population which are 'consistent with current professional knowledge'; and outcome indicators as the measures of both improved or deteriorated health and attributed to medical care. [32 33] (See, Figure 1). Data collection methods and tools were designed to collect and examine all aspects of QoC across the continuum of maternal care (antenatal, perinatal, and postnatal care) under the free maternity policy in Kenya. Broadly, the analysis converges all the concepts using the QoC for Maternal and Newborn – a monitoring framework for network countries, [34] which draws concepts from the earlier framework as proposed by the World Health Organisation. [35]

Conceptualising quality of care *(Donabedian model)* & Measuring quality of care *(Akachi and Kruk model)*

Structure

Facility infrastructure Health workers

For peer review only

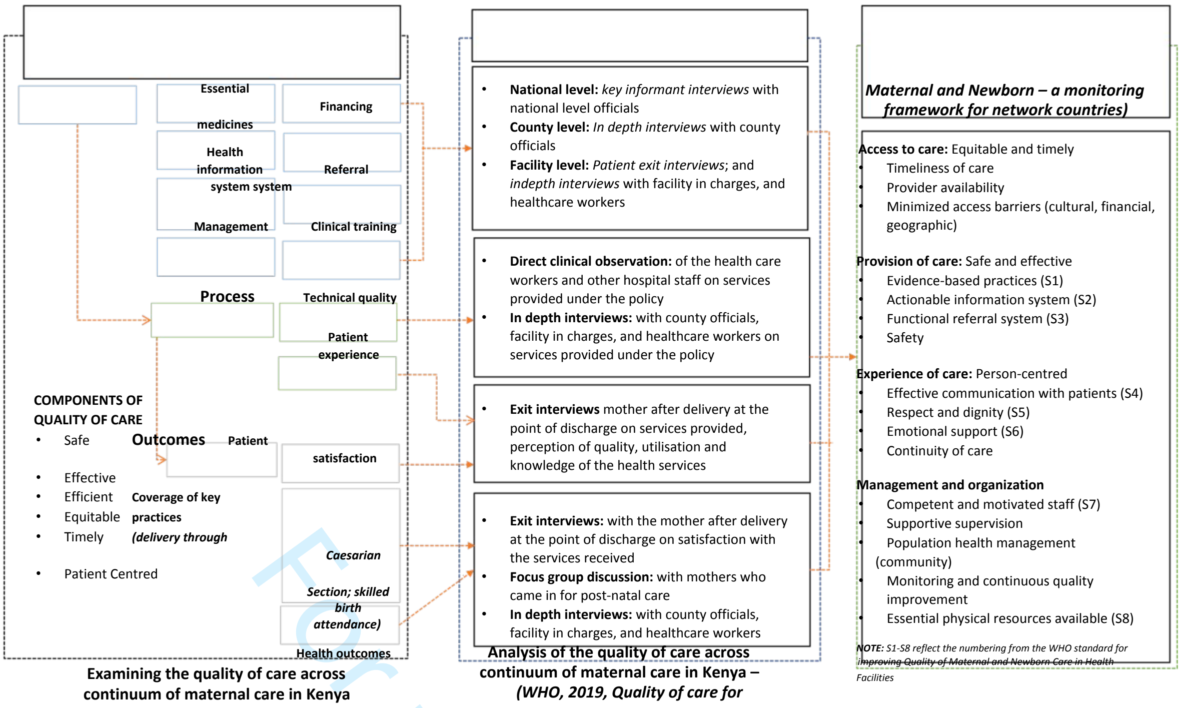

Figure 1: Combined frameworks used in this study for examining the quality of care across the continuum of maternal care (antenatal, perinatal, and postnatal care) under the free maternity policy in Kenya.

Study setting

The study was conducted across multiple levels within the Kenyan health system. The Kenyan health system is pluralistic in the provision and financing of services and is organised into six levels of care. Level 1 forms the community units overseen by community health workers (CHWs) whose role is providing promotive services (health education, treating minor ailments, and identifying cases that require referral to health facilities), [36] and both level 2 (dispensaries) and level 3 (health centres) provide primary healthcare services in addition to coordinating the community in their areas of jurisdiction. Level 4 and 5 offer curative services as county secondary referral facilities, with some being training centres, while level 6 are semi-autonomous tertiary facilities offering specialised care and serving as training institutions.

At the national level, we included the Ministry of Health, the NHIF, and development partner agencies involved in the expanded free maternity policy. At the County level, this study was conducted in Kiambu County in Kenya. The county was purposefully chosen because of the logistic feasibility of data collection (due to its proximity to Nairobi County and the cost implication accompanying data collection) and the sociodemographic characteristics, health indicators and population size. [37-39] It is the second-most populous county in Kenya after Nairobi City County, with a population of 2,417,735: 49.1% male and 50.59% female[37] 26.9% of the population in Kiambu are female of reproductive age (15-49 Years), [38] and 89.2% of births in the county happen in a health facility and 98.2% of births provided by a skilled provider. [5]

We purposefully selected three study facilities: a level 3 (considered a low volume – few numbers of clients), a level 4 (medium volume), and a level 5 (high volume). The facilities were chosen in consultation with the county team to provide nuanced, unique sub-counties dynamics given their richness in information and characteristics. (See Table 1).

For peer review only

For peer review only - http://bmjopen.bmj.com/site/about/guidelines.xhtml

Study population, sampling, and data collection

The study population used in this study were in four categories, as summarised in Table 1. We collected data between November 2018 and September 2019 through EIs, FGDs, IDIs, and KIIs.

The first group was staff from the Ministry of Health, NHIF, and development partners, who were purposefully selected based on their level of involvement in the expanded free maternity policy. These respondents participated in KIIs with one researcher (BO), which were done in English, using KII guides developed to capture the experience of the formulation and implementation of FMP. All the KIIs were conducted in Nairobi and were audiotaped following participants' consent using audio recorders. Each KII lasted between 45-60 minutes.

The second category included purposively selected respondents with knowledge of and experience in the implementation of the FMP at the county (meso) level (including county and sub-county level officials from the County Department of Health); and the facility (micro) level (including facility incharge, HCWs in charge of /offering maternal care/services, and other cadres of hospital workers) (Table 1). These respondents participated in IDI with one researcher (BO). The IDIs were conducted in English using two semi-structured guides (each for the county and health facility participants) developed to capture the experience of implementing FMP. The two semi-structured guides' construct validity was tested in the non-participating facility to check for ambiguity and flow of the questions. All the IDIs (save for one conducted at the place of convenience for the participant) were conducted at the participants' places of work and were audiotaped using audio recorders after obtaining their consent. Each IDI lasted between 30-60 minutes.

The third group comprised of EIs with mothers who had delivered in the three hospitals and were discharged home. The sample size of the mothers was estimated at 553 using the formula proposed by Gorstein et al. [40] A detailed discussion of the sample criteria and dynamics across the three selected facilities has been published elsewhere. [41] Four trained data collectors, supervised by one researcher (BO), conducted the EIs with the women. The design of the EI utilised a structured questionnaire, adapted from Dalinjong et al., [42] to elucidate the sociodemographic information of the women, health and related services received at the facility (perception of the quality of maternal care that the mothers received during delivery and ANC care, experiences with the FM policy). The conduct of the EIs ensured that one researcher (BO) introduced the data collectors to the administration and the maternity department heads of the three facilities; then, each morning of the interview, they identified the mothers who had been discharged (using bed numbers) and were waiting to return home. With the number of mothers identified per day, we generated a random sample using Stat Trek's Random number generator[43], which was used to identify mothers for the EI. The mothers were then invited to participate in the study, and interviews were conducted until we reached the intended sample size. We took each mother through the information sheet, and only when they were comfortable participating did, we give them the consent forms. One mother declined to participate (and eliminated two entries at the analysis stage for lacking complete information).

The final category included FGD with nine groups of mothers (ranging from 5-12 mothers) purposively selected based on a common interest: mothers who had had a skilled delivery in a hospital setting and had come to the study sites for the 6-, 10-, or 14- week postnatal visits. One researcher (BO) conducted all 9 FGDs in Swahili (given the different levels of knowledge of the participants) using an FGD guide

For peer review only - http://bmjopen.bmj.com/site/about/guidelines.xhtml

developed in reference to the gaps that had arisen from the EIs. The mothers in the FGD were recruited from the child welfare clinic of the three facilities when they brought their children for routine vaccination. The FGDs in each facility were organised with the help of a nurse from the maternity departments. We engaged the mothers as the children received their vaccinations and asked if they would participate in the study. All the FGDs were conducted in a prebooked room at the facilities and were audiotaped following participants' consent using audio recorders. Each FGD lasted between 45-90 minutes.

Table 1: Hospital characteristics and study population

	**Level 3 Hospital (Hospital A)**	**Level 4 Hospital (Hospital B)**	**Level 5 Hospital (Hospital C)**
**Hospital characteristics**			
Bed and cots capacity[a]	10	46	289
Number of staff[b]	35	115	262
Estimated annual deliveries[c]	1,076	5,635	9,152
Estimated annual outpatient care[c]	88,829	156,108	281,379
Estimated annual inpatient care[c]	764	7,223	14,205
**Hospital participants in the study**			
EIs	42	170	338
FGDs	3	3	3
IDIs	7	5	6
Facility level managers	*1*	*3*	*2*
Department in charges	*1*	*1*	*1*
Nursing officers	*4*	*0*	*1*
Accounting/ clerical officers	*1*	*1*	*2*
**County participants (IDI)**			**3**
Senior level managers			1
Middle-level manager			2
**National participants (KIIs)**			**15**
Ministry of Health officials			5
NHIF officials			3
Development partners			7
Notes: *Estimates for annual delivery, outpatient care and inpatient care were for the financial year July 2018 – June 2019; The outpatient total is an aggregate of both new and revisits.* ***EIs:*** *Exit Interviews;* ***FGDs:*** *Focus Group Discussions;* ***IDIs:*** *In-depth Interviews; and* ***KIIs:*** *Key Informant Interviews*			

Source: [a]Kenya Master Health Facility List [44], [b]In-depth interview with health facility in-charges of the individual facilities; [c]Kenya Health Information System (KHIS) for aggregate reporting [45].

Data management and analysis

Quantitative data from the EI was manually entered from the structured questionnaire into the Excel software by one researcher (BO), cleaned, checked for completeness, and then exported to STATA 15

For peer review only - http://bmjopen.bmj.com/site/about/guidelines.xhtml

For peer review only

for coding and analysis. The sociodemographic characteristics and the elements of quality were analysed descriptively using proportions.

All recorded FGDs were translated from Swahili to English, while the IDIs were transcribed verbatim in English. All transcripts were compared against their respective audio files by BO for transcription and translation accuracy. All the validated transcripts were imported into NVivo 12 for coding guided by the topic areas of quality of maternal healthcare. We used a framework approach to analyse the data guided by the QoC for Maternal and Newborn – a monitoring framework for network countries. [34] This approach included systematic sifting, sorting, coding, and charting data into key issues and themes. [46] One researcher (BO) familiarised himself with the data through immersion and repeatedly read and reread the transcripts. He then developed codes deductively from the conceptual framework and applied the codes to interpret segments in the transcripts that were important. The study team members (SK and SP) reviewed and discussed the initial coding framework, and any discrepancies were appropriately reconciled. The final coding framework was applied by (BO) to the data and later charted the data to allow the emergence of themes through comparisons and interpretations.

To enhance the interpretive rigour, we ensured credibility (also referred to as internal validity) through the convergence of evidence of the two methods utilised and triangulation (investigator, theoretical, and methodological) of data at the interpretive stage. [47]

Ethics consideration

This study was part of a bigger study[41] whose ethical approval was obtained from the University of Kent, the SSPSSR Students Ethics Committee and the AMREF Scientific and Ethics Review Unit in Kenya (Ref: AMREF – ESRC P537/2018). Further, we received written permission to conduct the study from the county government, and all the hospitals. We obtained written and oral informed consent from the potential participants before starting the interviews. All the study participants were presented with information sheets on the conduct of the study, the researchers involved, the purpose of the study, the right to withdraw, and measures of confidentiality ensured before they gave their written informed consent. Participants were informed that data would be reported in an aggregated format, and anonymity would be ensured in storing and publishing the study's findings.

RESULTS

The results on the quality of maternal care in this study were presented using the WHO-proposed monitoring logic model from the perspective of the implementers and the users of the policy. Results are presented in four broad domains: access to care (equitable and timely), provision of care (safe and effective), management and organisation, and care experience. A summary of the results is presented in

Table *2*.

Table 2: Summary of the quality of maternal care results

Domain	Sub-domain	Positive result	Negative result

**Element 1: Access to maternal care services under FMP (equitable and timely)**	***Minimised access barriers (cultural, financial, geographic)***	The FMP enhanced maternal care access elements (geographical, financial, or utilisation of services).	However, the facilities and HCWs were bearing the brunt of the burden of increased numbers of mothers seeking LM care (workload and burnout)

			There was an altered perception among women, leading to a preference for higher-level facilities.
	Additional maternal determinants of care and the timeliness of care	The distance to the hospital was perceived as normal (okay for the patients) and the preferred choice of transport to the facility was public transport	There was a longer waiting time for the initial visit by the pregnant women due to the enhanced ANC package of the FMP.
		All the three hospitals had a proper waiting area.	
		There was a positive perception about the time to seek care and the waiting time.	
	Provider availability		There were problems of struggling to employ specialists and other HCWs staffing challenges.
**Element 2: Provision of care (safe and effective)**	***Functional referral system***		Fewer women are being referred, but they have a better perception of services received during referral.
			The lack of equipment was the main reason for referral, and most women sought their own referral means from the hospital.
	Safety	Because of the policy, the facilities were managing complications better	HCWs were reducing the time they allocate per mother.
**Element 3: Management and organisation**	***Availability of essential physical resources***	The policy has improved the availability and standards of equipment and supplies.	Despite progress, some infrastructure, commodities, and supplies are still a challenge to some facilities.
		The facilities had improved infrastructure due to LM.	
		Enhanced facility resources and facility characteristics.	

For peer review only - http://bmjopen.bmj.com/site/about/guidelines.xhtml

	Competent and motivated staff	Mothers have a strong positive perception of healthcare delivery characteristics by the HCWs.	There were some causes of demotivation and dissatisfaction among HCWs.
		Nurses are multitasking and handling many roles amidst the challenge of human resources.	
		HCWs are adequately motivated to work despite the challenges.	
		HCWs' source of motivation was more than just money.	
	Monitoring and continuous quality improvement	Nurses monitor the quality of care provided through partographing and charting	
		labour progress, though they face challenges.	
**Element 4: Experience of care**	***Effective communication with the patients***	Mothers perceived and experienced the positive interpersonal qualities of the HCWs.	Inadequate preparation for birth by the HCWs.
		Mothers were happy to have received information about emergency/ procedures and training on breastfeeding, family planning, and baby care.	The lack of proper education and communication on expectations.
	Respect and dignity		Food was perceived as inadequate in some hospitals.
			There was over-crowding and bed-sharing, leading to a lack of privacy (congestion), and a lack of essential equipment and supplies, altering the quality of care.
	Emotional support		Women were experiencing physical, verbal, and emotional abuse.
			Some mothers experienced a lack of attention/care, negligence, and unhygienic practices from the HCWs and support staff.

Element 1: Access to maternal care services under FMP (equitable and timely)

For peer review only - http://bmjopen.bmj.com/site/about/guidelines.xhtml

Minimised access barriers (cultural, financial, geographic)

The FMP enhanced maternal care access elements (geographical, financial, or utilisation of services). For instance, due to the policy, there was an increase in the utilisation of maternal services (delivery and ANC). Findings from EIs showed that most mothers across the study sites (99.09%, n=545) visited a hospital for maternal health services during their pregnancy (Appendix 1). Further, IDI showed that more mothers (than previously) were confident in seeking skilled services rather than remaining at home.

> *'…mothers who could not come, now they are coming. And there is also a change in the number of deliveries we used to have before and now'* – ***(R009, Nursing officer).***

Equally, the respondents noted that with the enhanced identification strategies for the mothers, the FMP saw increased access to services among vulnerable populations such as street children, orphans, and adolescents. Besides, they averred that there was enhanced equity and financial access to the services by the women as those in the rural and urban areas received uniform services for free.

However, the facilities and HCWs were bearing the burden of increased numbers of mothers seeking LM care. As noted by most respondents, facilities were bearing the brunt of the increased number of mothers due to LM, which resulted in space shortages and increased workload. The workload was further exacerbated by the nature of work in the public facilities where the HCWs had no choice but to serve the mothers and meet the required utilisation targets. However, the facilities were working way beyond their abilities to manage the workload, and it resulted in HCWs experiencing some burnout:

> *'We work extra hours…you will find each care provider is serving more than they should, so the issue of burnout is also coming up'* – ***(R019, Facility Level Manager)***

There was an altered perception among women, leading to a preference for higher-level facilities. There was an increased workload in higher-level facilities caused by the mother's perception of there being specialist health care professionals that the lower-level dispensaries or community centres lack. As a result, the women believed that higher-level facilities had a higher chance of dealing with complications than the lower-level hospitals:

> *'…sometimes you ask them, "Why have you decided to come here?" "Because here, people who will attend to me are qualified."…But they say outside there, anybody can attend you.'* – ***(R014, Nursing officer).***

Additional maternal determinants of care and the timeliness of care

There was a positive perception about the time taken to seek care and the waiting time. A majority of the women visited a public facility (92%); and had a positive perception about the time taken to the facility and the distance to the hospital. Women who visited hospitals A (45.24%), B (51.18%), C (46.75%), and overall (48.00%)) noted that they took 30 minutes to 1 hour to seek delivery services and they perceived the time to be short (Appendix 1).

A majority (61.64%) perceived the distance to the hospital was normal (okay for the patients), and the preferred choice of transport to the facility was public transport (40.73%) (**Error! Reference source not found.**). Also, all three hospitals had a proper waiting area. While most of the women were happy

For peer review only - http://bmjopen.bmj.com/site/about/guidelines.xhtml

with the time the facilities were being opened and perceived the waiting time before being attended to as short (43.09%) (**Error! Reference source not found.**).

There was a longer waiting time for the initial visit by the pregnant women due to the enhanced ANC package of the FMP. The initial ANC profile included blood tests (for haemoglobin levels, blood group, rhesus, serology), Screening for tuberculosis, HIV testing and counselling, urinalysis, preventive services (such as deworming, intermittent preventive treatment for malaria, iron and folate supplementation) and prevention of mother to child transmission. All these were done at the same laboratory as other patients in the hospitals; hence, they had to wait for longer to get results:

> *'…for the first visit [they] will report here at 8:00[am] and…get out of this place as late as 3:00[pm]…because when they come…if it's lab everybody is there, the people who are coming for outpatient services are queuing there [too]…the rebate for the first visit [ANC]…covers up a lot' –* ***(R002, Clerical Officer).***

Provider availability

There were problems of struggling to employ specialists and other HCWs staffing challenges. The facility in-charges noted that they had a challenge of hiring specialist nurses to take care of the growing numbers, which had been exacerbated by the lack of specialised units:

> *'…we could not set up a neonatal ward [for lack of] a neonatal nurse...[yet] we get so many babies, and with that influx, we could still get some babies...' –* ***(R020, Facility level manager)***

One in-Charge noted that while the facilities had installed an ultrasound machine to meet the needs of the pregnant mothers, there was a gap in trying to identify the person to operate it and sustainably pay the staff.

The staffing challenge, particularly in the lower-level facilities, was hard to deal with because of the rules of staffing where, despite the high number of mothers, the number of staff cannot go beyond a certain number:

> *'…I think it's not because of Linda Mama, I think it's because of how it has been, we have been a level 3, although they said they would add us people. But you see they cannot exceed the number of staff in a level 3. If it were a level 4, they would increase.' –* ***(R007, Department incharge)***

Element 2: Provision of care (safe and effective)

Functional referral system

Fewer women are being referred, but they have a better perception of services received during referral. While referral of emergency cases is essential in preventing complications, results from EI showed that only 10.73% (n=59) of all the women interviewed in the EI, had been referred for additional care. Most had been referred from level 3 facilities (n=26), using an ambulance (n=22) or public means (n=15), and were mainly accompanied by their husbands (n=27), relatives (n=23) or health workers (n=21) either as an individual or both at the same time (Appendix 2). A majority of the mothers' companions had knowledge of emergency management (n=47), were allowed to stay in the hospitals (n=33) and were warmly received at the hospitals (n=19) during the referral (Appendix 2).

For peer review only - http://bmjopen.bmj.com/site/about/guidelines.xhtml

The women in the FGDs perceived that the maternal services provided by the mothers had improved because of the LM policy, leading to a reduction in referrals:

> *'**R3:** I can say the services are good because nowadays we don't run to [referral hospital] the way we used to. So, this hospital has been good, it has been helpful to us.'* – ***(Woman in FGD003).***

The lack of equipment was the main reason for referral, and most women sought their own referral means from the hospital. From the EI, the referred mothers noted that lack of equipment, theatre, NBU and blood (n=16) were the leading cause of referral, followed by foetal distress (n=7) (Appendix 2).

Whereas HCWs indicated that the county and facilities provide some form of referral transport for mothers, the referred mothers reported seeking their transport means for referral. These mothers perceived this to be dangerous for their health and safety and expensive, especially in unplanned emergencies.

> *'**R5:** …they [health workers] told me there's no vehicle, and they insist, "Look for a vehicle quickly so she can be referred" …now to do it fast and you don't have money…I really suffered; R8:…if a mother delivers now, [and]…is going to [a referral facility] and you know the road there is not good and someone has been stitched up down there [episiotomy]…when going there the stitches might be undone…'* – ***(Women in FGD009)***

Safety

Because of the policy, the facilities were managing complications better. HCWs and hospital administrations acknowledged that the policy improved the facilities' management of complications. The policy objectives incentivised them:

> *'…for example, she [patient] comes up with a chronic infection, which means the administration will spend more money buying an expensive drug for her. But you see, the moment she comes on time, early enough, she knows, "I went to the clinic, I was told I cannot deliver normally." She will come here on time. So, she will be told, "The moment you have reached 40 weeks, go to the hospital," she will be here. We do her C-section very safely; it is very simple she goes home. NBU decongested here…also the chorioamnionitis are no longer there.'* – ***(R012, Department Incharge)***

HCWs were reducing the time they allocate per mother. Given the workload that the HCWs were facing, they were reducing the time they allocated to providing each mother with care, and even some lower-level facilities were sending away mothers for they had higher numbers of patients: *'Owing to the fact that the patient numbers are higher than the health workers, the burden on the health worker is greater. Meaning the time allocated per patient is less than required'* – ***(R005, Facility Level Manager)***

Element 3: Management and organisation

Availability of essential physical resources

The policy has improved the availability and standards of equipment and supplies. With the help of reimbursements from the free policy, the facilities reported to have had improvements in the availability of supplies and medical equipment. In fact, the facilities have kept reordering supplies to keep up with the demand:

For peer review only - http://bmjopen.bmj.com/site/about/guidelines.xhtml

'…we've not actually gone out of stock. But you find we have to keep reordering because the demand is more.' – ***(R020, Facility Level Manager)***

Further, it was shown that with the availability of equipment and supplies, the HCWs did not have to utilise substandard care or equipment. For instance, one facility has shown how they had now departmentalised the sterilisation process of the equipment rather than using the hospital steriliser. With this came the availability of delivery packs, and they are no longer using ordinary blades as before:

'…we have so many like delivery packs which we used not to have. Sometimes we used to…. use a blade instead of a delivery pack or the scissors because these things were not there…. There are people who are employed to cater for washing those things…and take…them [to] utility for preparation for next use.' – ***(R014, Nursing Officer)***

The facilities had improved infrastructure due to LM. Some facilities had used the reimbursements from the policy to improve infrastructure such as theatre and ultrasound areas. Additionally, some were expanding their buildings to reduce congestion. For instance, one facility had been able to complete a section of an incomplete building and transfer mothers to it from the congested postnatal ward:

'…when our mothers are many in this maternity [in facility C]…those without complications or those who had delivered yesterday, we transfer them to that department, so there is that decongestion. And we have another building there, the reproductive health, it is only that it is not yet over [complete]…but now the patients who are being attended…were transferred to that department and…we got the extension.' – ***(R014, Nursing Officer)***

Other facilities even renovated older buildings that were no longer in use and converted them into maternity clinics to ease congestion. For instance, in facility B, one building constructed five years ago to be a mortuary and was only being used to store patients' records, has now been refurbished and is used as an outpatient clinic. The downside was that the mothers had a negative attitude towards it as they believed it was still a mortuary.

Additionally, the policy reimbursements were helping facilities to meet their essential services that were critical in easing the burden of work. As noted by HCWs, they could incentivise mothers by using elements such as transport that would help improve quality. However, with more patients came more workload:

'…sometimes that money will help to fuel the vehicle and…to maintain the ambulance…[and] sometimes it can support…staffs to go for seminars and…to conduct those in-reach…and also outreach services' – ***(R008, Nursing Officer)***

'….in a way it's a pusher to more quality service to the client…because you want…to attract more…because the more, the better. But…that also has brought the issue of us bursting through the seams.' – ***(R019, Facility Level Manager)***

Enhanced facility resources and facility characteristics. The women in the EI ascertained that there was an enhancement of the resources in the facilities due to the policy. The facilities were shown to have adequate waiting and examination rooms (51.60%); adequate hand washing facilities (91.82%);

For peer review only - http://bmjopen.bmj.com/site/about/guidelines.xhtml

adequate bathing facilities (67.46%); adequate toilet facilities (71.45%); well-suited equipment for detecting women's problems (90.91%); had an adequate number of staff (76.37%) who are well suited to treat women (96.55%); and had an overall clean environment (93.45%). However, the mothers showed some concern about the adequacy of the facility providing clean drinking water, as indicated by 46.18% of mothers.

Despite progress, some infrastructure, commodities, and supplies are still a challenge to some facilities. Some respondents noted that some facilities still have inadequate medical equipment (such as ultrasounds), space and supplies. The lack of these basic elements, such as a basic laboratory, was demotivating the women from using the services in the hospital and preventing HCWs from completely following up with the mothers as they would have wished to.

> *'…we don't have a very vibrant laboratory…as a clinician, I believe you want the patient tested, drugs availed, that patient will not come back to you after two days [said with wry humour]. You can give them a prescription, and they tell you they bought half a dose because they didn't have money, now, how will you help them? You see, it demotivates…… Yes. Even the ultrasound, the scans, we don't have the scans, so they have to do the scans outside [the facility]…About the [ward] it's not an ideal labour ward. We don't even have an ideal resuscitaire, you know, the improvised one?...you have to be extra cautious not to shake that thing, so the heater falls on the baby. Imagine, you have three mothers delivering, and you deliver as you put there...In the process you can burn those babies as you go to pick the other one...so, you have to be extra cautious... Even IPC [infection prevention and control] becomes an issue.'* – ***(R018, Facility level manager)***

The noted challenge regarding the supplies was that the county government was focusing on improving infrastructure, which was visible to the women, and perceived it as a better investment, rather than supplies and medication. The HCWs posited that the medication posed the biggest headache, whose potential cause was the drug ordering protocol. The facilities had to wait for a certain number of days before receiving top up for their orders:

> *'…there is a protocol…because like our drugs are ordered through KEMSA for a certain period, by any chance those drugs are not enough…they get finished before that period, we have to wait for the other order. But usually, in a hospital like ours [high-level facility], sometimes we are given extra money like miscellaneous where you can purchase emergency. But even when you purchase emergency like drugs, we are able to purchase a start dose or a prophylaxis, for continuity, you find now you have to involve maybe the patient.'* – ***(R020, Facility level manager)***

Competent and motivated staff

Mothers have a strong positive perception of healthcare delivery characteristics by the HCWs. A majority of the mothers in the EI had a positive perception of the healthcare delivery characteristics. For instance, 95.27% perceived that the staff examined pregnant and postpartum women well; 95.45% noted that the staff were very capable of finding out what is wrong with mothers; 59.64% noted that staff prescribed drugs that are needed and that the drugs supplied by the health facility were good (58.37%) and the mothers could obtain the drugs from health facility easily (67.27%) (Appendix 4). In addition, 71,27% perceived that they received adequate Information on danger signs of delivery and postpartum (Appendix 4). Interestingly, 79.82% perceived that the facility provided privacy during vaginal examination and delivery and 84.70% believed that the procedure they received during ANC and delivery felt very much necessary (Appendix 4).

For peer review only - http://bmjopen.bmj.com/site/about/guidelines.xhtml

Nurses are multitasking and handling many roles amidst the challenge of human resource. Nurses, especially in the lower-level facility, were shown to be going over and above in their work, covered both night and day shifts in addition to handling other hospital consultations at night, and still accompanied the pregnant women during referral. Despite the tasks being their roles, the constrained number of nurses was making the staff rotation allocation challenging, and hence, they had to multitask amidst the challenges.

Besides, because of the challenges of the increased workload from the LM policy, even the nurses in charge of both department and hospital administrations were forced to do the actual hands-on nursing practice rather than just stay in the office doing administrative work to ensure that the services are timely provided. Also, the nurses in the maternity wing asked for help from other departments when the work became overwhelming:

> *'…there is also the issue of shortage. Like today, we are so many, but at least we have covered all areas. But other times we report like three people, so…we have to work here and go to that place [to work in the wards]'* – ***(R007, Department in-charge)***

> *'We call help from other departments when it's so much.'* – ***(R001, Department in-charge)***

HCWs are adequately motivated to work despite the challenges. The HCWs reported being motivated to work more because they perceived that the more efforts that they put into providing service, the more the LM reimbursement funds the facility would make, which would subsequently translate to better services and additional hands (through locum nurses):

> *'…the policy of Linda mama has motivated the staff. At least we know that if you put more effort, there will be more funds on the facility, we will get more commodities, we will be compensated for escort [referral] and lunch…it will be more comfortable for us.'* – ***(R003, Nursing Officer)***

The hospital in-charges noted that despite the high workload, they feel that the HCWs are motivated and that they presented a perfect picture during supervision. For example, they noted that some were even comfortable running the wards alone without the support of other nurses and forfeiting their lunch time:

> *'…they go overboard [HCWs]…you would find two nurses on night duty, conducting 15-17 deliveries…alone. And finding this nurse has to monitor this mother from admission, delivery and postnatal and also the baby, you find they go overboard…like our nurses in maternity, they would not even break for lunch. They would wait until now the shift is over.'* – ***(R020, Facility level manager)***

Some mothers reported that the HCWs served them even when it was not their working shifts, which signified dedication to work:

> *'**R4:** I came here at 2:00 pm, and I got a doctor who was on the morning shift and the other one was changing. So, I told him to serve me, I wanted to deliver. He dressed in a hurry and came to help me.'* – ***(Woman in FGD003)***

In fact, the other cadre of HCWs, such as department clerical officers, noted that amidst the challenges, they are working beyond the stipulated hours either to support the provision of LM services or to work on the batching of the claims and ensure that the hospitals receive timely

For peer review only - http://bmjopen.bmj.com/site/about/guidelines.xhtml

reimbursement. However, they faced a challenge with inadequate and insufficient infrastructure (such as computers to ease work) and salaries. However, they perceived that they knew how to plan their days and work despite the challenges.

HCWs' source of motivation was more than just money. Some of the factors that the HCWs indicated as a source of motivation, rather than monetary values, were the kind acts and listening ear of the county administration and facility in-charges. For instance, in one facility, the department in charge felt that the administration provided them with a listening ear and acted on their grievances, including renovating the theatre and expanding the admission area. Others also felt that it resulted in the provision of adequate equipment and supplies to the facilities without having to improvise the old equipment:

> *'…at least we are listened to when we at least raise something…at least we get better service operating because of that. I mean theatre…was moved from here the squeezed area to that place, and then there wasn't bed, it was brought.'* – ***(R001, Department In-Charge)***

> *'…once in a while, we call them, have breakfast meetings with them, listen to their issues, discuss with them'* – ***(R016, County Senior Level Manager)***

The other source of motivation was that HCWs were happy when their burden of work was eased and department in-charges were doing so by employing additional people on locums, providing training opportunities, and recognising them for risking their lives at night during referrals to other facilities. Further, the nurses felt that they were involved in decision making and they perceived that it gave them a voice to raise an opinion on how the work needs to be done:

> *'So that one I see at least they could have involved us the people on the ground'* – ***(R014, Nursing Officer)***

There were some causes of demotivation and dissatisfaction among HCWs. For instance, HCWs noted that they felt inadequately remunerated despite the increased workload from the policy. With the workload, others felt that they had to multitask (for instance, handle referrals at all hours of the night and still had to come back to the facility after referral to carry out their duties which were waiting for them, and which they felt they were not adequately motivated for):

> *'We are underpaid, yeah let me say that without fear because we do a lot of work. You see like the time you came into the office; I was so buried there. I have been sitting there since 7:30 am'* – ***(R011, Clerical Officer)***

Similarly, the in-charges of the maternity departments, who were also HCWs, noted that the lack of timely reimbursements from the LM policy demotivated them. With such delays, the in-charges were having a strained working relationship with the hospital suppliers and even banks:

> *'You are doing your services, and you are claiming, but you are not getting the benefit of your work, so it renders even demoralising the people [HCWs in] the maternity…the same might demoralise even the suppliers who do supply us with the goods…some of them do cut off deals with dealing with the facility. Because we do pay them very late, and sometimes, they attract interest in their banks.'* – ***(R006, Nursing Officer)***

For peer review only - http://bmjopen.bmj.com/site/about/guidelines.xhtml

Monitoring and continuous quality improvement

Nurses monitor the quality of care provided through partographing and charting labour progress, though they face challenges. The nurses showed awareness of proper documentation of labour progress using a partograph to enhance quality care. However, they noted that they sometimes faced additional scenarios (presentations/ conditions from the patients, e.g., those from referrals or mothers who came in at the second phases of labour and delivered within a few minutes of admission) that they did not know how to document.

> *'although once in a while a file maybe there is a problem, but they try…..because you know a partograph is very important…I know maybe you have found challenges in those partographs when you were going through.'* – ***(R007, Department In-Charge).***

Despite the challenges, the nursing in-charges and facility managers were organising additional education to staff on the pregnant women monitoring processes. The university students, who were posted to the facilities for training, or even nurses who had had more recent training, were tasked to provide additional education to the nurses as they had more recent knowledge.

Element 4: Experience of care

Overall, a majority of the mothers (84.2%) from the EI were completely satisfied with the services they received (hospital A (85.1%), B (80.9%) and C (85.2%) were completely satisfied with the services provided). A higher proportion of mothers in hospital C (74.4%) than B (66.7%) and A (74.1%), would consider future delivery in the same health facility (Figure 2).

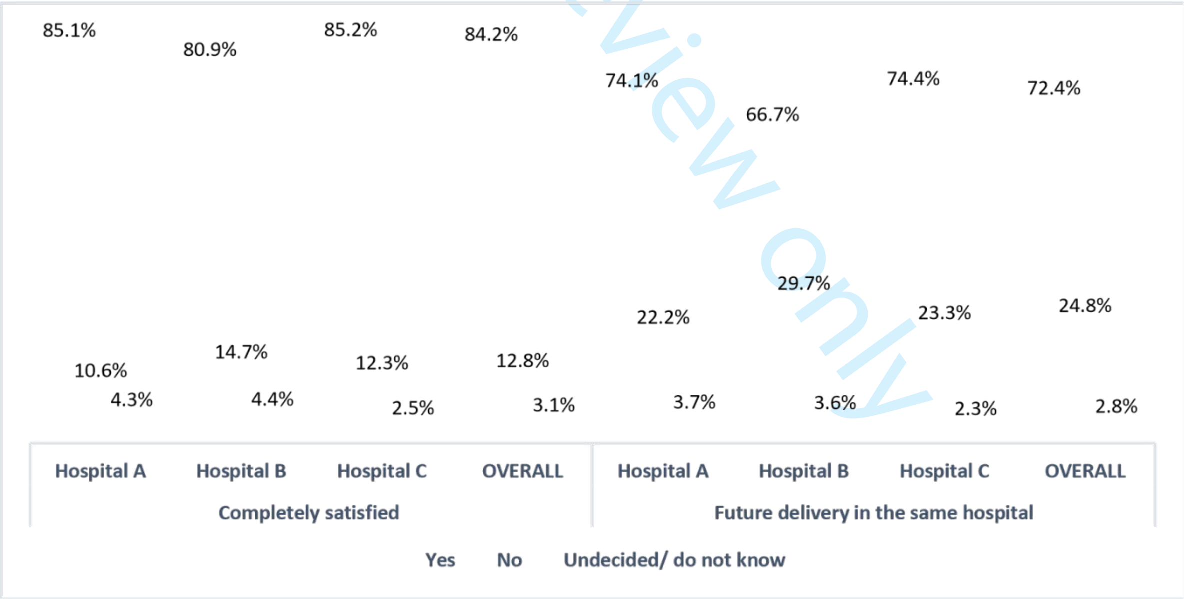

Figure 2: Overall satisfaction and future delivery

Effective communication with the patients

Mothers perceived and experienced the positive interpersonal qualities of the HCWs. A majority of the mothers in the EI had a positive perception (agreeing and completely agreeing) about the HCWs as being very open (94.34%); compassionate (90.58%); respectful (95.46%); devoted adequate time to the mothers (94.18%); and are very honest (92.00%) (Appendix 4). Some mothers noted that the HCWs

For peer review only - http://bmjopen.bmj.com/site/about/guidelines.xhtml

were empathetic, friendly, and reassuring. They appreciated the additional good treatment and sacrifices the HCWs made, such as warming food and additional support (such as bathing the baby and changing bedsheets and stained beddings) following the exhausting birth experience.

Some mothers appreciated being given priority in treatment, especially during emergencies by the doctors. In such circumstances, the firmness and decisiveness of the nurses were also perceived positively as being intent on preserving the lives of both the mother and baby. One mother was particularly impressed with the doctors who called for assistance in emergency scenarios when they were not able to handle them at the time:

> *'**R1:** when I came once I got a certain doctor and I think there was an emergency, and I was forced to wait but I did not take offence because…he called another doctor who came here and I saw they have experience because they just serve you.'* – ***(Woman in FGD003).***

Mothers were happy to have received information about emergency/ procedures, and training on breastfeeding, family planning, and baby care. Some mothers highlighted that since some doctors explained to them the medical procedures they were to undergo; they were able to relieve some anxiety around birth especially:

> *'**R2:** The doctor was good, he told me how it [procedure] would be done, and I was good.'* – ***(Woman in FGD004).***

The nurses supported the mothers during breastfeeding, taught them how to breastfeed and even encouraged those with difficulties. Some hospitals even went further by demonstrating to the mothers through *YouTube* videos on the procedure of breastfeeding which they perceived as very useful and helpful. The facility in-charges acknowledged that they trained and empowered the nurses with breastfeeding knowledge to ensure that they in turn train the mothers:

> *'And once this nurse trains in the breastfeeding, she'll go back, we make it as a duty for her to be educating the mother on those…on breastfeeding'* – ***(R020, Facility Level Manager).***

Besides breastfeeding, the mothers acknowledged being taught about family planning, how to wash the baby's cord, and what to do if the baby faced some complications, which they considered reassuring.

Inadequate preparation for birth by the HCWs. Some HCWs were perceived as not being well prepared to handle the birth of the baby, given that they never had the birth equipment readily laid or that some materials and supplies were not readily available. This ultimately resulted in birth complications such as amniotic fluid aspiration.

The lack of proper education and communication on expectations. Some mothers felt there was no clear communication on the immediate care after delivery, which created a knowledge gap and potentially made mothers make mistakes with medications that resulted in medical emergencies. For instance, one mother indicated:

> *'**R6:** For my child there was a time I put the Hexi-cord [cord cleaning medication] on their nose. I did not know; I asked my husband to pass me the medicine at night thinking it was a nose drip. So, we thought that was it and we administered to him, we were forced to bring the baby here at night.'* – ***(Woman in FGD001).***

For peer review only - http://bmjopen.bmj.com/site/about/guidelines.xhtml

Some HCWs were perceived as not being reassuring and unable to provide mothers with the expected reassurance:

> *'**R12:** "We have examined you; the baby is not close." You know, sometimes you feel the baby is close, and when it's time to deliver, many doctors and nurses came and told me, "Why are you disturbing us, you are standing on the floor. Climb the bed." I could not climb. They said, "We are referring you to [a referral hospital]." Now I said, oh my god what will I do? At that time, they started to insult me and told me, "Come here, you are going to deliver in the ward."'* – ***(Woman in FGD009).***

Respect and dignity

Food was perceived as inadequate in some hospitals. Some mothers revealed that despite having a good birth experience in the labour ward and not paying anything for the delivery, the food provided particularly by the support staff post-delivery was inadequate, untimely, and unwholesome. Some mothers in some facilities highlighted that appetence for food could sometimes last for a whole night post-delivery and thus they resorted to having their relatives and family bring them food.

> *'**R2:** I didn't pay anything, though their food is too little for a pregnant woman. It's true, it's too little, a mother has delivered, that food…and then they serve it very early, when it reaches 9pm you are hungry again…Yes, I had to call home [for food] because I felt weak. R7: There was a day I stayed here without food the whole night. I wasn't given.'* – ***(Women in FGD009).***

However, the administration revealed that the instance of food inadequacy may have been caused by the support staff who, despite the facility planning for adequate food for the whole hospital patients, may have rationed the food further. Despite the inadequacy of food, some mothers acknowledged that the food was actually good:

> *'**R3:** Yeah, it was good, I ate good things, and even the bathroom was clean. The services there are good.'* – ***(woman in FGD003)***

There was over-crowding and bed-sharing leading to a lack of privacy (congestion), and a lack of essential equipment and supplies, altering the quality of care. Congestion in the maternity department because of the FMP was a crosscutting theme especially in the higher-level facilities. The lower-level facilities equally faced an increase in the number of mothers particularly for ANC and delivery, but the mothers did not share beds:

> *'**R3:** but the problem I found here is congestion…. the first three hours [following CS] … I slept on a bed alone, after three hours we were two people on the bed. And from there the room we were taken too we would sleep four people with children, six people like that in one bed…. because I left there with a back problem because I cannot sleep, you are forced to sit, you sit for the child to sleep.'* – ***(woman in FGD005).***

Nonetheless, the hospitals gave bed priority to mothers who had had CS over normal delivery and were allowed to sleep on the bed alone in space permitting incidences in addition to having a special monitoring room. In contrast, mothers who had given birth normally, were forced to share beds with other mothers or sleep on the floor with only the babies sleeping on the beds. The congestion in the public facilities forced the mothers to seek care elsewhere.

For peer review only - http://bmjopen.bmj.com/site/about/guidelines.xhtml

Also, despite there being the FMP, the lack of basic essential equipment and space was also noted to be a key driver to poor QoC even in maternal and child health clinics for PNC:

> *'Go to MCH… and see how babies are weighed naked outside, in this harsh weather. It is at times very cold in the morning but what do we do, we have to weigh them….but we are glad that we are still able to offer services'* – ***(R018, Facility Level Manager).***

Emotional support

Women experienced physical, verbal, and emotional abuse. Some mothers experienced both physical and verbal abuse from HCWs and support staff. The abuse was exacerbated by the lack of clarity in communication with HCWs. For instance, one woman reported that the nurses had slapped her for being stubborn and uncooperative during birth, another woman mentioned that the nurse had tried to suture her episiotomy without using anaesthesia, and still another received abuse in return from either support staff or HCWs for requesting support:

> *'**R6:** I was slapped here…. for being stubborn; R3: you see someone is still in pain, they do not inject you with anaesthesia and they want to stitch you. Things like that are not good, this is also a human being, and they still feel pain. R5: I saw someone who had gone through a CS, and they told the nurse, they wanted to rise up, you know there is pain while rising up…but I saw her telling that nurse to help her get up, I saw [heard] the nurse insult her and I did not like that'* – ***(Women in FGD001)***

Equally important was one mother's testimony showing how she was wheeled to the theatre in a rather uncaring manner that lacked dignity:

> *'**R5:** What I saw, what he did to me, when I was experiencing labour pains, I was told to go to theatre, and I told him I cannot walk. He pushed me like a cart up to the theatre. I told him I could not walk; he pushed me like a lorry.'* – ***(Woman in FGD008)***

Some mothers experienced a lack of attention/care, negligence and unhygienic practices from the HCWs and support staff. For instance, in one case a doctor was shown to have forgotten to remove cotton wool used in packing blood after delivery:

> *'**R6:** Like in my case they did not remove that thing [cotton wool] and then I went home with it.'* – ***(Woman in FGD002)***

Additionally, some mothers perceived that some HCWs were not giving them and their babies proper attention while attending to them and they felt ignored. For instance, one respondent whose baby required medical oxygen felt a lack of support:

> *'**R6:** the baby came out fine. But I saw that by the time the nurse received him, he wasn't breathing well and then the nurses did not care because when I woke up after six hours I had to go look after my baby, when the oxygen came out, I would put it back, I changed everything. So, this time round I did not like them.'* – ***(Woman in FGD006)***

Some mothers were subjected to unhygienic practices by some HCWs, including being examined on an unclean bed previously used by another patient without wiping or being left unattended for long:
*'**R4:** Another thing that I didn't like there, you are examined on a bed that someone else had been examined on and it is damp. It wasn't good. Like for me I was examined on a bed that had some liquid substance; **R9:** I delivered at [a referral hospital]; I didn't like their services at all. Because when I*

For peer review only - http://bmjopen.bmj.com/site/about/guidelines.xhtml

delivered, I was cut down there [episiotomy] and the doctor left me for 30 minutes. On coming back, he stitched me with all that dirt, so I was not happy at all with their service.' – ***(Women in FGD009,).***

Some support staff also exacerbated the unhygienic practices of the mothers. For instance, one mother noted:

> *'**R6:** when I delivered here, I was asleep, when I woke up around 6.30. I found they [support staff] had opened windows as they wanted to clean. If you had put your bag on the floor, they ask you to pick it up and put it in bed and that bed is where you place the baby, and the ground is dirty.'* – ***(Woman in FGD001).***

DISCUSSION

To our knowledge, this is the first study to examine the quality of care across the continuum of maternal care (antenatal, perinatal, and postnatal care) under the free maternity policy in Kenya.

Our findings show that the LM policy has reduced geographical access barriers by harnessing more private sector and faith-based facilities to enhance service provision. Furthermore, it has eliminated financial access barriers through the incentives of free maternal care and increased utilisation of maternal services (more mothers seek SBA; hence, reduced home deliveries). These findings align with results from systematic reviews of maternal services under different free maternity policies, which showed increased maternal (ANC and delivery) services after removing user fees. [48 49] Dossou et al. [50] also showed a systematic increase in CS services after implementing the CS policy in Benin because of utilisation incentives. However, the reviews showed that the utilisation patterns under free policies were marred by geographical and temporal fluctuations in use, which differs from our study.

Further, despite the policy enhancing access, the facilities were using additional approaches and incentives to attract mothers, leading to a difference in perception of the services provided. The finding on factors leading to the choice of the delivery place is not new, as other authors have highlighted the difference in the preference for private or public facilities thus influencing perception. [51-53] In fact, in a recent FGD with women in Nairobi's informal settlements in Kenya, exploring their experiences of the quality of maternity care under LM, Oluoch-Aridi et al. [54] present the facilitators and barriers to choosing health facilities, which are all similar to the findings of this study. Interestingly, the choice of delivery site was influenced by several factors that are not necessarily related to LM, such as personal choice, previous experience or treatment, and access, as shown in other studies[4 55] or health system factors. [56] This highlights a key gap because it raises the question of whether LM has influenced the choice of hospital for delivery. Escamilla et al. [57] showed that the need for free services in Kenya had influenced women to bypass nearer facilities for farther private facilities that offered free care; which is similar to the findings from Sierra Leone by Fleming et al. [58]

Interestingly, while there was an increase in the utilisation of free maternal services, the facilities and HCWs bore the burden of increased numbers of mothers seeking LM care. HCWs were shown to be working beyond their capacity to provide care and experiencing burnout. Several authors have shown that following the implementation of the free policy in Kenya; there was a significant increase in the utilisation of maternal services, [14 59 60] which was attributable to the removal of cost barriers to women. [61] However, because the increase in the number of mothers seeking SBA services after implementing the policy did not follow a subsequent increase in the number of HCWs, the HCWs were bound to be

For peer review only - http://bmjopen.bmj.com/site/about/guidelines.xhtml

burdened. Previous studies have shown that the perennial lack of human resources has always been a problem in Kenya. For instance, Miseda et al. [62] reveal that out of the 138,266 HCWs required to fit the MoH Norms and Standards Guidelines for service delivery, only 31,412 are employed at the public sector, private facilities, and faith-based organisations (FBOs).

It was also shown that the HCWs went beyond their strengths to serve the increased number of mothers well, as a way to maximise reimbursements from the LM policy, but this could cause burnout if not followed by an increased workforce, thus leading to poor QoC. Two meta-analysis studies have shown that HCWs burnout could lead to the provision of poor QoC. [63 64] HCWs are motivated by what Franco et al. [65] deriving from Herzberg et al. [66] refer to as 'hygiene factors' (determining HCWs dissatisfaction) in this case the interpersonal relationship with the county and the administration, and 'motivating factor' (determining HCWs motivation and satisfaction) in this case being listened to. However, the facilities struggled to employ specialists and other HCWs staffing challenges.

Our study has highlighted the enhanced identification strategies for vulnerable populations (such as street children, orphans, and adolescents) that had initially been excluded from the policy on paper and are now using the policy. The findings align with the results of implementing the Safe Motherhood programme in Nigeria (Abiye initiative), which equally showed that removing user fees, particularly for the most vulnerable population, enhanced access and utility of service. [67] However, in a different study in Kenya, researchers showed that the enhancement of the reach of the vulnerable population was mainly done by HCWs who, bound by ethics and professionalism, provided FMP services to those excluded from the policy, such as foreigners, and those without IDs, such as street children who had no parents, refugees without IDs, or schoolgirls who were underage and pregnant. Hence, there is a need for official policy correction. While our results further show that there has been enhanced equity and financial access to the services by the women as those in the rural and urban areas received uniform services for free, in Benin, the CS policy exacerbated the inequalities as the policy reached the predominantly rich, exacerbating social exclusion. [50]

Besides, from our findings, there is a positive perception of the policy despite the longer waiting times, particularly in the initial visits where mothers are accessing ANC additional benefit packages that were not in the previous policy. In contrast, a mixed-method study in Nigeria showed that mothers were dissatisfied with the waiting time under the free policy, but the authors did not link it to any particular service. [68]

A rather interesting finding is the mothers' preference for higher-level facilities due to the perception of better services. Higher-level facilities are significantly burdened due to LM policy, leading to a ripple effect (where the facilities are left with a resource gap, as they use more resources to meet the mothers' specialised needs and manage deliveries that can be done at the periphery). However, it could also be argued that having more mothers in higher-level facilities means more claims and reimbursements. However, literature has attributed this preference to factors such as cleanliness, interpersonal skills, and other perceptions of better services; [69] and not the LM policy. A discrete choice experiment in Nigeria showed that the women chose to give birth in places with good condition of the health system, and absence of sexual, physical and verbal abuse, and that an unclean environment of birth without privacy and unclear user fees policy drove the women away. [70] The mother's choice of higher-level facilities has led to QoC concerns such as indifference in the treatment based on the type

For peer review only - http://bmjopen.bmj.com/site/about/guidelines.xhtml

of delivery and parity (partly because of overburdening higher facilities and the need for prioritisation). In Kenya, other studies have shown that mothers bypass lower-level facilities due to the perception of better quality. [71 72] Same case has been shown in Sri Lanka. [73]

Interestingly, fewer mothers are being referred from lower to higher facilities than before the LM policy. While in the previous policy, complications were being referred to higher-level hospitals from lower-level health centres to seek better services, [74] it could be argued that, through the LM policy, lower-level facilities are making adequate investments using the LM policy reimbursements and are thus able to handle complications. That may nevertheless not be true as another finding in our study showed that the fewer referrals that are happening are mainly due to the lack of equipment, theatre, and NBU in the lower-level facilities. Thus, it could be that the policy confusion in the reimbursements of the services is somewhat hampering the positive quality effects of the policy. Other literature from Ghana concurs with this assumption. For instance, Witter et al.'s[75] exploration of the policy showed that the uncoordinated and unreimbursed referral strategy (particularly at referring hospitals) hampered the positive effect of the policy, while Ganle et al. [76] showed that Ghana's referral system was ineffective and the care was substandard because of a lack of critical care staff to handle healthcare emergencies.

The mothers who are referred have a positive perception of the referral process. This perception could be because the HCWs went above and beyond to provide referral elements, such as allowing the mothers to have companions at referral time and in the hospital. However, the lack of transport for referral could hamper the referral gains by either making the mother pay or risk their life looking for transport systems at the tail end of delivery. For example, Burkina Faso included transport in their subsidy policy to enhance mothers' referrals to health facilities. [77] Through its well-organised rapid response to emergency and evacuation, mothers were positively satisfied with the referral system under the policy; however, IDIs with HCWs revealed no adequate follow-up to ensure the evacuated mothers received care as intended. [78] Interestingly, Kenyan nurses under the LM policy went above and beyond to refer and follow up mothers, which was a compensatory mechanism for improving QoC.

In addition, through the LM policy, there has been some improved availability of equipment, supply, and infrastructure. The improvement could be due to the provider and in-charges using Streel Level Beureacrat tacts (such as renovations) to improve the facility to attract more mothers who are the source of reimbursement funds. However, despite progress, some commodities, infrastructure, and supplies remain a challenge. The lack of supplies, equipment, and infrastructure contravenes the WHO statement number eight on quality, which shows that positive birth outcomes rely on their availability. [35] A recent review showed that inadequacy is a global phenomenon compromising the quality of maternal care. [79] Evidently, in all the facilities, the mothers revealed that they were satisfied with the characteristics of the facilities, such as having adequate rooms, adequate hand washing, bathing, and toilet facilities; in addition to equipment well suited for detecting women's problems. As is in this study, a mixed-methods study in Ghana showed that, despite the inadequate infrastructure in the facilities and lack of basic supplies, 89% of the mothers who participated in the EI, and those in the FGDs were satisfied with the quality of maternal care during childbirth[80] as is in this study. This postulates that mothers are more concerned about the interpersonal care received and the basic

For peer review only - http://bmjopen.bmj.com/site/about/guidelines.xhtml

amenities provided if they can have live births and remain alive. The absence of or inadequacy of equipment and supplies compromises the QoC.

Equally interesting was that the good experience of care received by the women was based on the level of support provided by the HCWs and the facilities. Research shows that a good relationship between patients and HCWs could help improve trust, diffuse patients' anxieties, and create open communication. [81] The majority of the mothers in both the FGDs and the EI attributed the good experience of care to the interpersonal skills exhibited by the HCWs, such as empathy, being friendly, kindness, respect, devoting time, and honesty. The good care experiences the women receive influences their future delivery in the same facility. However, the findings could not show whether such experiences were due to LM policy, except that it incentivised the HCWs to provide FP and breastfeeding education. The finding shows that despite the challenges of the policy, the mothers appreciated and perceived the HCWs and health facility characteristics positively. This shows that HCWs have significantly contributed to the quality of provision of care, but this may not lead to improved outcomes if the technical aspects of quality are not met. Similar findings have been reported elsewhere where, for instance, in Ghana, 77% of the mothers who participated in the EI noted that they were content with the HCWs service provision as they were patient and empathetic[80] or in Ethiopia, where 79.1% of the mothers interviewed were happy with the overall services provided. [82]

The poor experience of care by the mothers hampers the technical QoC received. By sharing the beds due to overcrowding, the mothers are exposed to unhygienic practices that could eventually lead to nosocomial infection in the maternity facilities, which hampers QoC. A review of quality elements in facilities in the 14 counties in Kenya linked the introduction of LM services with poor hygiene and low privacy. [24] Such findings are expected because investments in hospital infrastructure have not subsequently followed the increase in the number of mothers utilising maternal care.
Other literature has shown similar findings in other settings with FM services. [83-85]

The other finding of poor QoC experienced by the mothers, such as lack of attention, negligence and physical abuse, has been shown in other Kenyan literature. For instance, the beneficiaries of FM services in a study in Kakamega provincial hospital in Kenya noted that the HCWs negligence and use of vulgar language were demeaning to the patients. [86] Food is an important component in the birth process and for mothers to report that the food they received during delivery is inadequate is as surprising as it is demeaning. Also, as is in this study, poor communication with the mothers or lack thereof may create an ethical dilemma, especially in contexts where patients do not consent to or are not explained for procedures. [87] Mothers should play a role in the decisions of the care provided.

A key limitation of this study is that the EIs were conducted in one county, and it is plausible that there could be varied practices across other counties. The implication of this study is that it may be difficult to generalise the findings to all the other 47 counties in Kenya. Nonetheless, using IDIs and FGDs in this study provides an opportunity to unpack the issue at hand (quality of maternal care under LM policy) within its context and be analytically generalisable. The meta-issues identified by the study are likely to be found in other counties, even though they might manifest in different ways.

For peer review only - http://bmjopen.bmj.com/site/about/guidelines.xhtml

CONCLUSION

This study has demonstrated that LM policy has provided positive results of quality across all the broad quality domains: access to care (equitable and timely), provision of care (safe and effective), management and organisation, and the experience of care. There were positive elements such as minimised access barriers (cultural, financial, geographic), timeliness of care, and provider availability that have created functional referral systems and safety, and availability of essential physical resources and competent and motivated staff. The women in the study had a good care experience, which included reception of prompt maternal services, good care for the baby after birth, teaching about birth procedures, breastfeeding, and family planning. Further, the results have shown negative results from the policy hampering maternal care, such as the lack of supplies, equipment and infrastructure, and referral challenges. Cross-cutting poor experiences from the women exist, such as overcrowding of the healthcare facilities, inadequate food supply, the lack of communication of treatment plans, and experiencing both physical and verbal abuse. There is a need to address the negative aspects of the study while strengthening the positives to achieve the SDG and UHC goals that seek to ensure reduced maternal morbidities and mortalities through access to quality service for every woman.

List of abbreviations: ANC: Antenatal care; **CS:** Caeserian Section; **FBO:** faith-based organisations; **FGD:** Focus group discussions; **FMP:** Free maternity policy; **HCWs:** healthcare workers; **IDI:** Indepth interviews; **LM:** Linda mama; **LMIC:** Low-and-middle-income countries; **LIC:** low-income countries; **MCH:** maternal and child health; **QoC:** quality of care; **SBA:** Skilled birth attendance; **SSA:** sub-Saharan Africa; **UHC:** Universal Health Coverage; **WHO:** World Health Organization

Twitter: @bonnyoyugi_snr

Acknowledgements: The authors acknowledge the data collection support of the EI data from research assistants: Rachel Murigu, Justus Miran, Billy Bortich, Valentine Olunga, Janet Moraa, Winnie Kaitany, and Shillar Jeptoo.

Contributors: BO: conceptualised the study, curated and analysed the data, and drafted the initial manuscript which was subsequently revised for important intellectual content by all authors. ZAP: reviewed and edited all the drafts. SK and SP: contributed to the design and supervised the study. All authors read and approved the final manuscript.

Funding: This study was funded by the Commonwealth Scholarship Commission, which supported BO's PhD study. The funding agency did not play any role in the analysis, interpretation of results, and manuscript writing.

Competing interests: None declared.

Patient consent for publication: Consent obtained directly from patient(s).

Patient and public involvement: Patients or the public WERE NOT involved in the design, or conduct, or reporting, or dissemination plans of our research.

For peer review only

Ethics approval: Ethical approval was obtained from the University of Kent, SSPSSR Students Ethics Committee and AMREF Scientific and Ethics Review Unit in Kenya (Ref: AMREF – ESRC P537/2018). Further written permission to conduct the study was received from the county government of Kiambu, and all the participating hospitals. Written and oral informed consent was obtained from the potential participants before starting the interviews.

Data availability statement: The datasets used in this study are available from BO on b.oyugi@kent.ac.uk upon reasonable request.

Supplementary material: The authors have supplied this content.

For peer review only

REFERENCES

1. World Health Organization. Trends in maternal mortality 2000 to 2020: estimates by WHO, UNICEF, UNFPA, World Bank Group and UNDESA/Population Division Geveva: World Health Organization; 2023 [Available from: https://www.who.int/publications/i/item/9789240068759 accessed 12 March 2023.
2. Black RE, Walker N, Laxminarayan R, et al. Reproductive, Maternal, Newborn, and Child Health: Disease Control Priorities Washington (DC): The International Bank for Reconstruction and Development / The World Bank; 2016 [Third Edition (Volume 2):[1]. Available from: https://www.ncbi.nlm.nih.gov/books/NBK361907/ accessed 16 October 2023.
3. Kyei-Nimakoh M, Carolan-Olah M, McCann TV. Access barriers to obstetric care at health facilities in sub-Saharan Africa—a systematic review. *Systematic reviews* 2017;6:1-16.
4. Gabrysch S, Campbell OM. Still too far to walk: literature review of the determinants of delivery service use. *BMC pregnancy and childbirth* 2009;9(1):34.
5. Kenya National Bureau of Statistics, ICF. Kenya Demographic and Health Survey 2022. Key Indicators Report Nairobi, Kenya, and Rockville, Maryland, USA: KNBS and ICF; 2023 [Available from: https://dhsprogram.com/publications/publication-PR143-PreliminaryReports-Key-Indicators-Reports.cfm accessed 12 March 2023.
6. UNICEF. Neonatal mortality: UNICEF; 2020 [Available from: https://data.unicef.org/topic/childsurvival/neonatal-mortality/ accessed 15 September 2020.
7. Kenya Ministry of Health. Reducing Maternal and Neonatal Mortality in Kenya: Scaling up Effective Interventions in Maternal and Newborn Health, An Implementation Plan for the period 2016 - 2018. In: Reproductive and Maternal Health Services Unit, Neonatal Child and Adolescent Health, Community Health Services Unit, et al., eds. Nairobi: Kenya Ministry of Health, 2016.
8. World Health Organisation. Trends in Maternal Mortality 2000 to 2017: Estimates by WHO, UNICEF, UNFPA, World Bank Group and the United Nations Population Division. Geneva: World Health Organization 2019.
9. Chuma J, Maina T. Free Maternal Care and Removal of User Fees at Primary-Level Facilities in Kenya. Monitoring the Implementation and Impact—Baseline Report. Washington, DC: Health Policy Project, Futures Group, 2013.
10. Janisch C, Albrecht M, Wolfschuetz A, et al. Vouchers for health: a demand side output-based aid approach to reproductive health services in Kenya. *Global Public Health* 2010;5(6):578-94.
11. Chuma J, Musimbi J, Okungu V, et al. Reducing user fees for primary health care in Kenya: policy on paper or policy in practice? *International Journal for Equity in Health* 2009;8:15. doi: 10.1186/1475-9276-8-15
12. Chuma J, Okungu V. Viewing the Kenyan health system through an equity lens: implications for universal coverage. *International Journal for Equity in Health* 2011;10(1):22.
13. Opwora A, Kabare M, Molyneux S, et al. Direct facility funding as a response to user fee reduction: implementation and perceived impact among Kenyan health centres and dispensaries. *Health Policy and Planning* 2010;25(5):406-18. doi: 10.1093/heapol/czq009
14. Tama E, Molyneux S, Waweru E, et al. Examining the implementation of the free maternity services policy in Kenya: a mixed methods process evaluation. *International Journal of Health Policy and Management* 2018;7(7):603-13. doi: 10.15171/ijhpm.2017.135 [published Online First: 2018/07/13]

For peer review only - http://bmjopen.bmj.com/site/about/guidelines.xhtml

15. Orangi S, Kairu A, Ondera J, et al. Examining the implementation of the Linda Mama free maternity program in Kenya. *The International Journal of Health Planning and Management* 2021
16. World Health Organisation. The World Health Report 2010 Health Systems Financing: The path to universal coverage. Geneva: World Health Organization, 2010.
17. World Health Organisation. Arguing for Universal Health coverage. 2013
18. Orangi S, Kairu A, Malla L, et al. Impact of free maternity policies in Kenya: an interrupted timeseries analysis. *BMJ Global Health* 2021;6(6):e003649. doi: 10.1136/bmjgh-2020-003649
19. Gitobu C, Gichangi P, Mwanda W. Patterns in maternal mortality following the implementation of a free maternal health care policy in Kenyan public health facilities. *East African Medical Journal* 2017;94(6):433-44.
20. Gitobu C, Gichangi P, Mwanda W. Causes of neonatal mortality two years before and after the implementation of a free maternal health care policy in Kenyan public health facilities. *East African Medical Journal* 2017;94(5):323-35.
21. Lang'at E, Mwanri L, Temmerman M. Effects of implementing free maternity service policy in Kenya: an interrupted time series analysis. *BMC Health Service Research* 2019;19(1):645. doi: 10.1186/s12913-019-4462-x [published Online First: 2019/09/08]
22. Oyugi B, Nizalova O, Kendall S, et al. Does a free maternity policy in Kenya work? Impact and cost-benefit consideration based on demographic health survey data. *Eur J Health Econ* 2023 doi: 10.1007/s10198-023-01575-w [published Online First: 2023/02/14]
23. Oyugi B, Kendall S, Peckham S, et al. Out of pocket payments during childbirth in Kenya under the free maternity services: Perspectives of mothers, healthcare workers and county officials. *Wellcome Open Research* 2023
24. Gitobu CM, Gichangi PB, Mwanda WO. Satisfaction with delivery services offered under the free maternal healthcare policy in Kenyan public health facilities. *Journal of Environmental and Public Health* 2018;2018:4902864. doi: 10.1155/2018/4902864
25. Owiti A, Oyugi J, Essink D. Utilization of Kenya's free maternal health services among women living in Kibera slums: a cross-sectional study. *Pan Afr Med J* 2018;30:86. doi: 10.11604/pamj.2018.30.86.15151 [published Online First: 2018/10/23]
26. Lusambili AM, Naanyu V, Wade TJ, et al. Deliver on Your Own: Disrespectful Maternity Care in rural Kenya. *PLOS ONE* 2020;15(1):e0214836. doi: 10.1371/journal.pone.0214836
27. Donabedian A. The quality of care: how can it be assessed? *The Journal of the American Medical Association* 1988;260(12):1743-48.
28. Donabedian A. The seven pillars of quality. *Archives of Pathology and Laboratory Medicine* 1990;114(11):1115-18.
29. Kruk ME, Gage AD, Arsenault C, et al. High-quality health systems in the Sustainable Development Goals era: time for a revolution. *The Lancet Global Health* 2018;6(11):e1196e252.
30. World Health Organization. The network for improving quality of care for maternal, newborn and child health: evolution, implementation and progress: 2017-2020 report. 2021
31. Creswell JW, Clark VLP. Designing and Conducting Mixed Methods Research. Third edition ed. Los Angeles: Sage publications, Inc 2017.
32. Kelley E, Hurst J. Health Care Quality Indicators Project: Conceptual Framework Paper: Organisation for Economic Co-operation and Development 2006 [cited 2018 13th May]. Available from:

For peer review only - http://bmjopen.bmj.com/site/about/guidelines.xhtml

http://thuvien.thanglong.edu.vn:8081/dspace/bitstream/DHTL_123456789/3992/1/No.%2023%20Healthcare%20quality%20indicators%20project%20%E2%80%93%20Conceptual%20framework%20paper.pdf accessed 13th May 2018.

33. Akachi Y, Kruk ME. Quality of care: measuring a neglected driver of improved health. *Bulletin of the World Health Organization* 2017;95(6):465.
34. World Health Organization. Quality of Care for Maternal and Newborn Health: A Monitoring Framework for Network Countries Avenue Appia 20, 1211 Genève, Switzerland: Department of Maternal, Newborn, Child and Adolescent Health (MCA), World Health Organization HQ; 2019 [Available from: https://www.who.int/publications/m/item/quality-of-care-formaternal-and-newborn--a-monitoring-framework-for-network-countries accessed 16 October 2023.
35. World Health Organisation. Standards for Improving Quality of Maternal and Newborn Care in Health Facilities 20 Avenue Appia, 1211 Geneva 27, Switzerland: World Health Organisation; 2016 [Available from: https://apps.who.int/iris/bitstream/handle/10665/249155/9789241511216-per.pdf accessed 8th March 2019.
36. Kenya Ministry of Health. Kenya Community Health Policy 2020-2030. In: Division of Community Health, ed. Nairobi, Kenya: Kenya Ministry of Health, 2020.
37. Kenya National Bureau of Statistics. 2019 Kenya Population and Housing Census Volume I: Population by County and Sub-County Nairobi: Kenya National Bureau of Statistics; 2019 [cited 2020 27 March]. Available from: https://www.knbs.or.ke/?wpdmpro=2019-kenyapopulation-and-housing-census-volume-i-population-by-county-and-sub-county accessed 27 March 2020.
38. County Government of Kiambu. About Kiambu County 2018 [cited 2020 27 March]. Available from: https://kiambu.go.ke/about-us/#2 accessed 27 March 2020.
39. Kenya National Bureau of Statistics, Kenya Ministry of Health, National AIDS Control Council, et al. Kenya Demographic and Health Survey 2014 Calverton, MD: Kenya National Bureau of Statistics & ICF international; 2014 [cited 2020 5th May]. Available from: https://dhsprogram.com/pubs/pdf/fr308/fr308.pdf accessed 5th May 2017.
40. Gorstein J, Sullivan K, Parvanta I, et al. Indicators and Methods for Cross-Sectional Surveys of Vitamin and Mineral Status of Populations: The Micronutrient Initiative (Ottawa) and the Centers for Disease Control and Prevention (Atlanta); 2007 [Available from: https://www.who.int/vmnis/toolkit/mcn-micronutrient-surveys.pdf accessed 3 September 2020.
41. Oyugi B. The Policy Process, Quality and Cost of Free Maternal Healthcare in Kenya: A Mixed Methods Analysis of Maternity Policy. University of Kent, Centre for Health Services Studies, University of Kent, 2021.
42. Dalinjong PA, Wang AY, Homer CSE. The operations of the free maternal care policy and out-ofpocket payments during childbirth in rural Northern Ghana. *Health Economics Review* 2017;7(1):41. doi: 10.1186/s13561-017-0180-4
43. StatTrek.com. Random Number Generator: Stat Trek; 2021 [cited 2019 20 October]. Available from: https://stattrek.com/statistics/random-number-generator.aspx accessed 20 October 2020.
44. Kenya Ministry of Health. Kenya Master Health Facility List (KMHFL): Kenya Ministry of Health,; 2020 [Available from: http://kmhfl.health.go.ke/#/home accessed 12 November 2020.
45. DHIS2. Kenya Health Information System (KHIS) for Aggregate Reporting: DHIS2; 2020 [cited 2020 16 March]. Available from: https://hiskenya.org/dhis-webcommons/security/login.action accessed 16 March 2020.

For peer review only - http://bmjopen.bmj.com/site/about/guidelines.xhtml

46. Ritchie J, Spencer L. Qualitative data analysis for applied policy research. In: Bryman A, Burgess RG, eds. Analysing Qualitative Data. New York, NY: Routledge, Taylor & Francis Group 2002:173-94.
47. Yin KR. Case Study Research and Applications: Design and Methods. 6th ed. Thousand Oaks, CA: SAGE Publications 2018.
48. Dzakpasu S, Powell-Jackson T, Campbell OM. Impact of user fees on maternal health service utilization and related health outcomes: a systematic review. *Health Policy and Planning* 2014;29(2):137-50. doi: 10.1093/heapol/czs142
49. Oyugi B, Kendall S, Peckham S. Effects of free maternal policies on quality and cost of care and outcomes: an integrative review. *Primary health care research & development* 2021;22
50. Dossou J-P, Cresswell JA, Makoutodé P, et al. 'Rowing against the current': the policy process and effects of removing user fees for caesarean sections in Benin. *BMJ Global Health* 2018;3(1):e000537. doi: 10.1136/bmjgh-2017-000537
51. Okumu C, Oyugi B. Clients' satisfaction with quality of childbirth services: a comparative study between public and private facilities in Limuru Sub-County, Kiambu, Kenya. *PLoS One* 2018;13(3):e0193593.
52. Chirdan O, Lar L, Afolaranmi T, et al. Client satisfaction with maternal health services comparism between public and private hospitals in Jos Nigeria. *Jos Journal of Medicine* 2013;7(1):1-9.
53. Khan REA, Noreen S. Household choice of public versus private health institution for maternal health-care: a case study of Bahawalpur (Pakistan). *Pakistan Journal of Commerce and Social Sciences* 2016;10(3):444-60.
54. Oluoch-Aridi J, Wafula F, Kokwaro G, et al. 'We just look at the well-being of the baby and not the money required': a qualitative study exploring experiences of quality of maternity care among women in Nairobi's informal settlements in Kenya. *BMJ Open* 2020;10(9):e036966.
55. Amooti-Kaguna B, Nuwaha F. Factors influencing choice of delivery sites in Rakai district of Uganda. *Social Science & Medicine* 2000;50(2):203-13.
56. Parkhurst JO, Penn-Kekana L, Blaauw D, et al. Health systems factors influencing maternal health services: a four-country comparison. *Health Policy* 2005;73(2):127-38.
57. Escamilla V, Calhoun L, Winston J, et al. The role of distance and quality on facility selection for maternal and child health services in urban Kenya. *Journal of Urban Health* 2018;95(1):1-12.
58. Fleming LC, Ansumana R, Bockarie A, et al. Inpatient healthcare provider bypassing by women and their children in urban Bo, Sierra Leone. *The Pan African medical journal* 2016;23:146. doi: 10.11604/pamj.2016.23.146.8706
59. Pyone T, Smith H, van dB. Implementation of the free maternity services policy and its implications for health system governance in Kenya. *BMJ Global Health* 2017;2(4):e000249. doi: 10.1136/bmjgh-2016-000249
60. Lang'at E, Mwanri L. Healthcare service providers' and facility administrators' perspectives of the free maternal healthcare services policy in Malindi District, Kenya: a qualitative study. *Reproductive Health* 2015;12:59. doi: 10.1186/s12978-015-0048-1
61. Njuguna J, Kamau N, Muruka C. Impact of free delivery policy on utilization of maternal health services in county referral hospitals in Kenya. *BMC Health Services Research* 2017;17(1):429. doi: 10.1186/s12913-017-2376-z
62. Miseda MH, Were SO, Murianki CA, et al. The implication of the shortage of health workforce specialist on universal health coverage in Kenya. *Human Resources for Health* 2017;15(1):80.
63. Tawfik DS, Scheid A, Profit J, et al. Evidence relating health care provider burnout and quality of care: a systematic review and meta-analysis. *Annals of Internal Medicine* 2019;171(8):55567.

For peer review only

64. Salyers MP, Bonfils KA, Luther L, et al. The relationship between professional burnout and quality and safety in healthcare: a meta-analysis. *Journal of General Internal Medicine* 2017;32(4):475-82.

65. Franco LM, Bennett S, Kanfer R. Health sector reform and public sector health worker motivation: a conceptual framework. *Social Science & Medicine* 2002;54(8):1255-66.

66. Herzberg F, Mausner B, Snyderman B. The Motivation to Work. 2 ed. New York: John Wiley & Sons 1959.

67. Ajayi AI, Akpan W. Maternal health care services utilisation in the context of 'Abiye' (safe motherhood) programme in Ondo State, Nigeria. *BMC Public Health* 2020;20(1):362. doi: 10.1186/s12889-020-08512-z [published Online First: 2020/03/21]

68. Ajayi AI. "I am alive; my baby is alive": understanding reasons for satisfaction and dissatisfaction with maternal health care services in the context of user fee removal policy in Nigeria. *PLoS One* 2019;14(12) doi: 10.1371/journal.pone.0227010 [published Online First: 2019/12/24]

69. Oyugi B, Kioko U, Kaboro SM, et al. A facility-based study of women'satisfaction and perceived quality of reproductive and maternal health services in the Kenya output-based approach voucher program. *BMC pregnancy and childbirth* 2018;18(1):310.

70. Umar N, Quaife M, Exley J, et al. Toward improving respectful maternity care: a discrete choice experiment with rural women in northeast Nigeria. *BMJ Glob Health* 2020;5(3):e002135. doi: 10.1136/bmjgh-2019-002135 [published Online First: 2020/03/24]

71. Audo M, Ferguson A, Njoroge P. Quality of health care and its effects in the utilisation of maternal and child health services in Kenya. *East African Medical Journal* 2005;82(11):547.

72. Cohen J, Golub G, Kruk ME, et al. Do active patients seek higher quality prenatal care?: a panel data analysis from Nairobi, Kenya. *Preventive Medicine* 2016;92:74-81.

73. Perera S, Weerasinghe M. Bypassing primary care in Sri Lanka: a comparative study on reasons and satisfaction. *Vietnam Journal of Public Health* 2015;3(1):69-76.

74. Sidze EM, Fenenga C, Amendah DD, et al. Are free maternal healthcare services programs an impediment to quality care? an examination of the Kenyan experience. *African Population and Health Research Center* 2015;5(1):1-10.

75. Witter S, Arhinful DK, Kusi A, et al. The experience of Ghana in implementing a user fee exemption policy to provide free delivery care. *Reproductive Health Matters* 2007;15(30):61-71. doi: 10.1016/S0968-8080(07)30325-X

76. Ganle JK, Parker M, Fitzpatrick R, et al. A qualitative study of health system barriers to accessibility and utilization of maternal and newborn healthcare services in Ghana after user-fee abolition. *BMC pregnancy and childbirth* 2014;14(1):434-62. doi: 10.1186/s12884014-0425-8

77. Ridde V, Queuille L, Kafando Y, et al. Transversal analysis of public policies on user fees exemptions in six West African countries. *BMC Health Services Research* 2012;12:409. doi: 10.1186/1472-6963-12-409

78. Ridde V, Diarra A. A process evaluation of user fees abolition for pregnant women and children under five years in two districts in Niger (West Africa). *BMC Health Services Research* 2009;9:1-16. doi: 10.1186/1472-6963-9-89

79. Bohren MA, Vogel JP, Hunter EC, et al. The mistreatment of women during childbirth in health facilities globally: a mixed-methods systematic review. *PLoS Medicine* 2015;12(6):e1001847.

80. Dalinjong PA, Wang AY, Homer CS. Are health facilities well equipped to provide basic quality childbirth services under the free maternal health policy? findings from rural Northern Ghana. *BMC Health Services Research* 2018;18(1):959.

For peer review only - http://bmjopen.bmj.com/site/about/guidelines.xhtml

81. Okoror CEM, Enabudoso EJ, Okoroh MI. Women's perception and satisfaction with the quality of antenatal care services in mission hospitals in Benin City, Nigeria. *Pyramid Journal of Medicine* 2020;3(1) doi: 10.4081/pjm.2020.82
82. Tesfaye R, Worku A, Godana W, et al. Client satisfaction with delivery care service and associated factors in the public health facilities of Gamo Gofa zone, Southwest Ethiopia: in a resource limited setting. *Obstetrics and Gynecology International* 2016;2016:1-7.
83. Masaba BB, Mmusi-Phetoe RM. Free maternal health care policy in Kenya: level of utilization and barriers. *International Journal of Africa Nursing Sciences* 2020;13:100234.
84. Chesumei EJ. Factors associated with uptake of free maternity services at Baringo County Referral Hospital. Jomo Kenyatta University of Agriculture and Technology, 2019.
85. Witter S, Adjei S, Armar-Klemesu M, et al. Providing free maternal health care: ten lessons from an evaluation of the national delivery exemption policy in Ghana. *Global Health Action* 2009;2(1):1881. doi: 10.3402/gha.v2i0.1881
86. Asule BM, Kwena A, Wambui T. Effects of the free maternity care program on utilization of services at a county referral hospital in Kenya. *Kenyan Journal of Nursing & Midwifery* 2017;1(2)
87. Khumalo N, Rwakaikara E. Patient satisfaction with peri-partum care at Bertha Gxowa district hospital, South Africa. *African Journal of Primary Health Care & Family Medicine* 2020;12(1):a2409.

For peer review only

Appendix 1: Maternal healthcare access characteristics

Variable		Total Frequency (%) n=550	Hospital A n (%) n=42	Hospital B n (%) n=170	Hospital C n (%) n=338	p-value
Facility visited during pregnancy	Yes	545 (99.09)	42 (100)	169 (99.41)	334 (98.82)	0.650
	No	5 (0.91)	0	1 (0.59)	4 (1.18)	
Type of facility visited	Public facility	506 (92.00)	38 (90.48)	149 (87.65)	319 (94.38)	**P<0.001***
	Private facility	28 (5.09)	1 (2.38)	17 (10.00)	10 (2.96)	
	Faith based organization (Mission)	7 (1.27)	-	3 (1.76)	4 (1.18)	
	Other	9 (1.64)	3 (7.14)	1 (0.59)	5 (1.48)	
Time taken to reach hospital	Below 30 minutes	137 (24.91)	12 (28.57)	45 (26.47)	80 (23.67)	0.309
	30 minutes-1 hour	264 (48.00)	19 (45.24)	87 (51.18)	158 (46.75)	
	1 hour-2 hours	121 (22.00)	7 (16.67)	35 (20.59)	79 (23.37)	
	More than 2 hours	20 (3.64)	4 (9.52)	2 (1.18)	14 (4.14)	
	Don't know	8 (1.45)	-	1(0.59)	7 (1.96)	
Perception of the time take to reach the hospital	Very short	60 (10.91)	6 (14.29)	25 (14.71)	29 (8.58)	0.340
	Short	249 (45.27)	18 (42.86)	73 (42.94)	158 (46.75)	
	Normal	99 (18.00)	12 (28.57)	32 (18.82)	55 (16.27)	
	Long	107 (19.45)	5 (11.90)	29 (17.06)	73 (21.60)	
	Very long	32 (5.82)	1 (2.38)	11 (6.47)	20 (5.92)	
	Don't know	3 (0.54)	-	-	3 (0.89)	

For peer review only

Perception about distance to the facility	Very near	69 (12.55)	16 (38.10)	22 (12.94)	31 (9.17)	**P<0.001**
	Normal	339 (61.64)	17 (40.48)	107 (62.94)	215 (63.61)	
	Far	110 (20.00)	8 (19.05)	33 (19.41)	69 (20.41)	
	Very far	28 (5.09)	-	8 (4.71)	20 (5.92)	
	Don't know	4 (0.73)	1 (2.38)	-	3 (0.89)	
Means of transport to the facility	Walking	27 (4.91)	7 (16.67)	14 (8.24)	6 (1.78)	**P<0.001**
	Bi/Motorcycle	60 (10.91)	1 (2.38)	20 (11.76)	39 (11.54)	
	Public transport (matatu/tuk tuk)	224 (40.73)	8 (19.05)	55 (32.35)	161 (47.63)	
	Private car/taxi	211 (38.36)	24 (57.14)	78 (45.88)	109 (32.25)	
	Ambulance	22 (4.00)	-	1 (0.59)	21 (6.21)	
	Combined modes	6 (1.09)	2 (4.76)	2 (1.18)	2 (0.59)	
Does opening hour suit your time?	Yes	431 (78.36)	41 (97.62)	152 (89.41)	238 (70.41)	**P<0.001**
	No	9 (1.64)	1 (2.38)	1 (0.59)	7 (2.07)	
	Don't know	76 (13.82)	-	15 (8.82)	61 (18.05)	
	N/A	34 (6.18)	-	2 (1.18)	32 (9.47)	
Waiting time at the facility	Very short	80 (14.55)	12 (28.57)	26 (15.29)	42 (12.43)	**P<0.001**
	Short	237 (43.09)	16 (38.10)	72 (42.35)	149 (44.08)	
	Normal	70 (12.73)	11 (26.19)	28 (16.47)	31 (9.17)	
	Long	80 (14.55)	1 (2.38)	22 (12.94)	57 (16.86)	
	Very long	43 (7.82)	2 (4.76)	22 (12.94)	19 (5.62)	
	N/A	40 (7.27)	-	-	40 (11.83)	
Hospital have a	Yes	422 (76.73)	40 (95.24)	134 (78.82)	248 (73.37)	**0.005**

For peer review only

proper waiting area	No	85 (15.45)	1 (2.38)	29 (17.06)	55 (16.27)
	Don’t know	28 (5.09)	1 (2.38)	7 (4.12)	20 (5.92)
	N/A	15(2.73)	-	-	15 (4.44)

Note: Chi square test of proportion was used to test difference in overall proportions of maternal health access characteristics.
*There is a statistical difference in the type of facilities that the mothers visited (majority visited public facilities).
Bold means **p-value <0.05**

For peer review only

Appendix 2: Referral characteristics

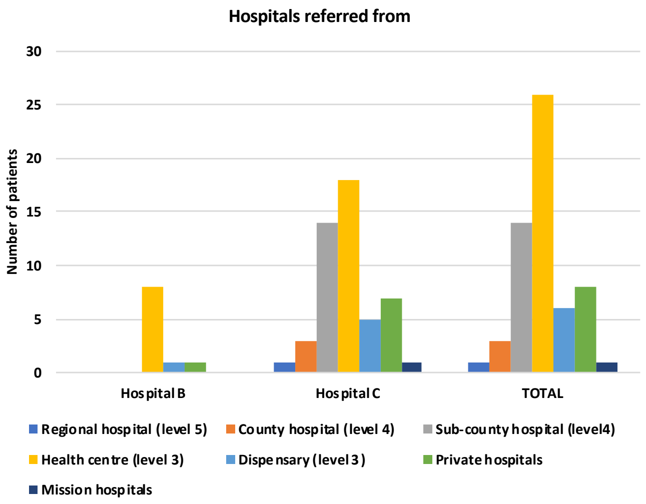

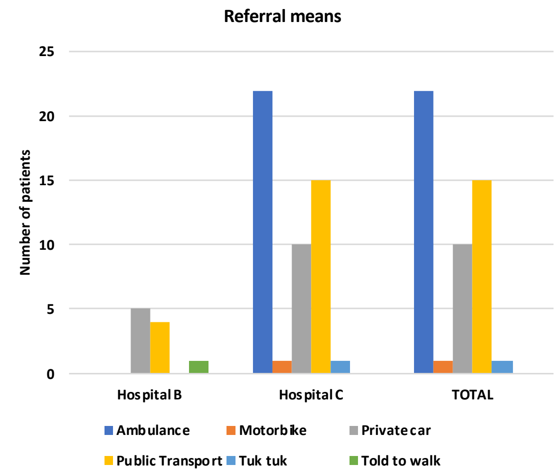

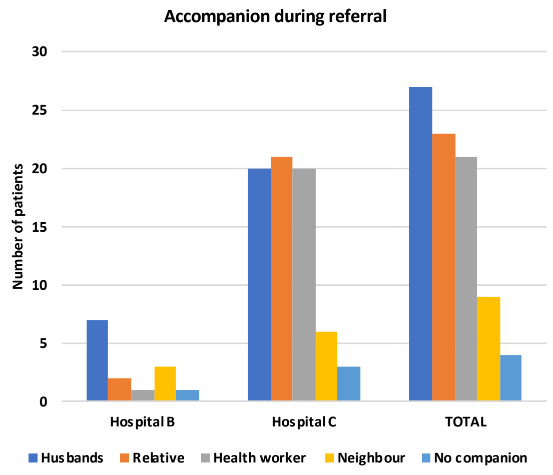

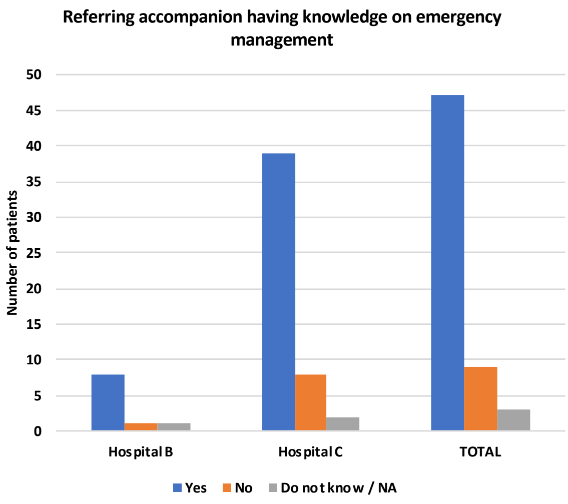

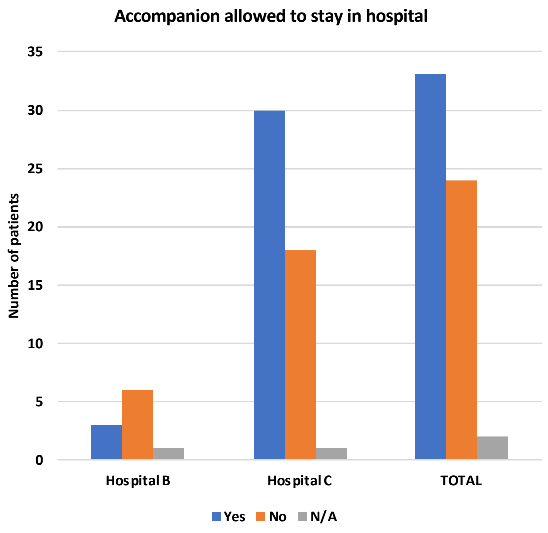

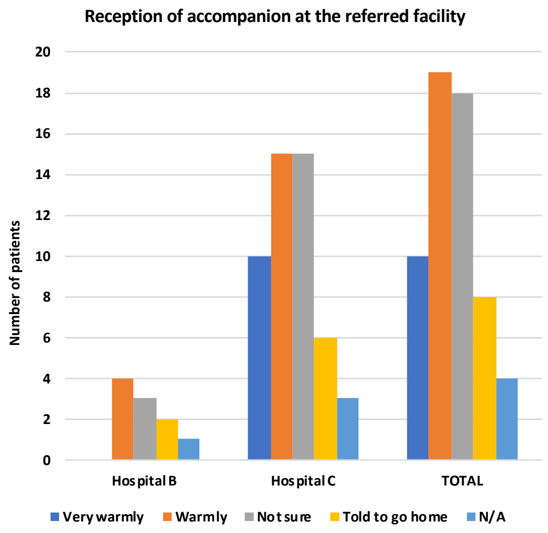

Appendix 3: Reasons for referral

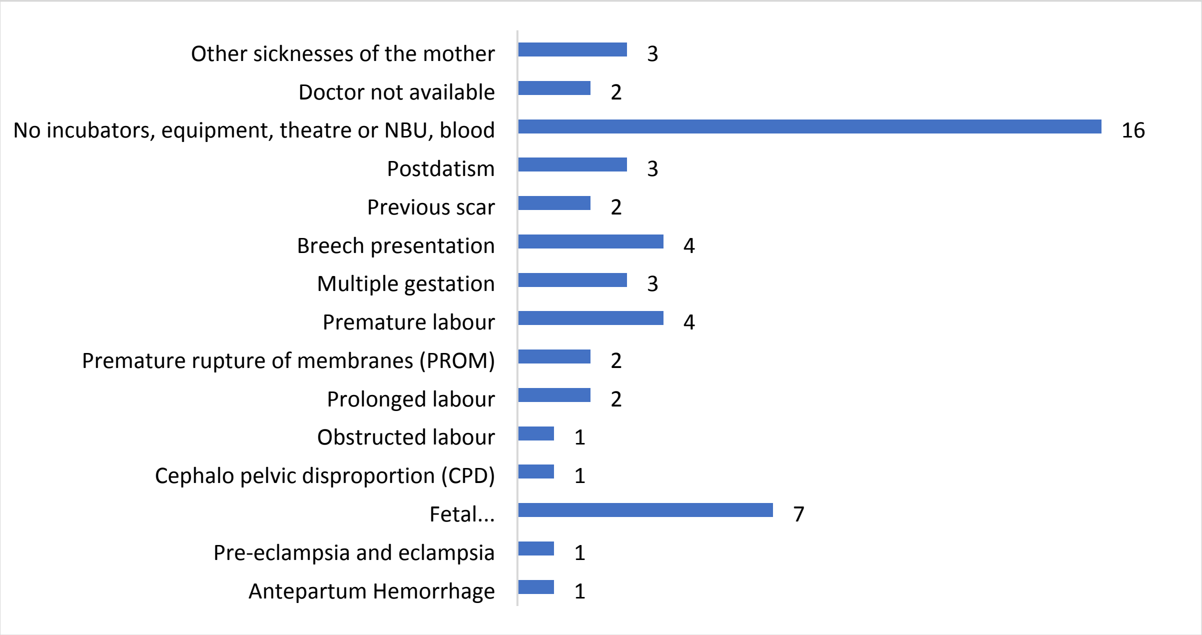

Appendix 4: Perception of quality of maternal care from the mothers exit interviews

Health Facility	CD	D	NS	A	CA
Number of staff adequate	0.73%	19.09%	3.82%	57.82%	18.55%
Staff well suited to treat women	0.36%	1.82%	1.27%	70.73%	25.82%
Waiting and examination rooms adequate	5.82%	38.73%	3.82%	40.55%	11.05%
Provision of clean drinking water adequate	3.82%	38.36%	11.46%	34.00%	12.18%
Hand washing facilities adequate	1.45%	6.00%	0.73%	70.55%	21.27%
Bathing facilities adequate	3.82%	24.36%	4.36%	52.91%	14.55%
Toilet facilities adequate	2.91%	24.00%	1.64%	55.45%	16.00%
Overal facility environment very clean	0.91%	3.64%	2.00%	70.00%	23.45%
Well suited equipment for detecting women's problems	0.91%	3.45%	4.73%	69.82%	21.09%
Distance from home very far	8.55%	59.09%	2.18%	23.82%	6.36%
**Healthcare delivery**	**CD**	**D**	**NS**	**A**	**CA**
Staff examine pregnant and post partum women well	0.91%	2.00%	1.82%	72.00%	23.27%
Staff very capable of finding out what is wrong with patients	0.73%	1.64%	2.18%	71.09%	24.36%
Staff prescribe drugs that are needed	0.00%	2.91%	37.45%	42.91%	16.73%
Drugs supplied by health facility are good	0.36%	1.45%	39.82%	42.55%	15.82%
Patients can obtain drugs from health facility easily	1.45%	5.64%	25.64%	52.00%	15.27%
Facility provided privacy very much during VE and delivery	3.82%	9.64%	6.73%	63.82%	16.00%
Felt very much of necessary procedure during ANC and delivery	3.83%	8.38%	3.10%	65.39%	19.31%
Adequate Information on danger signs of delivery and postpartum	1.45%	0.24%	3.27%	49.45%	21.82%
**Interpersonal Aspects**	**CD**	**D**	**NS**	**A**	**CA**
Staff very open with the patients	0.18%	3.83%	1.64%	68.61%	25.73%
Staff very compasionate towards the patients	1.27%	5.45%	2.73%	66.00%	24.58%
Staff are respectful towards the patients	0.18%	2.73%	1.64%	69.64%	25.82%
Time staff devete to the patients is adequate	0.36%	4.36%	1.09%	67.27%	26.91%

For peer review only

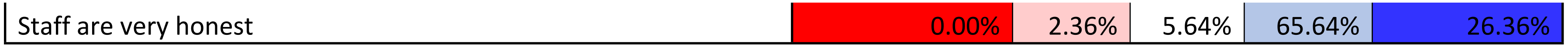

For peer review only

BMJ Open

Examining the quality of care across the continuum of maternal care (antenatal, perinatal, and postnatal care) under the expanded free maternity policy (Linda Mama Policy) in Kenya: a mixed-methods study

Journal:	*BMJ Open*
Manuscript ID	bmjopen-2023-082011.R1
Article Type:	Original research
Date Submitted by the Author:	05-Mar-2024

Complete List of Authors:	Oyugi, Boniface; M and E Advisory Group, P.O. Box 6523 - 00200, Western Heights, The Mint Nairobi; University of Kent, Centre for Health Services Studies<br>Audi-Poquillon, Zilper; The London School of Economics and Political Science, Department of Health Policy<br>Kendall, Sally; University of Kent, Centre for Health Services Studies<br>Peckham, Stephen; University of Kent, Centre for Health Services Studies

**Primary Subject Heading**:	Health services research
Secondary Subject Heading:	Evidence based practice, Health economics, Health policy, Health services research, Global health
Keywords:	Health economics < HEALTH SERVICES ADMINISTRATION & MANAGEMENT, Health policy < HEALTH SERVICES ADMINISTRATION & MANAGEMENT, Maternal medicine < OBSTETRICS, Quality in health care < HEALTH SERVICES ADMINISTRATION & MANAGEMENT, Community child health < PAEDIATRICS, HEALTH ECONOMICS

SCHOLARONE™ Manuscripts

BMJ

I, the Submitting Author has the right to grant and does grant on behalf of all authors of the Work (as defined in the below author licence), an exclusive licence and/or a non-exclusive licence for contributions from authors who are: i) UK Crown employees; ii) where BMJ has agreed a CC-BY licence shall apply, and/or iii) in accordance with the terms applicable for US Federal Government officers or employees acting as part of their official duties; on a worldwide, perpetual, irrevocable, royalty-free basis to BMJ Publishing Group Ltd ("BMJ") its licensees and where the relevant Journal is co-owned by BMJ to the co-owners of the Journal, to publish the Work in this journal and any other BMJ products and to exploit all rights, as set out in our licence.

The Submitting Author accepts and understands that any supply made under these terms is made by BMJ to the Submitting Author unless you are acting as an employee on behalf of your employer or a postgraduate student of an affiliated institution which is paying any applicable article publishing charge ("APC") for Open Access articles. Where the Submitting Author wishes to make the Work available on an Open Access basis (and intends to pay the relevant APC), the terms of reuse of such Open Access shall be governed by a Creative Commons licence – details of these licences and which Creative Commons licence will apply to this Work are set out in our licence referred to above.

Other than as permitted in any relevant BMJ Author's Self Archiving Policies, I confirm this Work has not been accepted for publication elsewhere, is not being considered for publication elsewhere and does not duplicate material already published. I confirm all authors consent to publication of this Work and authorise the granting of this licence.

For peer review only

Examining the quality of care across the continuum of maternal care (antenatal, perinatal, and postnatal care) under the expanded free maternity policy (Linda Mama Policy) in Kenya: a mixed-methods study

Authors

Boniface Oyugi[1,2], **Zilper Audi-Poquillon**[3*], **Sally Kendall**[2], **Stephen Peckham**[2]

1. M and E Advisory Group, P.O. Box 6523 - 00200, Western Heights, The Mint Nairobi, Kenya.
2. Centre for Health Services Studies (CHSS), University of Kent, George Allen Wing, Canterbury CT2 7NF
3. Department of Health Policy, London School of Economics and Political Science

***Corresponding Author: Zilper Audi-Poquillo (ZAP)** – z.a.audi-poquillon@lse.ac.uk

Email addresses:
BO: b.oyugi@kent.ac.uk
SK: S.Kendall-608@kent.ac.uk
SP: S.Peckham@kent.ac.uk

ORCID IDs
Boniface Oyugi: https://orcid.org/0000-0002-9550-9138
Zilper Audi-Poquillon: https://orcid.org/0009-0002-6901-7348
Sally Kendall: https://orcid.org/0000-0002-2507-0350
Stephen Peckham: https://orcid.org/0000-0002-7002-2614

Qualification
BO: PhD (Dr)
ZAP: MSc (Ms)
SK: PhD (Professor)
SP: (Professor)

For peer review only

ABSTRACT

Background

Kenya still faces the challenge of mothers and neonates dying from preventable pregnancy-related complications. The free maternity policy (FMP), implemented in 2013 and expanded in 2017 (Linda Mama Policy (LMP)), sought to address the challenge. This study examines the quality of care (QoC) across the continuum of maternal care under the LMP in Kenya.

Methods

We conducted a convergent parallel mixed-methods study across multiple levels of the Kenyan health system, involving key informant interviews (KIIs) with national stakeholders (n=15), in-depth interviews (IDIs) with County officials and healthcare workers (HCWs) (n=21), exit interview survey with mothers (n=553) who utilised the LMP delivery services, and focus group discussions (FGDs) (n=9) with mothers who returned for postnatal visits (6, 10, and 14 weeks). Quantitative data was analysed descriptively, while qualitative data was analysed thematically. All the data were triangulated at the analysis and discussion stage using a framework approach guided by the QoC for Maternal and Newborns.

Results

The results showed that the expanded FMP enhanced maternal care access: geographical, financial, and service utilisation. However, the facilities and HCWs bore the brunt of the increased workload and burnout. There was a longer waiting time for the initial visit by the pregnant women because of the enhanced antenatal care (ANC) package of the LMP. The availability and standards of equipment, supplies, and infrastructure still posed challenges. Nurses were multitasking and motivated despite the human resources challenge. Mothers were happy to have received care information; however, there were challenges regarding respect and dignity they received (inadequate food, over-crowding, bed-sharing and lack of privacy), and they experienced physical, verbal, and emotional abuse and a lack of attention/care.

Conclusions

There is a need to address the negative aspects of QoC while strengthening the positives to achieve the UHC goals through better quality service for every woman.

Keywords: quality of care, maternal and childcare, maternal care, Linda Mama, free maternity policy, Kenya

For peer review only

STRENGTH AND LIMITATIONS OF THIS STUDY

- This is the first study that has explored the optimal quality of care (QoC) across the continuum of maternal care (antenatal, perinatal, and postnatal care) under the expanded free maternity policy in Kenya using the QoC for Maternal and Newborn – a monitoring framework for network countries.
- The use of a mixed methods approach in this study permitted for complementarity, convergence and triangulation of the qualitative and quantitative data to deepen the description and analysis of the policy, hence attenuating the weaknesses of the singular methods.
- While the results may not be generalisable beyond the study county (area) because of the heterogeneity of the counties, this study identifies significant contextual factors that may have influenced the patterns of implementation and the findings which are transferable (enhanced transferability) to other 47 counties in the counties and can be used to interpret the implications of the results in other settings.
- There could be many other unidentified QoC elements from this study, particularly other county-specific issues, but the findings could be considered the first step in exploring and compiling the existing knowledge about the global situation.
- This study could be particularly informative for policymakers as a guide to effective evidence-based interventions that can be adopted to strengthen the implementation of the FMP in the country.

For peer review only

INTRODUCTION

There are nearly 287,000 maternal deaths due to preventable pregnancy and childbirth-related complications happening globally (translating to almost 800 maternal deaths every day or one every two minutes) (1). Low-and-middle-income countries (LMIC) and low-income countries (LIC), especially those in sub-Saharan Africa (SSA), such as Kenya, are the most affected because of barriers to accessing maternal services (such as low quality of care (QoC), poor socio-economic conditions, poor infrastructure, and lack of well-trained healthcare professionals) (2-4). While Kenya's maternal and child health status has significantly improved in the last decade, the current maternal mortality ratio (MMR) of 530 deaths per 100,00 live births is significantly higher than the world average of 223 maternal deaths per 100,000 live births,(1) as is the neonatal mortality rate (NMR) of 21 deaths per 1,000 live births which is higher than the world average of 18 deaths per 1,000 (5, 6). Approximately 7,300 women still die every year making up 15% of all deaths among women of reproductive age, with both mothers and neonates dying from preventable pregnancy-related complications (7). One in 76 women in Kenya is at risk of dying from pregnancy complications (8).

As such, reducing and eliminating pregnancy-related mortality, ending preventable newborn and child mortality, and achieving Universal Health Coverage (UHC) remain crucial targets and priorities for realising the Sustainable Development Goals (SDGs) in Kenya. Various reforms in the health sector in Kenya have sought to achieve the above SDG targets by reducing catastrophic expenditure on maternity care and improving the quality of healthcare service delivery (9-13). One such reform was initiated in Kenya in June 2013, when the government launched a user fee waiver for maternity and primary health care (PHC) services (9). However, its implementation faced challenges of poor service delivery due to inadequate preparation before the implementation and a lack of adequate systems to verify the QoC provided and the reimbursement claims from the hospitals to the government (14).

Subsequently, to overcome these challenges, the country transitioned to a new expanded free maternity policy (FMP) in 2017 to provide access to maternal services to all pregnant women in an expanded network of providers including, private, faith-based, and all level 3–6 public institutions (15). The expanded FMP was called Linda Mama (LM) (Swahili for "*caring for the mother*"), and was managed through the National Hospital Insurance Fund (NHIF) to overcome challenges from the previous policy by enhancing administrative efficiency, ameliorating the reimbursement logistical challenges, creating a longer-term financing sustainability, and easing legal hurdles (16). Besides, it aimed to improve access to quality maternal and child services and reduce inequalities, thereby advancing the country's agenda of UHC (15, 17). The benefits package of the expanded policy captured both inpatient and outpatient services (including more antenatal services, delivery, postnatal care, and referrals of emergencies of pregnancy-related conditions and complications) for the mother and the newborn up to a year (18, 19).

Being part of the reform linked to the UHC agenda, there were three facets targeted for improvements: population, services and direct costs,(20) envisaging that every person would have access to the entire range of quality health services and care they needed, whenever and wherever they needed them, without financial hardship (21, 22). The LM policy was mainly implemented to achieve the three facets. However, following the implementation of the two free maternity policies, researchers have focussed on understanding the facets: population and cost, through studies focused on the policy's immediate and trend effect,(23) its impacts on mortality and utilisation of services,(24-

For peer review only - http://bmjopen.bmj.com/site/about/guidelines.xhtml

27) out of pocket expenditure,(28) policy formulation and implementation elements,(15-17) and the cost-benefit analysis (27). While there has been an attempt to look at the services, the quality of services and care aspects from both policies has not been conclusive and a gap remains. For instance, one study evaluated the satisfaction with the delivery services under FMP (29). It showed that the mothers who benefited from the services were satisfied with different components such as communication by the healthcare workers (HCWs), staff availability in the wards and delivery rooms, and supplies availability, but were also unsatisfied with cleanliness, consultation time, and privacy in the wards. Another study evaluating the utilisation of the free maternity services implemented in 2013 among women living in Kibera slums in Nairobi showed that mothers positively perceived the distance to the facility and shorter waiting time, in addition to patients facing bad providers' attitudes (30). Yet, another study that evaluated disrespectful maternal care under the policy in Kisii and Kilifi counties showed that mothers experienced disrespectful maternal care throughout the maternity process, and it appeared even more significant among women who were poor, young, or had children with disabilities (31). All three studies on quality have focused on one aspect of quality: the outcome (from the patient perspective), leaving out other quality dimensions that researchers (32, 33) have discussed: structure, process, and outcome.

Therefore, the quality-of-service facet is yet to be fully explored. One study evaluated the characteristics associated with the QoC of the initial assessment for pregnant mothers, intrapartum, and postpartum and newborn care (continuum of care) not under the FMP but in the country context using service provision data and the finding was that a sustained focus on the QoC along the maternity care continuum was imperative for the mothers and their newborns and policymakers (in distributing resources to improve the areas of the continuum (34). Increasing service coverage alone is unlikely to produce better health outcomes without attention to the quality of care provided. The LM policy seeks to be a high-quality health intervention that optimises maternal care in the Kenyan context by consistently delivering and giving care that enhances or maintains maternal and neonatal outcomes, and that is valued and trusted by everyone since it responds to a changing population's needs (35). Maternal care under LM policy envisages enhancing the degree to which maternal services received by clients increase the likelihood of desired health outcomes consistent with current professional knowledge and are effective, safe, people-centred, timely, equitable, integrated and efficient (36). Therefore, exploring the optimal quality of maternal care and outcomes from the LM policy would be imperative. This study examines the QoC across the continuum of maternal care (antenatal, perinatal, and postnatal care) under the LM Policy in Kenya.

METHODS

Study Design

We utilised the convergent mixed methods design, specifically the parallel-database variant in this study (37) using qualitative and quantitative data that were collected and analysed in tandem and then compared and combined to better understand the QoC across the continuum of maternal care (antenatal, perinatal, and postnatal care) under the free maternity policy in Kenya.

Framework for analysis

As quality cannot be measured by itself,(38) in this study, we conceptualised quality from the Donabedian perspective, broadly classifying quality as structure, process, and outcome dimensions,(32, 33) which can be identified, measured, and attributed to healthcare. Akachi and Kruk

For peer review only - http://bmjopen.bmj.com/site/about/guidelines.xhtml

(39) provide more details on measuring changes in the QoC and bring attention to including user experience as a measure of outcomes in the quality assessment. With these two refined aspects, we broadly defined the structure indicators as pointers which are inputs to or characteristics of health; process indicators as gauges to either appropriate or inappropriate care in a targeted population which are 'consistent with current professional knowledge'; and outcome indicators as the measures of both improved or deteriorated health and attributed to medical care (38, 39). (See, Figure 1). Data collection methods and tools were designed to collect and examine all aspects of QoC across the continuum of maternal care (antenatal, perinatal, and postnatal care) under the free maternity policy in Kenya. Broadly, the analysis converges all the concepts using the QoC for Maternal and Newborn – a monitoring framework for network countries,(40) which draws concepts from the earlier framework as proposed by the World Health Organisation (41).

[INSERT FIGURE 1 HERE]

Study setting

The study was conducted across multiple levels within the Kenyan health system. The Kenyan health system is pluralistic in the provision and financing of services and is organised into six levels of care. Level 1 forms the community units overseen by community health workers (CHWs) whose role is providing promotive services (health education, treating minor ailments, and identifying cases that require referral to health facilities),(42) and both level 2 (dispensaries) and level 3 (health centres) provide primary healthcare services in addition to coordinating the community in their areas of jurisdiction. Level 4 and 5 offer curative services as county secondary referral facilities, with some being training centres, while level 6 are semi-autonomous tertiary facilities offering specialised care and serving as training institutions.

At the national level, we included the Ministry of Health, the NHIF, and development partner agencies involved in the expanded free maternity policy. At the County level, this study was conducted in Kiambu County in Kenya. While this study is part of a larger study, Kiambu County was purposefully chosen because of its sociodemographic characteristics, health indicators, and population size (43-45). It is the second-most populous county in Kenya after Nairobi City County, with a population of 2,417,735: 49.1% male and 50.59% female,(43) 26.9% of the population in Kiambu are female of reproductive age (15-49 Years),(44) 89.2% of births in the county happen in a health facility, 98.2% of births provided by a skilled provider, 67% of women aged 15-49 who had a live birth had 4+ antenatal visits, and 89% of women aged 15-49 had a postnatal check during the first two days after birth (5). While these statistics are slightly higher than the national average, they have not translated to quality care. Research has shown that primary care facilities with a low delivery volume have very low-quality delivery care, indicating crucial deficiencies in infrastructure and staffing, routine and emergency care practices, and referral systems (46). A majority of the facilities providing care in Kiambu are primary care facilities (70 tier 2 - dispensaries and tier 3 - health centres) with low volumes compared to the secondary facilities (13 tier 4 - hospitals and 1 tier 5 - inter-county facility) with a high volume (47). With the secondary care facilities receiving a higher population for care from the neighbouring counties and the locals,(48) they are bound to be stretched beyond the limit, hence the potential for challenges in the quality of care. Kiambu faces challenges with the interventions to address maternal health, such as referral systems that work, family planning, access to safe abortion services, availability of skilled health workers, and accessible health facilities (49-51) despite being cosmopolitan. With the

For peer review only - http://bmjopen.bmj.com/site/about/guidelines.xhtml

majority of the population in Kiambu being urban,(43) which is facing overcrowding, there is a potential for the urban averages of maternal mortality to become either closer or worse than rural averages. Further, the researchers purposively chose Kiambu County because they were constrained with resources to conduct the larger study (52) because of the logistic feasibility of data collection (due to its proximity to Nairobi County - where the data collectors were based - and the cost implication accompanying data collection). But also, the county has been shown to poses higher trends in maternal mortality of the counties around Nairobi from Central Region (53).

We purposefully selected three study facilities: a level 3 (considered a low volume – few numbers of clients), a level 4 (medium volume), and a level 5 (high volume). The facilities were chosen in consultation with the county team to provide nuanced, unique sub-counties dynamics given their richness in information and characteristics. (See Table 1).

Study population, sampling, and data collection

The study population used in this study were in four categories, as summarised in Table 1. We collected data between November 2018 and September 2019 through exit interviews (EIs), focus group discussions (FGDs), in-depth interviews (IDIs), and key informant interviews (KIIs).

The first group was staff from the Ministry of Health, NHIF, and development partners, who were purposefully selected based on their level of involvement in the expanded free maternity policy. These respondents participated in KIIs with one researcher (BO), which were done in English, using KII guides developed to capture the experience of the formulation and implementation of the expanded FMP. All the KIIs (n=15) were conducted in Nairobi and were audiotaped following participants' consent using audio recorders. Each KII lasted between 45-60 minutes.

The second category included purposively selected respondents with knowledge of and experience in the implementation of the expanded FMP at the county (meso) level (including county and sub-county level officials from the County Department of Health); and the facility (micro) level (including facility in-charge, HCWs in charge of /offering maternal care/services, and other cadres of hospital workers) (Table 1). These respondents participated in IDI with one researcher (BO). The IDIs (n=21) were conducted in English using two semi-structured guides (each for the county and health facility participants) developed to capture the experience of implementing the expanded FMP. The construct validity of the two semi-structured guides was tested in the non-participating facility to check for ambiguity and flow of the questions. All the IDIs (save for one conducted at the place of convenience for the participant) were conducted at the participants' places of work and were audiotaped using audio recorders after obtaining their consent. Each IDI lasted between 30-60 minutes. The KIIs and IDIs were stopped at the point where meaning saturation (where no new information, further dimensions, nuances, or insights were forthcoming) was attained (54). At this point, we noted that we had fully understood the issue under discussion.

The third group comprised of EIs with mothers who had delivered in the three hospitals and were discharged home. The sample size of the mothers was estimated at 553 using the formula proposed by Gorstein et al. (55). A detailed discussion of the sample criteria and dynamics across the three selected facilities has been published elsewhere (52). Four trained data collectors, supervised by one researcher (BO), conducted the EIs with the women. The design of the EI utilised a structured

For peer review only - http://bmjopen.bmj.com/site/about/guidelines.xhtml

questionnaire, adapted from Dalinjong et al.,(56) to elucidate the sociodemographic information of the women, health and related services received at the facility (perception of the quality of maternal care that the mothers received during delivery and antenatal care (ANC) care, experiences with the FM policy). The conduct of the EIs ensured that one researcher (BO) introduced the data collectors to the administration and the maternity department heads of the three facilities; then, each morning of the interview, they identified the mothers who had been discharged (using bed numbers) and were waiting to return home. With the number of mothers identified per day, we generated a random sample using Stat Trek's Random number generator,(57) which was used to identify mothers for the EI. The mothers were then invited to participate in the study, and interviews were conducted until we reached the intended sample size. We took each mother through the information sheet, and only when they were comfortable participating did we give them the consent forms. One mother declined to participate (and we eliminated two entries at the analysis stage for lacking complete information).

The final category included FGD with nine groups of mothers (ranging from 5-12 mothers) purposively selected based on a common interest: mothers who had had a skilled delivery in a hospital setting and had come to the study sites for the 6-, 10-, or 14- week postnatal visits. One researcher (BO) conducted all 9 FGDs in Swahili (given the different levels of knowledge of the participants) using an FGD guide developed in reference to the gaps that had arisen from the EIs. The mothers in the FGD were recruited from the child welfare clinic of the three facilities when they brought their children for routine vaccination. The FGDs in each facility were organised with the help of a nurse from the maternity departments. We engaged the mothers as the children received their vaccinations and asked if they would participate in the study. All the FGDs were conducted in a pre-booked room at the facilities and were audiotaped following participants' consent using audio recorders. Each FGD lasted between 45-90 minutes.

Table 1: Hospital characteristics and study population

	**Level 3 Hospital (Hospital A)**	**Level 4 Hospital (Hospital B)**	**Level 5 Hospital (Hospital C)**
**Hospital characteristics**			
Bed and cots capacity[a]	10	46	289
Number of staff[b]	35	115	262
Estimated annual deliveries[c]	1,076	5,635	9,152
Estimated annual outpatient care[c]	88,829	156,108	281,379
Estimated annual inpatient care[c]	764	7,223	14,205
**Hospital participants in the study**			
EIs	42	170	338
FGDs	3	3	3
IDIs	7	5	6
Facility level managers	*1*	*3*	*2*
Department in charges	*1*	*1*	*1*
Nursing officers	*4*	*0*	*1*
Accounting/ clerical officers	*1*	*1*	*2*
**County participants (IDI)**			**3**
Senior level managers			1
Middle-level manager			2

For peer review only

**National participants (KIIs)**	**15**
Ministry of Health officials	5
NHIF officials	3
Development partners	7

Notes: *Estimates for annual delivery, outpatient care and inpatient care were for the financial year July 2018 – June 2019; The outpatient total is an aggregate of both new and revisits.*
EIs: *Exit Interviews;* ***FGDs:*** *Focus Group Discussions;* ***IDIs:*** *In-depth Interviews; and* ***KIIs:*** *Key Informant Interviews*

Source: [a]Kenya Master Health Facility List(58), [b]In-depth interview with health facility in-charges of the individual facilities; [c]Kenya Health Information System (KHIS) for aggregate reporting(59).

Data management and analysis

Quantitative data from the EI was manually entered from the structured questionnaire into the Excel software by one researcher (BO), cleaned, checked for completeness, and then exported to STATA 15 for coding and analysis. The sociodemographic characteristics and the elements of quality were analysed descriptively using proportions.

All recorded FGDs were translated from Swahili to English, while the IDIs were transcribed verbatim in English. All transcripts were compared against their respective audio files by BO for transcription and translation accuracy. All the validated transcripts were imported into NVivo 12 for coding guided by the topic areas of quality of maternal healthcare. We used a framework approach to analyse the data guided by the QoC for Maternal and Newborn – a monitoring framework for network countries (40). This approach included systematic sifting, sorting, coding, and charting data into key issues and themes (60). One researcher (BO) familiarised himself with the data through immersion and repeatedly read and reread the transcripts. He then developed codes deductively from the conceptual framework and applied the codes to interpret segments in the transcripts that were important. The study team members (SK and SP) reviewed and discussed the initial coding framework, and any discrepancies were appropriately reconciled. The final coding framework was applied by (BO) to the data and later charted the data to allow the emergence of themes through comparisons and interpretations.

To enhance the interpretive rigour, we ensured credibility (also referred to as internal validity) through the convergence of evidence of the two methods utilised and triangulation (investigator, theoretical, and methodological) of data at the interpretive stage (61).

Ethics consideration

This study was part of a larger study (52) whose ethical approval was obtained from the University of Kent, the SSPSSR Students Ethics Committee and the AMREF Scientific and Ethics Review Unit in Kenya (Ref: AMREF – ESRC P537/2018). Further, we received written permission to conduct the study from the county government, and all the hospitals. We obtained written and oral informed consent from the potential participants before starting the interviews. All the study participants were presented with information sheets on the conduct of the study, the researchers involved, the purpose of the study, the right to withdraw, and measures of confidentiality ensured before they gave their written informed consent. Participants were informed that data would be reported in an aggregated format, and anonymity would be ensured in storing and publishing the study's findings.

For peer review only - http://bmjopen.bmj.com/site/about/guidelines.xhtml

Patient and public involvement

Patients and/or the public were not involved in the design, or conduct, or reporting, or dissemination plans of this research. We intend to disseminate these research findings to the public through a summarised press article or brief, social media, and the websites of authors' institutions.

RESULTS

The results on the quality of maternal care in this study were presented using the WHO-proposed monitoring logic model from the perspective of the implementers and the users of the policy. Results are presented in four broad domains: access to care (equitable and timely), provision of care (safe and effective), management and organisation, and care experience. A summary of the results is presented in

Table *2*.

Table 2: Summary of the quality of maternal care results

Domain	Sub-domain	Positive result	Negative result
**Element 1: Access to maternal care services under the expanded FMP (equitable and timely)**	***Minimised access barriers (cultural, financial, geographic)***	The expanded FMP enhanced maternal care access elements (geographical, financial, or utilisation of services).	However, the facilities and HCWs were bearing the brunt of the burden of increased numbers of mothers seeking LM care (workload and burnout)
			There was an altered perception among women, leading to a preference for higher-level facilities.
	Additional maternal determinants of care and the timeliness of care	The distance to the hospital was perceived as normal (okay for the patients) and the preferred choice of transport to the facility was public transport	There was a longer waiting time for the initial visit by the pregnant women due to the enhanced ANC package of the expanded FMP.
		All the three hospitals had a proper waiting area.	
		There was a positive perception about the time to seek care and the waiting time.	
	Provider availability		There were problems of struggling to employ specialists and other HCWs staffing challenges.
**Element 2: Provision of care (safe and effective)**	***Functional referral system***		Fewer women are being referred, but they have a better perception of services received during referral.
			A lack of equipment, theatre, NBU and blood in the facilities were the main reason for referrals.

For peer review only

	Safety	Because of the policy, the facilities were managing complications better	HCWs were reducing the time they allocate per mother.
**Element 3: Management and organisation**	***Availability of essential physical resources***	The policy has improved the availability and standards of equipment and supplies.	Despite progress, some infrastructure, commodities, and supplies are still a challenge to some facilities.
		The facilities had improved infrastructure due to LM.	
		Enhanced facility resources and facility characteristics.	
	Competent and motivated staff	Mothers have a strong positive perception of healthcare delivery characteristics by the HCWs.	There were some causes of demotivation and dissatisfaction among HCWs.
		Nurses are multitasking and handling many roles amidst the challenge of human resources.	
		HCWs are adequately motivated to work despite the challenges.	
		HCWs' source of motivation was more than just money.	
	Monitoring and continuous quality improvement	Nurses monitor the quality of care provided through partographing and charting labour progress, though they face challenges.	
**Element 4: Experience of care**	***Effective communication with the patients***	Mothers perceived and experienced the positive interpersonal qualities of the HCWs.	Inadequate preparation for birth by the HCWs.
		Mothers were happy to have received information about emergency/ procedures and training on breastfeeding, family planning, and baby care.	The lack of proper 11ducationn and communication on expectations.
	Respect and dignity		Food was perceived as inadequate in some hospitals.
			There was over-crowding and bed-sharing, leading to a lack of privacy (congestion), and a lack of essential equipment and supplies, altering the QoC.
	Emotional support		Women were experiencing physical, verbal, and emotional abuse.
			Some mothers experienced a lack of attention/care, negligence, and unhygienic practices from the HCWs and support staff.

Element 1: Access to maternal care services under the expanded FMP (equitable and timely)

For peer review only - http://bmjopen.bmj.com/site/about/guidelines.xhtml

Minimised access barriers (cultural, financial, geographic)

The expanded FMP enhanced maternal care access elements (geographical, financial, or utilisation of services). For instance, due to the policy, there was an increase in the utilisation of maternal services (delivery and ANC). Findings from Eis showed that most mothers across the study sites (99.09%, n=545) visited a hospital for maternal health services during their pregnancy (Appendix 1). Further, IDI showed that more mothers (than previously) were confident in seeking skilled services rather than remaining at home.

> *'…mothers who could not come, now they are coming. And there is also a change in the number of deliveries we used to have before and now'* – ***(R009, Nursing officer).***

Equally, the respondents noted that with the enhanced identification strategies for the mothers, the expanded FMP saw increased access to services among vulnerable populations such as street children, orphans, and adolescents. Besides, they averred that there was enhanced equity and financial access to the services by the women as those in the rural and urban areas received uniform services for free.

However, the facilities and HCWs were bearing the burden of increased numbers of mothers seeking LM care. As noted by most respondents, facilities were bearing the brunt of the increased number of mothers due to LM, which resulted in space shortages and increased workload. The workload was further exacerbated by the nature of work in the public facilities where the HCWs had no choice but to serve the mothers and meet the required utilisation targets. However, the facilities were working way beyond their abilities to manage the workload, and it resulted in HCWs experiencing some burnout:

> *'We work extra hours…you will find each care provider is serving more than they should, so the issue of burnout is also coming up'* – ***(R019, Facility Level Manager)***

There was an altered perception among women, leading to a preference for higher-level facilities. There was an increased workload in higher-level facilities caused by the mother's perception of there being specialist health care professionals that the lower-level dispensaries or community centres lack. As a result, the women believed that higher-level facilities had a higher chance of dealing with complications than the lower-level hospitals:

> *'…sometimes you ask them, "Why have you decided to come here?" "Because here, people who will attend to me are qualified."…But they say outside there, anybody can attend you.'* – ***(R014, Nursing officer).***

Additional maternal determinants of care and the timeliness of care

There was a positive perception about the time taken to seek care and the waiting time. A majority of the women visited a public facility (92%); and had a positive perception about the time taken to the facility and the distance to the hospital. Women who visited hospitals A (45.24%), B (51.18%), C (46.75%), and overall (48.00%)) noted that they took 30 minutes to 1 hour to seek delivery services and they perceived the time to be short (Appendix 1).

A majority (61.64%) perceived the distance to the hospital was normal (okay for the patients), and the preferred choice of transport to the facility was public transport (40.73%) (Appendix 1). Also, all three hospitals had a proper waiting area. While most of the women were happy with the time the facilities

For peer review only - http://bmjopen.bmj.com/site/about/guidelines.xhtml

were being opened and perceived the waiting time before being attended to as short (43.09%) (Appendix 1).

There was a longer waiting time for the initial visit by the pregnant women due to the enhanced ANC package of the expanded FMP. The initial ANC profile included blood tests (for haemoglobin levels, blood group, rhesus, serology), Screening for tuberculosis, HIV testing and counselling, urinalysis, preventive services (such as deworming, intermittent preventive treatment for malaria, iron and folate supplementation) and prevention of mother to child transmission. All these were done at the same laboratory as other patients in the hospitals; hence, they had to wait for longer to get results:

> *'…for the first visit [they] will report here at 8:00[am] and…get out of this place as late as 3:00[pm]…because when they come…if it's lab everybody is there, the people who are coming for outpatient services are queuing there [too]…the rebate for the first visit [ANC]…covers up a lot'* – ***(R002, Clerical Officer).***

Provider availability

There were problems of struggling to employ specialists and other HCWs staffing challenges. The facility in-charges noted that they had a challenge of hiring specialist nurses to take care of the growing numbers, which had been exacerbated by the lack of specialised units:

> *'…we could not set up a neonatal ward [for lack of] a neonatal nurse...[yet] we get so many babies, and with that influx, we could still get some babies...'* – ***(R020, Facility level manager)***

One in-Charge noted that while the facilities had installed an ultrasound machine to meet the needs of the pregnant mothers, there was a gap in trying to identify the person to operate it and sustainably pay the staff.

The staffing challenge, particularly in the lower-level facilities, was hard to deal with because of the rules of staffing where, despite the high number of mothers, the number of staff cannot go beyond a certain number:

> *'…I think it's not because of Linda Mama, I think it's because of how it has been, we have been a level 3, although they said they would add us people. But you see they cannot exceed the number of staff in a level 3. If it were a level 4, they would increase.'* – ***(R007, Department in-charge)***

Element 2: Provision of care (safe and effective)

Functional referral system

Fewer women are being referred, but they have a better perception of services received during referral. While referral of emergency cases is essential in preventing complications, results from EI showed that only 10.73% (n=59) of all the women interviewed in the EI, had been referred for additional care. Most had been referred from level 3 facilities (n=26), using an ambulance (n=22) or public means (n=15), and were mainly accompanied by their husbands (n=27), relatives (n=23) or health workers (n=21) either as an individual or both at the same time (Appendix 2). A majority of the mothers' companions had knowledge of emergency management (n=47), were allowed to stay in the hospitals (n=33) and were warmly received at the hospitals (n=19) during the referral (Appendix 2).

For peer review only

The women in the FGDs perceived that the maternal services provided by the mothers had improved because of the LM policy, leading to a reduction in referrals:

> *'**R3:** I can say the services are good because nowadays we don't run to [referral hospital] the way we used to. So, this hospital has been good, it has been helpful to us.'* – ***(Woman in FGD003).***

The lack of equipment was the main reason for referral, and most women sought their own referral means from the hospital. From the EI, the referred mothers noted that lack of equipment, theatre, NBU and blood (n=16) were the leading cause of referral, followed by foetal distress (n=7) (Appendix 3). Whereas HCWs indicated that the county and facilities provide some form of referral transport for mothers, the referred mothers reported seeking their transport means for referral. These mothers perceived this to be dangerous for their health and safety and expensive, especially in unplanned emergencies.

> *'**R5:** …they [health workers] told me there's no vehicle, and they insist, "Look for a vehicle quickly so she can be referred" …now to do it fast and you don't have money…I really suffered; R8:…if a mother delivers now, [and]…is going to [a referral facility] and you know the road there is not good and someone has been stitched up down there [episiotomy]…when going there the stitches might be undone…'* – ***(Women in FGD009)***

Safety

Because of the policy, the facilities were managing complications better. HCWs and hospital administrations acknowledged that the policy improved the facilities' management of complications. The policy objectives incentivised them:

> *'…for example, she [patient] comes up with a chronic infection, which means the administration will spend more money buying an expensive drug for her. But you see, the moment she comes on time, early enough, she knows, "I went to the clinic, I was told I cannot deliver normally." She will come here on time. So, she will be told, "The moment you have reached 40 weeks, go to the hospital," she will be here. We do her C-section very safely; it is very simple she goes home. NBU decongested here…also the chorioamnionitis are no longer there.'* – ***(R012, Department In-charge)***

HCWs were reducing the time they allocate per mother. Given the workload that the HCWs were facing, they were reducing the time they allocated to providing each mother with care, and even some lower-level facilities were sending away mothers for they had higher numbers of patients:

> *'Owing to the fact that the patient numbers are higher than the health workers, the burden on the health worker is greater. Meaning the time allocated per patient is less than required'* – ***(R005, Facility Level Manager)***

Element 3: Management and organisation

Availability of essential physical resources

The policy has improved the availability and standards of equipment and supplies. With the help of reimbursements from the free policy, the facilities reported to have had improvements in the availability of supplies and medical equipment. In fact, the facilities have kept reordering supplies to keep up with the demand:

For peer review only - http://bmjopen.bmj.com/site/about/guidelines.xhtml

'…we've not actually gone out of stock. But you find we have to keep reordering because the demand is more.' – ***(R020, Facility Level Manager)***

Further, it was shown that with the availability of equipment and supplies, the HCWs did not have to utilise substandard care or equipment. For instance, one facility has shown how they had now departmentalised the sterilisation process of the equipment rather than using the hospital steriliser. With this came the availability of delivery packs, and they are no longer using ordinary blades as before:

'…we have so many like delivery packs which we used not to have. Sometimes we used to…. Use a blade instead of a delivery pack or the scissors because these things were not there…. There are people who are employed to cater for washing those things…and take…them [to] utility for preparation for next use.' – ***(R014, Nursing Officer)***

The facilities had improved infrastructure due to LM. Some facilities had used the reimbursements from the policy to improve infrastructure such as theatre and ultrasound areas. Additionally, some were expanding their buildings to reduce congestion. For instance, one facility had been able to complete a section of an incomplete building and transfer mothers to it from the congested postnatal ward:

'…when our mothers are many in this maternity [in facility C]…those without complications or those who had delivered yesterday, we transfer them to that department, so there is that decongestion. And we have another building there, the reproductive health, it is only that it is not yet over [complete]…but now the patients who are being attended…were transferred to that department and…we got the extension.' – ***(R014, Nursing Officer)***

Other facilities even renovated older buildings that were no longer in use and converted them into maternity clinics to ease congestion. For instance, in facility B, one building constructed five years ago to be a mortuary and was only being used to store patients' records, has now been refurbished and is used as an outpatient clinic. The downside was that the mothers had a negative attitude towards it as they believed it was still a mortuary.

Additionally, the policy reimbursements were helping facilities to meet their essential services that were critical in easing the burden of work. As noted by HCWs, they could incentivise mothers by using elements such as transport that would help improve quality. However, with more patients came more workload:

'…sometimes that money will help to fuel the vehicle and…to maintain the ambulance…[and] sometimes it can support…staffs to go for seminars and…to conduct those in-reach…and also outreach services' – ***(R008, Nursing Officer)***

'….in a way it's a pusher to more quality service to the client…because you want…to attract more…because the more, the better. But…that also has brought the issue of us bursting through the seams.' – ***(R019, Facility Level Manager)***

Enhanced facility resources and facility characteristics. The women in the EI ascertained that there was an enhancement of the resources in the facilities due to the policy. The facilities were shown to have adequate waiting and examination rooms (51.60%); adequate hand washing facilities (91.82%);

For peer review only - http://bmjopen.bmj.com/site/about/guidelines.xhtml

adequate bathing facilities (67.46%); adequate toilet facilities (71.45%); well-suited equipment for detecting women's problems (90.91%); had an adequate number of staff (76.37%) who are well suited to treat women (96.55%); and had an overall clean environment (93.45%) (Appendix 4). However, the mothers showed some concern about the adequacy of the facility providing clean drinking water, as indicated by 46.18% of mothers (Appendix 4).

Despite progress, some infrastructure, commodities, and supplies are still a challenge to some facilities. Some respondents noted that some facilities still have inadequate medical equipment (such as ultrasounds), space and supplies. The lack of these basic elements, such as a basic laboratory, was demotivating the women from using the services in the hospital and preventing HCWs from completely following up with the mothers as they would have wished to.

> *'…we don't have a very vibrant laboratory…as a clinician, I believe you want the patient tested, drugs availed, that patient will not come back to you after two days [said with wry humour]. You can give them a prescription, and they tell you they bought half a dose because they didn't have money, now, how will you help them? You see, it demotivates…… Yes. Even the ultrasound, the scans, we don't have the scans, so they have to do the scans outside [the facility]…About the [ward] it's not an ideal labour ward. We don't even have an ideal resuscitaire, you know, the improvised one?...you have to be extra cautious not to shake that thing, so the heater falls on the baby. Imagine, you have three mothers delivering, and you deliver as you put there...In the process you can burn those babies as you go to pick the other one...so, you have to be extra cautious... Even IPC [infection prevention and control] becomes an issue.'* – ***(R018, Facility level manager)***

The noted challenge regarding the supplies was that the county government was focusing on improving infrastructure, which was visible to the women, and perceived it as a better investment, rather than supplies and medication. The HCWs posited that the medication posed the biggest headache, whose potential cause was the drug ordering protocol. The facilities had to wait for a certain number of days before receiving top up for their orders:

> *'…there is a protocol…because like our drugs are ordered through KEMSA for a certain period, by any chance those drugs are not enough…they get finished before that period, we have to wait for the other order. But usually, in a hospital like ours [high-level facility], sometimes we are given extra money like miscellaneous where you can purchase emergency. But even when you purchase emergency like drugs, we are able to purchase a start dose or a prophylaxis, for continuity, you find now you have to involve maybe the patient.'* – ***(R020, Facility level manager)***

Competent and motivated staff

Mothers have a strong positive perception of healthcare delivery characteristics by the HCWs. A majority of the mothers in the EI had a positive perception of the healthcare delivery characteristics. For instance, 95.27% perceived that the staff examined pregnant and postpartum women well; 95.45% noted that the staff were very capable of finding out what is wrong with mothers; 59.64% noted that staff prescribed drugs that are needed and that the drugs supplied by the health facility were good (58.37%) and the mothers could obtain the drugs from health facility easily (67.27%) (Appendix 4). In addition, 71,27% perceived that they received adequate Information on danger signs of delivery and postpartum (Appendix 4). Interestingly, 79.82% perceived that the facility provided privacy during vaginal examination and delivery and 84.70% believed that the procedure they received during ANC and delivery felt very much necessary (Appendix 4).

For peer review only - http://bmjopen.bmj.com/site/about/guidelines.xhtml

Nurses are multitasking and handling many roles amidst the challenge of human resource. Nurses, especially in the lower-level facility, were shown to be going over and above in their work, covered both night and day shifts in addition to handling other hospital consultations at night, and still accompanied the pregnant women during referral. Despite the tasks being their roles, the constrained number of nurses was making the staff rotation allocation challenging, and hence, they had to multitask amidst the challenges.

Besides, because of the challenges of the increased workload from the LM policy, even the nurses in charge of both department and hospital administrations were forced to do the actual hands-on nursing practice rather than just stay in the office doing administrative work to ensure that the services are timely provided. Also, the nurses in the maternity wing asked for help from other departments when the work became overwhelming:

> *'…there is also the issue of shortage. Like today, we are so many, but at least we have covered all areas. But other times we report like three people, so…we have to work here and go to that place [to work in the wards]'* – ***(R007, Department in-charge)***

> *'We call help from other departments when it's so much.'* – ***(R001, Department in-charge)***

HCWs are adequately motivated to work despite the challenges. The HCWs reported being motivated to work more because they perceived that the more efforts that they put into providing service, the more the LM reimbursement funds the facility would make, which would subsequently translate to better services and additional hands (through locum nurses):

> *'…the policy of Linda mama has motivated the staff. At least we know that if you put more effort, there will be more funds on the facility, we will get more commodities, we will be compensated for escort [referral] and lunch…it will be more comfortable for us.'* – ***(R003, Nursing Officer)***

The hospital in-charges noted that despite the high workload, they feel that the HCWs are motivated and that they presented a perfect picture during supervision. For example, they noted that some were even comfortable running the wards alone without the support of other nurses and forfeiting their lunch time:

> *'…they go overboard [HCWs]…you would find two nurses on night duty, conducting 15-17 deliveries…alone. And finding this nurse has to monitor this mother from admission, delivery and postnatal and also the baby, you find they go overboard…like our nurses in maternity, they would not even break for lunch. They would wait until now the shift is over.'* – ***(R020, Facility level manager)***

Some mothers reported that the HCWs served them even when it was not their working shifts, which signified dedication to work:

> ***'R4:*** *I came here at 2:00 pm, and I got a doctor who was on the morning shift and the other one was changing. So, I told him to serve me, I wanted to deliver. He dressed in a hurry and came to help me.'* – ***(Woman in FGD003)***

In fact, the other cadre of HCWs, such as department clerical officers, noted that amidst the challenges, they are working beyond the stipulated hours either to support the provision of LM services or to work on the batching of the claims and ensure that the hospitals receive timely

For peer review only - http://bmjopen.bmj.com/site/about/guidelines.xhtml

reimbursement. However, they faced a challenge with inadequate and insufficient infrastructure (such as computers to ease work) and salaries. However, they perceived that they knew how to plan their days and work despite the challenges.

HCWs' source of motivation was more than just money. Some of the factors that the HCWs indicated as a source of motivation, rather than monetary values, were the kind acts and listening ear of the county administration and facility in-charges. For instance, in one facility, the department in charge felt that the administration provided them with a listening ear and acted on their grievances, including renovating the theatre and expanding the admission area. Others also felt that it resulted in the provision of adequate equipment and supplies to the facilities without having to improvise the old equipment:

> *'…at least we are listened to when we at least raise something…at least we get better service operating because of that. I mean theatre…was moved from here the squeezed area to that place, and then there wasn't bed, it was brought.'* – ***(R001, Department In-Charge)***

> *'…once in a while, we call them, have breakfast meetings with them, listen to their issues, discuss with them'* – ***(R016, County Senior Level Manager)***

The other source of motivation was that HCWs were happy when their burden of work was eased and department in-charges were doing so by employing additional people on locums, providing training opportunities, and recognising them for risking their lives at night during referrals to other facilities. Further, the nurses felt that they were involved in decision making and they perceived that it gave them a voice to raise an opinion on how the work needs to be done:

> *'So that one I see at least they could have involved us the people on the ground'* – ***(R014, Nursing Officer)***

There were some causes of demotivation and dissatisfaction among HCWs. For instance, HCWs noted that they felt inadequately remunerated despite the increased workload from the policy. With the workload, others felt that they had to multitask (for instance, handle referrals at all hours of the night and still had to come back to the facility after referral to carry out their duties which were waiting for them, and which they felt they were not adequately motivated for):

> *'We are underpaid, yeah let me say that without fear because we do a lot of work. You see like the time you came into the office; I was so buried there. I have been sitting there since 7:30 am'* – ***(R011, Clerical Officer)***

Similarly, the in-charges of the maternity departments, who were also HCWs, noted that the lack of timely reimbursements from the LM policy demotivated them. With such delays, the in-charges were having a strained working relationship with the hospital suppliers and even banks:

> *'You are doing your services, and you are claiming, but you are not getting the benefit of your work, so it renders even demoralising the people [HCWs in] the maternity…the same might demoralise even the suppliers who do supply us with the goods…some of them do cut off deals with dealing with the facility. Because we do pay them very late, and sometimes, they attract interest in their banks.'* – ***(R006, Nursing Officer)***

Monitoring and continuous quality improvement

For peer review only - http://bmjopen.bmj.com/site/about/guidelines.xhtml

Nurses monitor the QoC provided through partographing and charting labour progress, though they face challenges. The nurses showed awareness of proper documentation of labour progress using a partograph to enhance quality care. However, they noted that they sometimes faced additional scenarios (presentations/ conditions from the patients, e.g., those from referrals or mothers who came in at the second phases of labour and delivered within a few minutes of admission) that they did not know how to document.

> *'although once in a while a file maybe there is a problem, but they try.....because you know a partograph is very important…I know maybe you have found challenges in those partographs when you were going through.'* – ***(R007, Department In-Charge)*****.**

Despite the challenges, the nursing in-charges and facility managers were organising additional education to staff on the pregnant women monitoring processes. The university students, who were posted to the facilities for training, or even nurses who had had more recent training, were tasked to provide additional education to the nurses as they had more recent knowledge.

Element 4: Experience of care

Overall, a majority of the mothers (84.2%) from the EI were completely satisfied with the services they received (hospital A (85.1%), B (80.9%) and C (85.2%) were completely satisfied with the services provided). A higher proportion of mothers in hospital C (74.4%) than B (66.7%) and A (74.1%), would consider future delivery in the same health facility (Figure 2).

[INSERT FIGURE 2 HERE]

Effective communication with the patients

Mothers perceived and experienced the positive interpersonal qualities of the HCWs. A majority of the mothers in the EI had a positive perception (agreeing and completely agreeing) about the HCWs as being very open (94.34%); compassionate (90.58%); respectful (95.46%); devoted adequate time to the mothers (94.18%); and are very honest (92.00%) (Appendix 4). Some mothers noted that the HCWs were empathetic, friendly, and reassuring. They appreciated the additional good treatment and sacrifices the HCWs made, such as warming food and additional support (such as bathing the baby and changing bedsheets and stained beddings) following the exhausting birth experience.

Some mothers appreciated being given priority in treatment, especially during emergencies by the doctors. In such circumstances, the firmness and decisiveness of the nurses were also perceived positively as being intent on preserving the lives of both the mother and baby. One mother was particularly impressed with the doctors who called for assistance in emergency scenarios when they were not able to handle them at the time:

> ***'R1:*** *when I came once I got a certain doctor and I think there was an emergency, and I was forced to wait but I did not take offence because…he called another doctor who came here and I saw they have experience because they just serve you.'* – ***(Woman in FGD003).***

Mothers were happy to have received information about emergency/ procedures, and training on breastfeeding, family planning, and baby care. Some mothers highlighted that since some doctors

For peer review only - http://bmjopen.bmj.com/site/about/guidelines.xhtml

explained to them the medical procedures they were to undergo; they were able to relieve some anxiety around birth especially:

> *'**R2:** The doctor was good, he told me how it [procedure] would be done, and I was good.' –* ***(Woman in FGD004).***

The nurses supported the mothers during breastfeeding, taught them how to breastfeed and even encouraged those with difficulties. Some hospitals even went further by demonstrating to the mothers through *YouTube* videos on the procedure of breastfeeding which they perceived as very useful and helpful. The facility in-charges acknowledged that they trained and empowered the nurses with breastfeeding knowledge to ensure that they in turn train the mothers:

> *'And once this nurse trains in the breastfeeding, she'll go back, we make it as a duty for her to be educating the mother on those…on breastfeeding' –* **(R020, Facility Level Manager).**

Besides breastfeeding, the mothers acknowledged being taught about family planning, how to wash the baby's cord, and what to do if the baby faced some complications, which they considered reassuring.

Inadequate preparation for birth by the HCWs. Some HCWs were perceived as not being well prepared to handle the birth of the baby, given that they never had the birth equipment readily laid or that some materials and supplies were not readily available. This ultimately resulted in birth complications such as amniotic fluid aspiration.

The lack of proper education and communication on expectations. Some mothers felt there was no clear communication on the immediate care after delivery, which created a knowledge gap and potentially made mothers make mistakes with medications that resulted in medical emergencies. For instance, one mother indicated:

> *'**R6:** For my child there was a time I put the Hexi-cord [cord cleaning medication] on their nose. I did not know; I asked my husband to pass me the medicine at night thinking it was a nose drip. So, we thought that was it and we administered to him, we were forced to bring the baby here at night.' –* ***(Woman in FGD001).***

Some HCWs were perceived as not being reassuring and unable to provide mothers with the expected reassurance:

> *'**R12:** "We have examined you; the baby is not close." You know, sometimes you feel the baby is close, and when it's time to deliver, many doctors and nurses came and told me, "Why are you disturbing us, you are standing on the floor. Climb the bed." I could not climb. They said, "We are referring you to [a referral hospital]." Now I said, oh my god what will I do? At that time, they started to insult me and told me, "Come here, you are going to deliver in the ward."' –* ***(Woman in FGD009).***

Respect and dignity

Food was perceived as inadequate in some hospitals. Some mothers revealed that despite having a good birth experience in the labour ward and not paying anything for the delivery, the food provided particularly by the support staff post-delivery was inadequate, untimely, and unwholesome. Some

For peer review only - http://bmjopen.bmj.com/site/about/guidelines.xhtml

mothers in some facilities highlighted that appetence for food could sometimes last for a whole night post-delivery and thus they resorted to having their relatives and family bring them food.

> *'**R2:** I didn't pay anything, though their food is too little for a pregnant woman. It's true, it's too little, a mother has delivered, that food…and then they serve it very early, when it reaches 9pm you are hungry again…Yes, I had to call home [for food] because I felt weak. R7: There was a day I stayed here without food the whole night. I wasn't given.' – **(Women in FGD009).***

However, the administration revealed that the instance of food inadequacy may have been caused by the support staff who, despite the facility planning for adequate food for the whole hospital patients, may have rationed the food further. Despite the inadequacy of food, some mothers acknowledged that the food was actually good:

> *'**R3:** Yeah, it was good, I ate good things, and even the bathroom was clean. The services there are good.' – **(woman in FGD003)***

There was over-crowding and bed-sharing leading to a lack of privacy (congestion), and a lack of essential equipment and supplies, altering the QoC. Congestion in the maternity department because of the expanded FMP was a crosscutting theme especially in the higher-level facilities. The lower-level facilities equally faced an increase in the number of mothers particularly for ANC and delivery, but the mothers did not share beds:

> *'**R3:** but the problem I found here is congestion…. The first three hours [following CS] … I slept on a bed alone, after three hours we were two people on the bed. And from there the room we were taken too we would sleep four people with children, six people like that in one bed…. Because I left there with a back problem because I cannot sleep, you are forced to sit, you sit for the child to sleep.' – **(woman in FGD005).***

Nonetheless, the hospitals gave bed priority to mothers who had had caeserian section (CS) over normal delivery and were allowed to sleep on the bed alone in space permitting incidences in addition to having a special monitoring room. In contrast, mothers who had given birth normally, were forced to share beds with other mothers or sleep on the floor with only the babies sleeping on the beds. The congestion in the public facilities forced the mothers to seek care elsewhere.

Also, despite there being the expanded FMP, the lack of basic essential equipment and space was also noted to be a key driver to poor QoC even in maternal and child health clinics for PNC:

> *'Go to MCH [maternal and child health clinic]… and see how babies are weighed naked outside, in this harsh weather. It is at times very cold in the morning but what do we do, we have to weigh them….but we are glad that we are still able to offer services' – **(R018, Facility Level Manager).***

Emotional support

Women experienced physical, verbal, and emotional abuse. Some mothers experienced both physical and verbal abuse from HCWs and support staff. The abuse was exacerbated by the lack of clarity in communication with HCWs. For instance, one woman reported that the nurses had slapped her for being stubborn and uncooperative during birth, another woman mentioned that the nurse had tried to suture her episiotomy without using anaesthesia, and still another received abuse in return from either support staff or HCWs for requesting support:

For peer review only - http://bmjopen.bmj.com/site/about/guidelines.xhtml

*‘**R6:** I was slapped here…. For being stubborn; R3: you see someone is still in pain, they do not inject you with anaesthesia and they want to stitch you. Things like that are not good, this is also a human being, and they still feel pain. R5: I saw someone who had gone through a CS, and they told the nurse, they wanted to rise up, you know there is pain while rising up…but I saw her telling that nurse to help her get up, I saw [heard] the nurse insult her and I did not like that’ – **(Women in FGD001)***

Equally important was one mother’s testimony showing how she was wheeled to the theatre in a rather uncaring manner that lacked dignity:

*‘**R5:** What I saw, what he did to me, when I was experiencing labour pains, I was told to go to theatre, and I told him I cannot walk. He pushed me like a cart up to the theatre. I told him I could not walk; he pushed me like a lorry.’ – **(Woman in FGD008)***

Some mothers experienced a lack of attention/care, negligence and unhygienic practices from the HCWs and support staff. For instance, in one case a doctor was shown to have forgotten to remove cotton wool used in packing blood after delivery:

*‘**R6:** Like in my case they did not remove that thing [cotton wool] and then I went home with it.’ – **(Woman in FGD002)***

Additionally, some mothers perceived that some HCWs were not giving them and their babies proper attention while attending to them and they felt ignored. For instance, one respondent whose baby required medical oxygen felt a lack of support:

*‘**R6:** the baby came out fine. But I saw that by the time the nurse received him, he wasn’t breathing well and then the nurses did not care because when I woke up after six hours I had to go look after my baby, when the oxygen came out, I would put it back, I changed everything. So, this time round I did not like them.’ – **(Woman in FGD006)***

Some mothers were subjected to unhygienic practices by some HCWs, including being examined on an unclean bed previously used by another patient without wiping or being left unattended for long:

*‘**R4:** Another thing that I didn’t like there, you are examined on a bed that someone else had been examined on and it is damp. It wasn’t good. Like for me I was examined on a bed that had some liquid substance; **R9:** I delivered at [a referral hospital]; I didn’t like their services at all. Because when I delivered, I was cut down there [episiotomy] and the doctor left me for 30 minutes. On coming back, he stitched me with all that dirt, so I was not happy at all with their service.’ – **(Women in FGD009,).***

Some support staff also exacerbated the unhygienic practices of the mothers. For instance, one mother noted:

*‘**R6:** when I delivered here, I was asleep, when I woke up around 6.30. I found they [support staff] had opened windows as they wanted to clean. If you had put your bag on the floor, they ask you to pick it up and put it in bed and that bed is where you place the baby, and the ground is dirty.’ – **(Woman in FGD001).***

DISCUSSION

For peer review only - http://bmjopen.bmj.com/site/about/guidelines.xhtml

To our knowledge, this is the first study to examine the QoC across the continuum of maternal care (antenatal, perinatal, and postnatal care) under the expanded FMP or LM policy in Kenya.

Our findings show that the LM policy has reduced geographical access barriers by harnessing more private sector and faith-based facilities to enhance service provision. Furthermore, it has eliminated financial access barriers through the incentives of free maternal care and increased utilisation of maternal services (more mothers seek SBA; hence, reduced home deliveries). These findings align with results from systematic reviews of maternal services under different free maternity policies, which showed increased maternal (ANC and delivery) services after removing user fees (62, 63). Dossou et al.(64) also showed a systematic increase in CS services after implementing the CS policy in Benin because of utilisation incentives. However, the reviews showed that the utilisation patterns under free policies were marred by geographical and temporal fluctuations in use, which differs from our study.

Further, despite the policy enhancing access, the facilities were using additional approaches and incentives to attract mothers, leading to a difference in perception of the services provided. The finding on factors leading to the choice of the delivery place is not new, as other authors have highlighted the difference in the preference for private or public facilities thus influencing perception (65-67). In fact, in a recent FGD with women in Nairobi's informal settlements in Kenya, exploring their experiences of the quality of maternity care under LM, Oluoch-Aridi et al.(68) present the facilitators and barriers to choosing health facilities, which are all similar to the findings of this study. Interestingly, the choice of delivery site was influenced by several factors that are not necessarily related to LM, such as personal choice, previous experience or treatment, and access, as shown in other studies (4, 69) or health system factors (70). This highlights a key gap because it raises the question of whether LM has influenced the choice of hospital for delivery. Escamilla et al.(71) showed that the need for free services in Kenya had influenced women to bypass nearer facilities for farther private facilities that offered free care; which is similar to the findings from Sierra Leone by Fleming et al.(72)

Interestingly, our finding shows that while there was an increase in the utilisation of free maternal services, the facilities and HCWs bore the burden of providing service to more mothers seeking LM care. This finding aligns with other authors' findings, which have shown that there was a significant increase in the utilisation of maternal services following the implementation of the free policy in Kenya,(14, 73, 74) which was attributable to the removal of cost barriers to women (75). Nonetheless, our study goes further to highlight that despite bearing the burden, the facilities and the HCWs were shown to be working beyond their capacity to provide care to the extent that the HCWs ended up experiencing burnout. The unintended consequence of the increased burden on the HCWs could be explained by the fact that the implemented policy did not translate to an equal investment in an increased number of HCWs, hence the burden. Previous studies have shown that the perennial lack of human resources has always been a problem in Kenya. For instance, Miseda et al.(76) reveal that out of the 138,266 HCWs required to fit the MoH Norms and Standards Guidelines for service delivery, only 31,412 are employed at the public sector, private facilities, and faith-based organisations (FBOs).

It was also shown that the HCWs went beyond their strengths to serve the increased number of mothers well, as a way to maximise reimbursements from the LM policy, but this could cause burnout

For peer review only - http://bmjopen.bmj.com/site/about/guidelines.xhtml

if not followed by an increased workforce, thus leading to poor QoC. Two meta-analysis studies have shown that HCWs burnout could lead to the provision of poor QoC (77, 78). HCWs are motivated by what Franco et al.(79) deriving from Herzberg et al.(80) refer to as 'hygiene factors' (determining HCWs dissatisfaction) in this case the interpersonal relationship with the county and the administration, and 'motivating factor' (determining HCWs motivation and satisfaction) in this case being listened to. However, the facilities struggled to employ specialists and other HCWs staffing challenges.

Our study has highlighted the enhanced identification strategies for vulnerable populations (such as street children, orphans, and adolescents) that had initially been excluded from the policy on paper and are now using the policy. The findings align with the results of implementing the Safe Motherhood programme in Nigeria (Abiye initiative), which equally showed that removing user fees, particularly for the most vulnerable population, enhanced access and utility of service (81). However, in a different study in Kenya, researchers showed that the enhancement of the reach of the vulnerable population was mainly done by HCWs who, bound by ethics and professionalism, provided expanded FMP services to those excluded from the policy, such as foreigners, and those without IDs, such as street children who had no parents, refugees without IDs, or schoolgirls who were underage and pregnant. Hence, there is a need for official policy correction (82). While our results further show that there has been enhanced equity and financial access to the services by the women as those in the rural and urban areas received uniform services for free, in Benin, the CS policy exacerbated the inequalities as the policy reached the predominantly rich, exacerbating social exclusion (64).

Besides, from our findings, there is a positive perception of the policy despite the longer waiting times, particularly in the initial visits where mothers are accessing ANC additional benefit packages that were not in the previous policy. In contrast, a mixed-method study in Nigeria showed that mothers were dissatisfied with the waiting time under the free policy, but the authors did not link it to any particular service (83).

A rather interesting finding is the mothers' preference for higher-level facilities due to the perception of better services. Higher-level facilities are significantly burdened due to LM policy, leading to a ripple effect (where the facilities are left with a resource gap, as they use more resources to meet the mothers' specialised needs and manage deliveries that can be done at the periphery). However, it could also be argued that having more mothers in higher-level facilities means more claims and reimbursements. However, literature has attributed this preference to factors such as cleanliness, interpersonal skills, and other perceptions of better services;(84) and not the LM policy. A discrete choice experiment in Nigeria showed that the women chose to give birth in places with good condition of the health system, and absence of sexual, physical and verbal abuse, and that an unclean environment of birth without privacy and unclear user fees policy drove the women away (85). The mother's choice of higher-level facilities has led to QoC concerns such as indifference in the treatment based on the type of delivery and parity (partly because of overburdening higher facilities and the need for prioritisation). In Kenya, other studies have shown that mothers bypass lower-level facilities due to the perception of better quality (86, 87). Same case has been shown in Sri Lanka (88).

Interestingly, fewer mothers are being referred from lower to higher facilities than before the LM policy. While in the previous policy, complications were being referred to higher-level hospitals from

For peer review only - http://bmjopen.bmj.com/site/about/guidelines.xhtml

lower-level health centres to seek better services,(89) it could be argued that, through the LM policy, lower-level facilities are making adequate investments using the LM policy reimbursements and are thus able to handle complications. That may nevertheless not be true as another finding in our study showed that the fewer referrals that are happening are mainly due to the lack of equipment, theatre, and NBU in the lower-level facilities. Thus, it could be that the policy confusion in the reimbursements of the services is somewhat hampering the positive quality effects of the policy. Other literature from Ghana concurs with this assumption. For instance, Witter et al.'s(90) exploration of the policy showed that the uncoordinated and unreimbursed referral strategy (particularly at referring hospitals) hampered the positive effect of the policy, while Ganle et al.(91) showed that Ghana's referral system was ineffective and the care was substandard because of a lack of critical care staff to handle healthcare emergencies.

The mothers who are referred have a positive perception of the referral process. This perception could be because the HCWs went above and beyond to provide referral elements, such as allowing the mothers to have companions at referral time and in the hospital. However, the lack of transport for referral could hamper the referral gains by either making the mother pay or risk their life looking for transport systems at the tail end of delivery. For example, Burkina Faso included transport in their subsidy policy to enhance mothers' referrals to health facilities (92). Through its well-organised rapid response to emergency and evacuation, mothers were positively satisfied with the referral system under the policy; however, IDIs with HCWs revealed no adequate follow-up to ensure the evacuated mothers received care as intended (93). Interestingly, Kenyan nurses under the LM policy went above and beyond to refer and follow up mothers, which was a compensatory mechanism for improving QoC.

In addition, through the LM policy, there has been some improved availability of equipment, supply, and infrastructure. The improvement could be due to the provider and in-charges using Streel Level Beureacrat tacts (such as renovations) to improve the facility to attract more mothers who are the source of reimbursement funds. However, despite progress, some commodities, infrastructure, and supplies remain a challenge. The lack of supplies, equipment, and infrastructure contravenes the WHO statement number eight on quality, which shows that positive birth outcomes rely on their availability (41). A recent review showed that inadequacy is a global phenomenon compromising the quality of maternal care (94). Evidently, in all the facilities, the mothers revealed that they were satisfied with the characteristics of the facilities, such as having adequate rooms, adequate hand washing, bathing, and toilet facilities; in addition to equipment well suited for detecting women's problems. As is in this study, a mixed-methods study in Ghana showed that, despite the inadequate infrastructure in the facilities and lack of basic supplies, 89% of the mothers who participated in the EI, and those in the FGDs were satisfied with the quality of maternal care during childbirth (95) as is in this study. This postulates that mothers are more concerned about the interpersonal care received and the basic amenities provided if they can have live births and remain alive. The absence of or inadequacy of equipment and supplies compromises the QoC.

Equally interesting was that the good experience of care received by the women was based on the level of support provided by the HCWs and the facilities. Research shows that a good relationship between patients and HCWs could help improve trust, diffuse patients' anxieties, and create open communication (96). The majority of the mothers in both the FGDs and the EI attributed the good

For peer review only - http://bmjopen.bmj.com/site/about/guidelines.xhtml

experience of care to the interpersonal skills exhibited by the HCWs, such as empathy, being friendly, kindness, respect, devoting time, and honesty. The good care experiences the women receive influences their future delivery in the same facility. However, the findings could not show whether such experiences were due to LM policy, except that it incentivised the HCWs to provide FP and breastfeeding education. The finding shows that despite the challenges of the policy, the mothers appreciated and perceived the HCWs and health facility characteristics positively. This shows that HCWs have significantly contributed to the quality of provision of care, but this may not lead to improved outcomes if the technical aspects of quality are not met. Similar findings have been reported elsewhere where, for instance, in Ghana, 77% of the mothers who participated in the EI noted that they were content with the HCWs service provision as they were patient and empathetic (95) or in Ethiopia, where 79.1% of the mothers interviewed were happy with the overall services provided (97).

The poor experience of care by the mothers hampers the technical QoC received. By sharing the beds due to overcrowding, the mothers are exposed to unhygienic practices that could eventually lead to nosocomial infection in the maternity facilities, which hampers QoC. A review of quality elements in facilities in the 14 counties in Kenya linked the introduction of LM services with poor hygiene and low privacy (29) Such findings are expected because investments in hospital infrastructure have not subsequently followed the increase in the number of mothers utilising maternal care. Other literature has shown similar findings in other settings with FM services (98-100).

The other finding of poor QoC experienced by the mothers, such as lack of attention, negligence and physical abuse, has been shown in other Kenyan literature. For instance, the beneficiaries of FM services in a study in Kakamega provincial hospital in Kenya noted that the HCWs negligence and use of vulgar language were demeaning to the patients (101). Food is an important component in the birth process and for mothers to report that the food they received during delivery is inadequate is as surprising as it is demeaning. Also, as is in this study, poor communication with the mothers or lack thereof may create an ethical dilemma, especially in contexts where patients do not consent to or are not explained for procedures (102). Mothers should play a role in the decisions of the care provided.

A key limitation of this study is that the EIs were conducted in one county, and it is plausible that there could be varied practices across other counties. The implication of this study is that it may be difficult to generalise the findings to all the other 47 counties in Kenya. Nonetheless, using IDIs and FGDs in this study provides an opportunity to unpack the issue at hand (quality of maternal care under LM policy) within its context and be analytically generalisable. The meta-issues identified by the study are likely to be found in other counties, even though they might manifest in different ways.

CONCLUSION

This study has demonstrated that LM policy has provided positive results of quality across all the broad quality domains: access to care (equitable and timely), provision of care (safe and effective), management and organisation, and the experience of care. There were positive elements such as minimised access barriers (cultural, financial, geographic), timeliness of care, and provider availability that have created functional referral systems and safety, and availability of essential physical resources and competent and motivated staff. The women in the study had a good care experience, which included reception of prompt maternal services, good care for the baby after birth, teaching about birth procedures, breastfeeding, and family planning. Further, the results have shown negative

For peer review only - http://bmjopen.bmj.com/site/about/guidelines.xhtml

results from the policy hampering maternal care, such as the lack of supplies, equipment and infrastructure, and referral challenges. Cross-cutting poor experiences from the women exist, such as overcrowding of the healthcare facilities, inadequate food supply, the lack of communication of treatment plans, and experiencing both physical and verbal abuse. There is a need to address the negative aspects of the study while strengthening the positives to achieve the SDG and UHC goals that seek to ensure reduced maternal morbidities and mortalities through access to quality service for every woman.

Figure 1: Combined frameworks used in this study for examining the quality of care across the continuum of maternal care (antenatal, perinatal, and postnatal care) under the free maternity policy in Kenya.

Figure 2: Overall satisfaction and future delivery

List of abbreviations: ANC: Antenatal care; **CS:** Caeserian Section; **FBO:** faith-based organisations; **FGD:** Focus group discussions; **FMP:** Free maternity policy; **HCWs:** healthcare workers; **IDI:** Indepth interviews; **LM:** Linda mama; **LMIC:** Low-and-middle-income countries; **LIC:** low-income countries; **MCH:** maternal and child health; **QoC:** quality of care; **SBA:** Skilled birth attendance; **SSA:** sub-Saharan Africa; **UHC:** Universal Health Coverage; **WHO:** World Health Organization

Twitter: @bonnyoyugi_snr

Acknowledgements: The authors acknowledge the data collection support of the EI data from research assistants: Rachel Murigu, Justus Miran, Billy Bortich, Valentine Olunga, Janet Moraa, Winnie Kaitany, and Shillar Jeptoo.

Contributors: BO: conceptualised the study, curated and analysed the data, and drafted the initial manuscript which was subsequently revised for important intellectual content by all authors. ZAP: reviewed and edited all the drafts. SK and SP: contributed to the design and supervised the study. All authors read and approved the final manuscript.

Funding: This study was funded by the Commonwealth Scholarship Commission (KECS-2017-266), which supported BO's PhD study. The funding agency did not play any role in the analysis, interpretation of results, and manuscript writing.

Competing interests: None declared.

Patient consent for publication: Consent obtained directly from patient(s).

Patient and public involvement: Patients or the public were not involved in the design, or conduct, or reporting, or dissemination plans of our research.

Ethics approval: Ethical approval was obtained from the University of Kent, SSPSSR Students Ethics Committee and AMREF Scientific and Ethics Review Unit in Kenya (Ref: AMREF – ESRC P537/2018). Further written permission to conduct the study was received from the county government of Kiambu,

For peer review only

and all the participating hospitals. Written and oral informed consent was obtained from the potential participants before starting the interviews.

Data availability statement: The datasets used in this study are available from BO on b.oyugi@kent.ac.uk upon reasonable request.

Supplementary material: The authors have supplied this content.

For peer review only

REFERENCES

1. World Health Organization. Trends in maternal mortality 2000 to 2020: estimates by WHO, UNICEF, UNFPA, World Bank Group and UNDESA/Population Division Geveva: World Health Organization; 2023 [Available from: https://www.who.int/publications/i/item/9789240068759.
2. Black RE, Walker N, Laxminarayan R, Temmerman M. Reproductive, Maternal, Newborn, and Child Health: Disease Control Priorities Washington (DC): The International Bank for Reconstruction and Development / The World Bank; 2016 [Third Edition (Volume 2):[1]. Available from: https://www.ncbi.nlm.nih.gov/books/NBK361907/.
3. Kyei-Nimakoh M, Carolan-Olah M, McCann TV. Access barriers to obstetric care at health facilities in sub-Saharan Africa—a systematic review. Systematic reviews. 2017;6:1-16.
4. Gabrysch S, Campbell OM. Still too far to walk: literature review of the determinants of delivery service use. BMC pregnancy and childbirth. 2009;9(1):34.
5. Kenya National Bureau of Statistics, ICF. Kenya Demographic and Health Survey 2022. Key Indicators Report Nairobi, Kenya, and Rockville, Maryland, USA: KNBS and ICF; 2023 [Available from: https://dhsprogram.com/publications/publication-PR143-Preliminary-Reports-Key-Indicators-Reports.cfm.
6. UNICEF. Neonatal mortality: UNICEF; 2020 [Available from: https://data.unicef.org/topic/child-survival/neonatal-mortality/.
7. Kenya Ministry of Health. Reducing Maternal and Neonatal Mortality in Kenya: Scaling up Effective Interventions in Maternal and Newborn Health, An Implementation Plan for the period 2016 - 2018. In: Reproductive and Maternal Health Services Unit, Neonatal Child and Adolescent Health, Community Health Services Unit, Maternal and Newborn Health Technical Working Group, editors. Nairobi: Kenya Ministry of Health; 2016.
8. World Health Organisation. Trends in Maternal Mortality 2000 to 2017: Estimates by WHO, UNICEF, UNFPA, World Bank Group and the United Nations Population Division. Geneva: World Health Organization; 2019.
9. Chuma J, Maina T. Free Maternal Care and Removal of User Fees at Primary-Level Facilities in Kenya. Washington, DC: Health Policy Project, Futures Group; 2013.
10. Janisch C, Albrecht M, Wolfschuetz A, Kundu F, Klein S. Vouchers for health: a demand side output-based aid approach to reproductive health services in Kenya. Global Public Health. 2010;5(6):578-94.
11. Chuma J, Musimbi J, Okungu V, Goodman C, Molyneux C. Reducing user fees for primary health care in Kenya: policy on paper or policy in practice? International Journal for Equity in Health. 2009;8:15.
12. Chuma J, Okungu V. Viewing the Kenyan health system through an equity lens: implications for universal coverage. International Journal for Equity in Health. 2011;10(1):22.
13. Opwora A, Kabare M, Molyneux S, Goodman C. Direct facility funding as a response to user fee reduction: implementation and perceived impact among Kenyan health centres and dispensaries. Health Policy and Planning. 2010;25(5):406-18.
14. Tama E, Molyneux S, Waweru E, Tsofa B, Chuma J, Barasa E. Examining the implementation of the free maternity services policy in Kenya: a mixed methods process evaluation. International Journal of Health Policy and Management. 2018;7(7):603-13.
15. Orangi S, Kairu A, Ondera J, Mbuthia B, Koduah A, Oyugi B, et al. Examining the implementation of the Linda Mama free maternity program in Kenya. The International Journal of Health Planning and Management. 2021.
16. Oyugi B, Audi-Poquillon Z, Kendall S, Peckham S, Barasa E. The policy formulation process, and the role of actors in the policy formulation and implementation process: A policy analysis of the Kenyan free maternity policy. medRxiv. 2024:2024.01.26.23300268.
17. Ombere OS, Nyambedha OE, Haller T, Merten S. Local perspectives on policy implementation of free maternity health services in Kenya: Implications for universal health coverage. 2023.

For peer review only - http://bmjopen.bmj.com/site/about/guidelines.xhtml

18. National health Insurance Fund. Linda Mama Services Nairobi: National health Insurance Fund; 2017 [Available from: http://www.nhif.or.ke/healthinsurance/lindamamaServices.
19. Kenya Ministry of Health, National Hospital Insurance Fund. Implementation Manual for Programme Managers Nairobi: National Hospital Insurance Fund; 2016 [Available from: https://www.health.go.ke/wp-content/uploads/2018/11/implementation-manual-softy-copy-sample.pdf.
20. The Executive Office of the President. The Big 4 Agenda Nairobi: President's Delivery Unit; 2017 [Available from: https://big4.delivery.go.ke/.
21. World Health Organisation. The World Health Report 2010 Health Systems Financing: The path to universal coverage. Geneva: World Health Organization; 2010.
22. World Health Organisation. Arguing for Universal Health coverage. 2013.
23. Orangi S, Kairu A, Malla L, Ondera J, Mbuthia B, Ravishankar N, et al. Impact of free maternity policies in Kenya: an interrupted time-series analysis. BMJ Global Health. 2021;6(6):e003649.
24. Gitobu C, Gichangi P, Mwanda W. Patterns in maternal mortality following the implementation of a free maternal health care policy in Kenyan public health facilities. East African Medical Journal. 2017;94(6):433-44.
25. Gitobu C, Gichangi P, Mwanda W. Causes of neonatal mortality two years before and after the implementation of a free maternal health care policy in Kenyan public health facilities. East African Medical Journal. 2017;94(5):323-35.
26. Lang'at E, Mwanri L, Temmerman M. Effects of implementing free maternity service policy in Kenya: an interrupted time series analysis. BMC Health Service Research. 2019;19(1):645.
27. Oyugi B, Nizalova O, Kendall S, Peckham S. Does a free maternity policy in Kenya work? Impact and cost-benefit consideration based on demographic health survey data. Eur J Health Econ. 2023.
28. Oyugi B, Kendall S, Peckham S, Barasa E. Out of pocket payments during childbirth in Kenya under the free maternity services: Perspectives of mothers, healthcare workers and county officials. Wellcome Open Research. 2023.
29. Gitobu CM, Gichangi PB, Mwanda WO. Satisfaction with delivery services offered under the free maternal healthcare policy in Kenyan public health facilities. Journal of Environmental and Public Health. 2018;2018:4902864.
30. Owiti A, Oyugi J, Essink D. Utilization of Kenya's free maternal health services among women living in Kibera slums: a cross-sectional study. Pan Afr Med J. 2018;30:86.
31. Lusambili AM, Naanyu V, Wade TJ, Mossman L, Mantel M, Pell R, et al. Deliver on Your Own: Disrespectful Maternity Care in rural Kenya. PLOS ONE. 2020;15(1):e0214836.
32. Donabedian A. The quality of care: how can it be assessed? The Journal of the American Medical Association. 1988;260(12):1743-8.
33. Donabedian A. The seven pillars of quality. Archives of Pathology and Laboratory Medicine. 1990;114(11):1115-8.
34. Owili PO, Muga MA, Mendez BR, Chen B. Quality of maternity care and its determinants along the continuum in Kenya: A structural equation modeling analysis. PLoS One. 2017;12(5):e0177756.
35. Kruk ME, Gage AD, Arsenault C, Jordan K, Leslie HH, Roder-DeWan S, et al. High-quality health systems in the Sustainable Development Goals era: time for a revolution. The Lancet Global Health. 2018;6(11):e1196-e252.
36. World Health Organization. The network for improving quality of care for maternal, newborn and child health: evolution, implementation and progress: 2017-2020 report. 2021.
37. Creswell JW, Clark VLP. Designing and Conducting Mixed Methods Research. Third edition ed. Los Angeles: Sage publications, Inc; 2017.
38. Kelley E, Hurst J. Health Care Quality Indicators Project: Conceptual Framework Paper: Organisation for Economic Co-operation and Development 2006 [cited 2018 13th May]. Available from: http://thuvien.thanglong.edu.vn:8081/dspace/bitstream/DHTL_123456789/3992/1/No.%2023%20H

For peer review only

For peer review only - http://bmjopen.bmj.com/site/about/guidelines.xhtml

ealthcare%20quality%20indicators%20project%20%E2%80%93%20Conceptual%20framework%20paper.pdf.
39. Akachi Y, Kruk ME. Quality of care: measuring a neglected driver of improved health. Bulletin of the World Health Organization. 2017;95(6):465.
40. World Health Organization. Quality of Care for Maternal and Newborn Health: A Monitoring Framework for Network Countries Avenue Appia 20, 1211 Genève, Switzerland: Department of Maternal, Newborn, Child and Adolescent Health (MCA), World Health Organization HQ; 2019 [Available from: https://www.who.int/publications/m/item/quality-of-care-for-maternal-and-newborn--a-monitoring-framework-for-network-countries.
41. World Health Organisation. Standards for Improving Quality of Maternal and Newborn Care in Health Facilities 20 Avenue Appia, 1211 Geneva 27, Switzerland: World Health Organisation; 2016 [Available from: https://apps.who.int/iris/bitstream/handle/10665/249155/9789241511216-per.pdf.
42. Kenya Ministry of Health. Kenya Community Health Policy 2020-2030. In: Division of Community Health, editor. Nairobi, Kenya: Kenya Ministry of Health; 2020.
43. Kenya National Bureau of Statistics. 2019 Kenya Population and Housing Census Volume I: Population by County and Sub-County Nairobi: Kenya National Bureau of Statistics; 2019 [cited 2020 27 March]. Available from: https://www.knbs.or.ke/?wpdmpro=2019-kenya-population-and-housing-census-volume-i-population-by-county-and-sub-county.
44. County Government of Kiambu. About Kiambu County 2018 [cited 2020 27 March]. Available from: https://kiambu.go.ke/about-us/#2.
45. Kenya National Bureau of Statistics, Kenya Ministry of Health, National AIDS Control Council, Kenya Medical Research institute, National Council for Population and Development, ICF international. Kenya Demographic and Health Survey 2014 Calverton, MD: Kenya National Bureau of Statistics & ICF international; 2014 [cited 2020 5th May]. Available from: https://dhsprogram.com/pubs/pdf/fr308/fr308.pdf.
46. Kruk ME, Leslie HH, Verguet S, Mbaruku GM, Adanu RMK, Langer A. Quality of basic maternal care functions in health facilities of five African countries: an analysis of national health system surveys. The Lancet Global Health. 2016;4(11):e845-e55.
47. Kiambu County Government. Health services Kiambu: Midas Africa; 2024 [Available from: https://kiambu.go.ke/departments/health-services/.
48. Kahenda M. They live in Nairobi, but give birth in Kiambu Nairobi: The Standard; 2023 [Available from: https://www.standardmedia.co.ke/health/health-science/article/2001442841/they-live-in-nairobi-but-give-birth-in-kiambu.
49. Kamau KJ, Osuga BO, Njuguna S. Challenges facing implementation of referral system for quality health care services in Kiambu county, Kenya. 2017.
50. Okoroafor SC, Kwesiga B, Ogato J, Gura Z, Gondi J, Jumba N, et al. Investing in the health workforce in Kenya: trends in size, composition and distribution from a descriptive health labour market analysis. BMJ Global Health. 2022;7(Suppl 1):e009748.
51. Perfomance Monitoring for Action. Kenya (Kiambu): Results from phase 2 cross-sectional survey - (section 1: contraceptive use, dynamic, and demand) 2020 [Available from: https://www.pmadata.org/sites/default/files/data_product_results/Kenya%20Kiambu_Phase%202%20XS%20Results_Brief_final.pdf.
52. Oyugi B. The Policy Process, Quality and Cost of Free Maternal Healthcare in Kenya: A Mixed Methods Analysis of Maternity Policy: University of Kent, Centre for Health Services Studies, University of Kent; 2021.
53. Muchemi OM, Gichogo AW, Mungai JG, Roka ZG. Trends in health facility based maternal mortality in Central Region, Kenya: 2008-2012. Pan African Medical Journal. 2016;23(1).
54. Hennink MM, Kaiser BN, Marconi VC. Code saturation versus meaning saturation: how many interviews are enough? Qualitative Health Research. 2017;27(4):591-608.
55. Gorstein J, Sullivan K, Parvanta I, Begin F. Indicators and Methods for Cross-Sectional Surveys of Vitamin and Mineral Status of Populations: The Micronutrient Initiative (Ottawa) and the Centers

For peer review only - http://bmjopen.bmj.com/site/about/guidelines.xhtml

for Disease Control and Prevention (Atlanta); 2007 [Available from: https://www.who.int/vmnis/toolkit/mcn-micronutrient-surveys.pdf.
56. Dalinjong PA, Wang AY, Homer CSE. The operations of the free maternal care policy and out-of-pocket payments during childbirth in rural Northern Ghana. Health Economics Review. 2017;7(1):41.
57. StatTrek.com. Random Number Generator: Stat Trek; 2021 [cited 2019 20 October]. Available from: https://stattrek.com/statistics/random-number-generator.aspx.
58. Kenya Ministry of Health. Kenya Master Health Facility List (KMHFL): Kenya Ministry of Health,; 2020 [Available from: http://kmhfl.health.go.ke/#/home.
59. DHIS2. Kenya Health Information System (KHIS) for Aggregate Reporting: DHIS2; 2020 [cited 2020 16 March]. Available from: https://hiskenya.org/dhis-web-commons/security/login.action.
60. Ritchie J, Spencer L. Qualitative data analysis for applied policy research. In: Bryman A, Burgess RG, editors. Analysing Qualitative Data. New York, NY: Routledge, Taylor & Francis Group; 2002. p. 173-94.
61. Yin KR. Case Study Research and Applications: Design and Methods. 6th ed. Thousand Oaks, CA: SAGE Publications; 2018.
62. Dzakpasu S, Powell-Jackson T, Campbell OM. Impact of user fees on maternal health service utilization and related health outcomes: a systematic review. Health Policy and Planning. 2014;29(2):137-50.
63. Oyugi B, Kendall S, Peckham S. Effects of free maternal policies on quality and cost of care and outcomes: an integrative review. Primary health care research & development. 2021;22.
64. Dossou J-P, Cresswell JA, Makoutodé P, De Brouwere V, Witter S, Filippi V, et al. 'Rowing against the current': the policy process and effects of removing user fees for caesarean sections in Benin. BMJ Global Health. 2018;3(1):e000537.
65. Okumu C, Oyugi B. Clients' satisfaction with quality of childbirth services: a comparative study between public and private facilities in Limuru Sub-County, Kiambu, Kenya. PLoS One. 2018;13(3):e0193593.
66. Chirdan O, Lar L, Afolaranmi T, Inalegwu E, Igoh C, Adah G. Client satisfaction with maternal health services comparism between public and private hospitals in Jos Nigeria. Jos Journal of Medicine. 2013;7(1):1-9.
67. Khan REA, Noreen S. Household choice of public versus private health institution for maternal health-care: a case study of Bahawalpur (Pakistan). Pakistan Journal of Commerce and Social Sciences. 2016;10(3):444-60.
68. Oluoch-Aridi J, Wafula F, Kokwaro G, Adam MB. 'We just look at the well-being of the baby and not the money required': a qualitative study exploring experiences of quality of maternity care among women in Nairobi's informal settlements in Kenya. BMJ Open. 2020;10(9):e036966.
69. Amooti-Kaguna B, Nuwaha F. Factors influencing choice of delivery sites in Rakai district of Uganda. Social Science & Medicine. 2000;50(2):203-13.
70. Parkhurst JO, Penn-Kekana L, Blaauw D, Balabanova D, Danishevski K, Rahman SA, et al. Health systems factors influencing maternal health services: a four-country comparison. Health Policy. 2005;73(2):127-38.
71. Escamilla V, Calhoun L, Winston J, Speizer IS. The role of distance and quality on facility selection for maternal and child health services in urban Kenya. Journal of Urban Health. 2018;95(1):1-12.
72. Fleming LC, Ansumana R, Bockarie A, Alejandre J, Bangura U, Jimmy DH, et al. Inpatient healthcare provider bypassing by women and their children in urban Bo, Sierra Leone. The Pan African medical journal. 2016;23:146.
73. Pyone T, Smith H, van dB. Implementation of the free maternity services policy and its implications for health system governance in Kenya. BMJ Global Health. 2017;2(4):e000249.

For peer review only

74. Lang'at E, Mwanri L. Healthcare service providers' and facility administrators' perspectives of the free maternal healthcare services policy in Malindi District, Kenya: a qualitative study. Reproductive Health. 2015;12:59.
75. Njuguna J, Kamau N, Muruka C. Impact of free delivery policy on utilization of maternal health services in county referral hospitals in Kenya. BMC Health Services Research. 2017;17(1):429.
76. Miseda MH, Were SO, Murianki CA, Mutuku MP, Mutwiwa SN. The implication of the shortage of health workforce specialist on universal health coverage in Kenya. Human Resources for Health. 2017;15(1):80.
77. Tawfik DS, Scheid A, Profit J, Shanafelt T, Trockel M, Adair KC, et al. Evidence relating health care provider burnout and quality of care: a systematic review and meta-analysis. Annals of Internal Medicine. 2019;171(8):555-67.
78. Salyers MP, Bonfils KA, Luther L, Firmin RL, White DA, Adams EL, et al. The relationship between professional burnout and quality and safety in healthcare: a meta-analysis. Journal of General Internal Medicine. 2017;32(4):475-82.
79. Franco LM, Bennett S, Kanfer R. Health sector reform and public sector health worker motivation: a conceptual framework. Social Science & Medicine. 2002;54(8):1255-66.
80. Herzberg F, Mausner B, Snyderman B. The Motivation to Work. 2 ed. New York: John Wiley & Sons; 1959.
81. Ajayi AI, Akpan W. Maternal health care services utilisation in the context of 'Abiye' (safe motherhood) programme in Ondo State, Nigeria. BMC Public Health. 2020;20(1):362.
82. Oyugi B, Kendall S, Peckham S, Orangi S, Barasa E. Exploring the Adaptations of the Free Maternity Policy Implementation by Health Workers and County Officials in Kenya. Global Health: Science and Practice. 2023;11(5).
83. Ajayi AI. "I am alive; my baby is alive": understanding reasons for satisfaction and dissatisfaction with maternal health care services in the context of user fee removal policy in Nigeria. PLoS One. 2019;14(12).
84. Oyugi B, Kioko U, Kaboro SM, Okumu C, Ogola-Munene S, Kalsi S, et al. A facility-based study of women'satisfaction and perceived quality of reproductive and maternal health services in the Kenya output-based approach voucher program. BMC pregnancy and childbirth. 2018;18(1):310.
85. Umar N, Quaife M, Exley J, Shuaibu A, Hill Z, Marchant T. Toward improving respectful maternity care: a discrete choice experiment with rural women in northeast Nigeria. BMJ Glob Health. 2020;5(3):e002135.
86. Audo M, Ferguson A, Njoroge P. Quality of health care and its effects in the utilisation of maternal and child health services in Kenya. East African Medical Journal. 2005;82(11):547.
87. Cohen J, Golub G, Kruk ME, McConnell M. Do active patients seek higher quality prenatal care?: a panel data analysis from Nairobi, Kenya. Preventive Medicine. 2016;92:74-81.
88. Perera S, Weerasinghe M. Bypassing primary care in Sri Lanka: a comparative study on reasons and satisfaction. Vietnam Journal of Public Health. 2015;3(1):69-76.
89. Sidze EM, Fenenga C, Amendah DD, Maina TM, Mutua MK, Mulupi SK, et al. Are free maternal healthcare services programs an impediment to quality care? an examination of the Kenyan experience. African Population and Health Research Center. 2015;5(1):1-10.
90. Witter S, Arhinful DK, Kusi A, Zakariah-Akoto S. The experience of Ghana in implementing a user fee exemption policy to provide free delivery care. Reproductive Health Matters. 2007;15(30):61-71.
91. Ganle JK, Parker M, Fitzpatrick R, Otupiri E. A qualitative study of health system barriers to accessibility and utilization of maternal and newborn healthcare services in Ghana after user-fee abolition. BMC pregnancy and childbirth. 2014;14(1):434-62.
92. Ridde V, Queuille L, Kafando Y, Robert E. Transversal analysis of public policies on user fees exemptions in six West African countries. BMC Health Services Research. 2012;12:409.
93. Ridde V, Diarra A. A process evaluation of user fees abolition for pregnant women and children under five years in two districts in Niger (West Africa). BMC Health Services Research. 2009;9:1-16.

For peer review only - http://bmjopen.bmj.com/site/about/guidelines.xhtml

94. Bohren MA, Vogel JP, Hunter EC, Lutsiv O, Makh SK, Souza JP, et al. The mistreatment of women during childbirth in health facilities globally: a mixed-methods systematic review. PLoS Medicine. 2015;12(6):e1001847.
95. Dalinjong PA, Wang AY, Homer CS. Are health facilities well equipped to provide basic quality childbirth services under the free maternal health policy? findings from rural Northern Ghana. BMC Health Services Research. 2018;18(1):959.
96. Okoror CEM, Enabudoso EJ, Okoroh MI. Women's perception and satisfaction with the quality of antenatal care services in mission hospitals in Benin City, Nigeria. Pyramid Journal of Medicine. 2020;3(1).
97. Tesfaye R, Worku A, Godana W, Lindtjorn B. Client satisfaction with delivery care service and associated factors in the public health facilities of Gamo Gofa zone, Southwest Ethiopia: in a resource limited setting. Obstetrics and Gynecology International. 2016;2016:1-7.
98. Masaba BB, Mmusi-Phetoe RM. Free maternal health care policy in Kenya: level of utilization and barriers. International Journal of Africa Nursing Sciences. 2020;13:100234.
99. Chesumei EJ. Factors associated with uptake of free maternity services at Baringo County Referral Hospital: Jomo Kenyatta University of Agriculture and Technology; 2019.
100. Witter S, Adjei S, Armar-Klemesu M, Graham W. Providing free maternal health care: ten lessons from an evaluation of the national delivery exemption policy in Ghana. Global Health Action. 2009;2(1):1881.
101. Asule BM, Kwena A, Wambui T. Effects of the free maternity care program on utilization of services at a county referral hospital in Kenya. Kenyan Journal of Nursing & Midwifery. 2017;1(2).
102. Khumalo N, Rwakaikara E. Patient satisfaction with peri-partum care at Bertha Gxowa district hospital, South Africa. African Journal of Primary Health Care & Family Medicine. 2020;12(1):a2409.

For peer review only

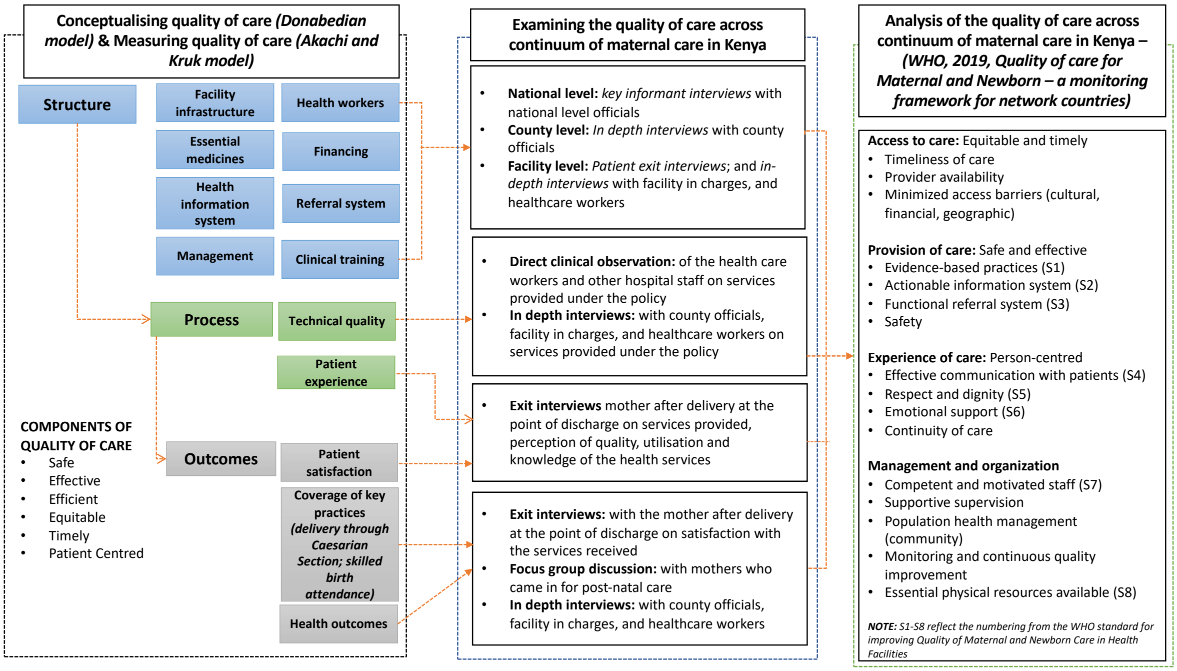

For peer review only - http://bmjopen.bmj.com/site/about/guidelines.xhtml

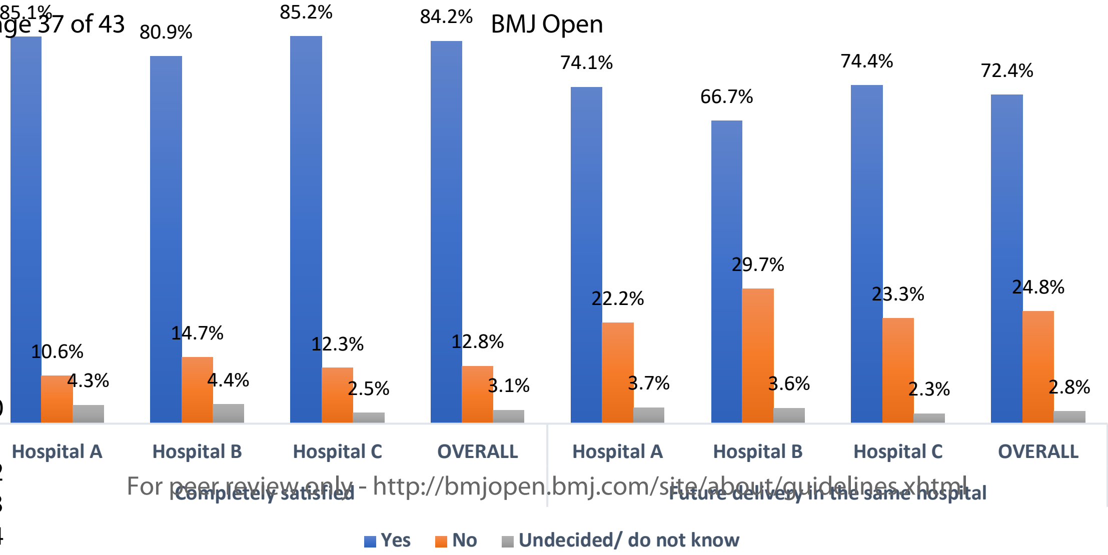

For peer review only - http://bmjopen.bmj.com/site/about/guidelines.xhtml

Appendix 1: Maternal healthcare access characteristics

Variable		Total Frequency (%) n=550	Hospital A n (%) n=42	Hospital B n (%) n=170	Hospital C n (%) n=338	p-value
Facility visited during pregnancy	Yes	545 (99.09)	42 (100)	169 (99.41)	334 (98.82)	0.650
	No	5 (0.91)	0	1 (0.59)	4 (1.18)	
Type of facility visited	Public facility	506 (92.00)	38 (90.48)	149 (87.65)	319 (94.38)	**P<0.001***
	Private facility	28 (5.09)	1 (2.38)	17 (10.00)	10 (2.96)	
	Faith based organization (Mission)	7 (1.27)	-	3 (1.76)	4 (1.18)	
	Other	9 (1.64)	3 (7.14)	1 (0.59)	5 (1.48)	
Time taken to reach hospital	Below 30 minutes	137 (24.91)	12 (28.57)	45 (26.47)	80 (23.67)	0.309
	30 minutes-1 hour	264 (48.00)	19 (45.24)	87 (51.18)	158 (46.75)	
	1 hour-2 hours	121 (22.00)	7 (16.67)	35 (20.59)	79 (23.37)	
	More than 2 hours	20 (3.64)	4 (9.52)	2 (1.18)	14 (4.14)	
	Don't know	8 (1.45)	-	1(0.59)	7 (1.96)	
Perception of the time take to reach the hospital	Very short	60 (10.91)	6 (14.29)	25 (14.71)	29 (8.58)	0.340
	Short	249 (45.27)	18 (42.86)	73 (42.94)	158 (46.75)	
	Normal	99 (18.00)	12 (28.57)	32 (18.82)	55 (16.27)	
	Long	107 (19.45)	5 (11.90)	29 (17.06)	73 (21.60)	
	Very long	32 (5.82)	1 (2.38)	11 (6.47)	20 (5.92)	
	Don't know	3 (0.54)	-	-	3 (0.89)	
	Very near	69 (12.55)	16 (38.10)	22 (12.94)	31 (9.17)	**P<0.001**

For peer review only

Variable		Total Frequency (%) n=550	Hospital A n (%) n=42	Hospital B n (%) n=170	Hospital C n (%) n=338	p-value
Perception about distance to the facility	Normal	339 (61.64)	17 (40.48)	107 (62.94)	215 (63.61)	
	Far	110 (20.00)	8 (19.05)	33 (19.41)	69 (20.41)	
	Very far	28 (5.09)	-	8 (4.71)	20 (5.92)	
	Don't know	4 (0.73)	1 (2.38)	-	3 (0.89)	
Means of transport to the facility	Walking	27 (4.91)	7 (16.67)	14 (8.24)	6 (1.78)	**P<0.001**
	Bi/Motorcycle	60 (10.91)	1 (2.38)	20 (11.76)	39 (11.54)	
	Public transport (matatu/tuk tuk)	224 (40.73)	8 (19.05)	55 (32.35)	161 (47.63)	
	Private car/taxi	211 (38.36)	24 (57.14)	78 (45.88)	109 (32.25)	
	Ambulance	22 (4.00)	-	1 (0.59)	21 (6.21)	
	Combined modes	6 (1.09)	2 (4.76)	2 (1.18)	2 (0.59)	
Does opening hour suit your time?	Yes	431 (78.36)	41 (97.62)	152 (89.41)	238 (70.41)	**P<0.001**
	No	9 (1.64)	1 (2.38)	1 (0.59)	7 (2.07)	
	Don't know	76 (13.82)	-	15 (8.82)	61 (18.05)	
	N/A	34 (6.18)	-	2 (1.18)	32 (9.47)	
Waiting time at the facility	Very short	80 (14.55)	12 (28.57)	26 (15.29)	42 (12.43)	**P<0.001**
	Short	237 (43.09)	16 (38.10)	72 (42.35)	149 (44.08)	
	Normal	70 (12.73)	11 (26.19)	28 (16.47)	31 (9.17)	
	Long	80 (14.55)	1 (2.38)	22 (12.94)	57 (16.86)	
	Very long	43 (7.82)	2 (4.76)	22 (12.94)	19 (5.62)	
	N/A	40 (7.27)	-	-	40 (11.83)	

For peer review only

Variable		Total Frequency (%) n=550	Hospital A n (%) n=42	Hospital B n (%) n=170	Hospital C n (%) n=338	p-value
Hospital have a proper waiting area	Yes	422 (76.73)	40 (95.24)	134 (78.82)	248 (73.37)	**0.005**
	No	85 (15.45)	1 (2.38)	29 (17.06)	55 (16.27)	
	Don't know	28 (5.09)	1 (2.38)	7 (4.12)	20 (5.92)	
	N/A	15(2.73)	-	-	15 (4.44)	

Note: Chi square test of proportion was used to test difference in overall proportions of maternal health access characteristics.
*There is a statistical difference in the type of facilities that the mothers visited (majority visited public facilities).
Bold means **p-value <0.05**

For peer review only

Appendix 2: Referral characteristics

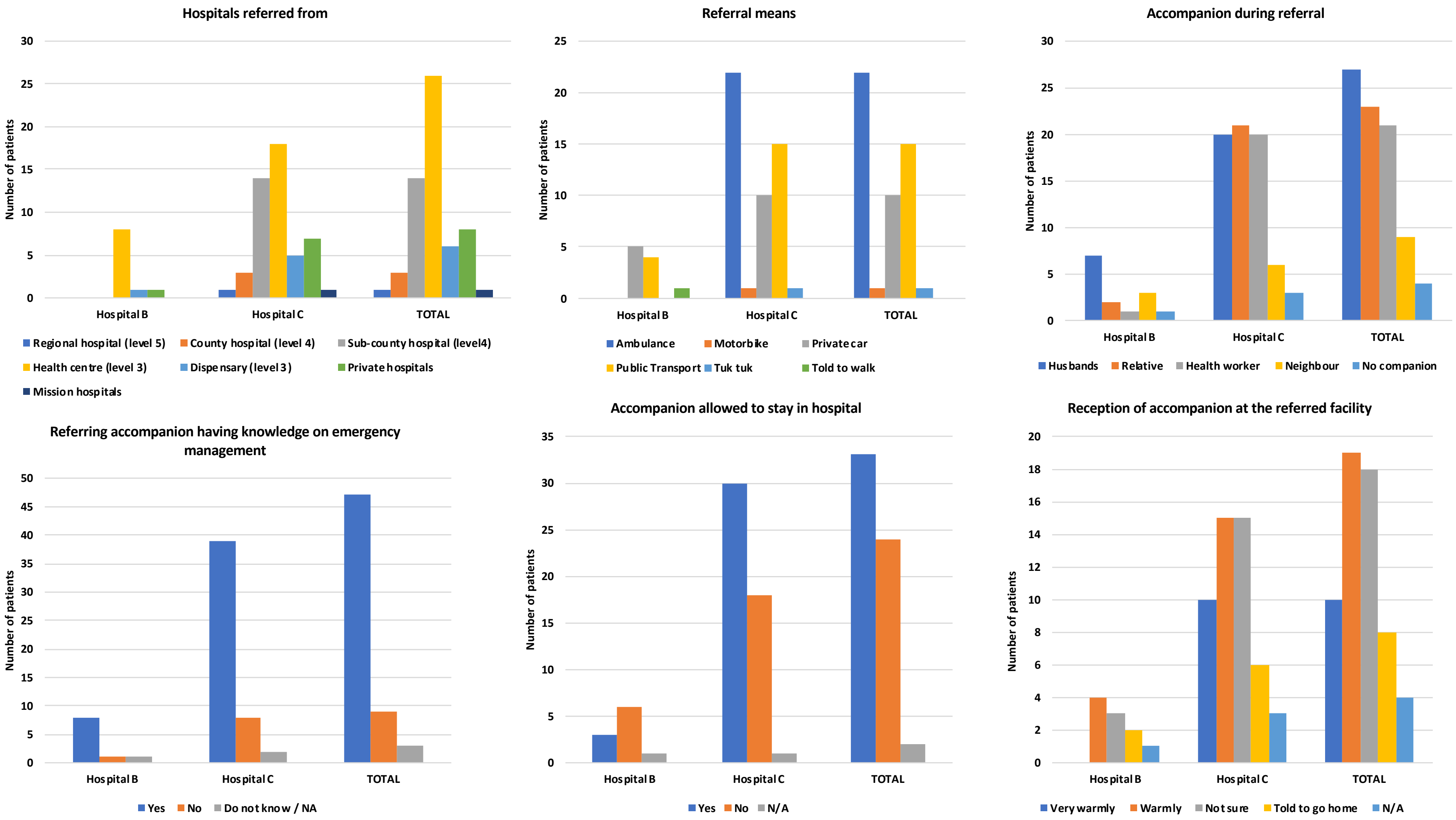

Appendix 3: Reasons for referral

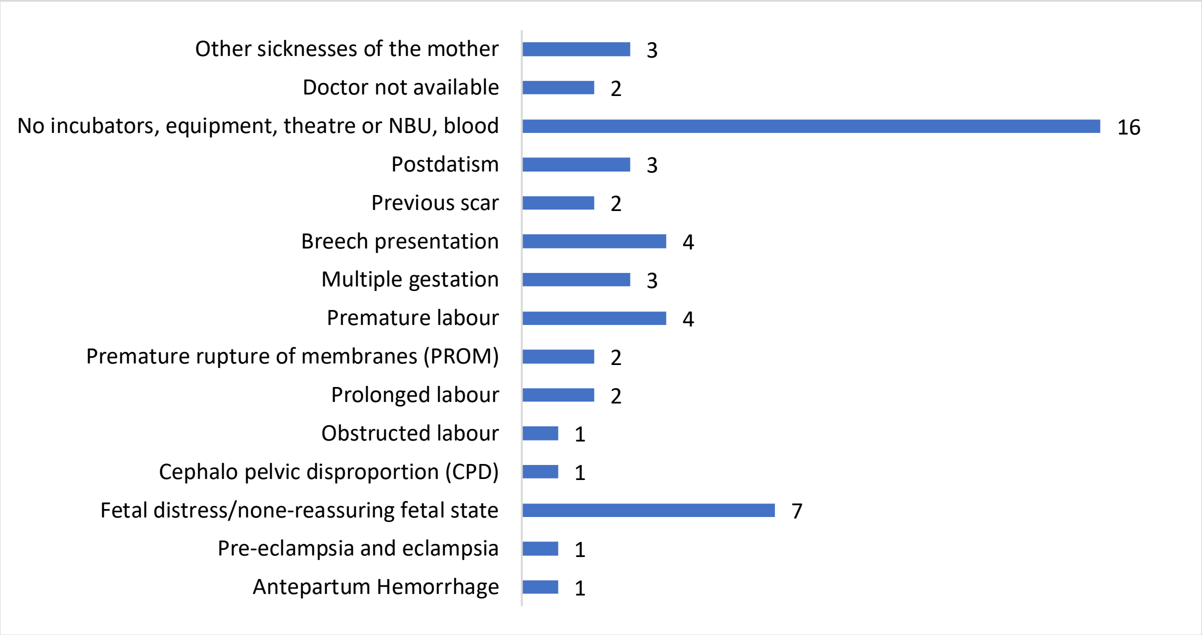

For peer review only - http://bmjopen.bmj.com/site/about/guidelines.xhtml

Appendix 4: Perception of quality of maternal care from the mothers exit interviews

Health Facility	Completely Disagree	Disagree	Not Sure	Agree	Completely Agree
Number of staff adequate	0.73%	19.09%	3.82%	57.82%	18.55%
Staff well suited to treat women	0.36%	1.82%	1.27%	70.73%	25.82%
Waiting and examination rooms adequate	5.82%	38.73%	3.82%	40.55%	11.05%
Provision of clean drinking water adequate	3.82%	38.36%	11.46%	34.00%	12.18%
Hand washing facilities adequate	1.45%	6.00%	0.73%	70.55%	21.27%
Bathing facilities adequate	3.82%	24.36%	4.36%	52.91%	14.55%
Toilet facilities adequate	2.91%	24.00%	1.64%	55.45%	16.00%
Overal facility environment very clean	0.91%	3.64%	2.00%	70.00%	23.45%
Well suited equipment for detecting women's problems	0.91%	3.45%	4.73%	69.82%	21.09%
Distance from home very far	8.55%	59.09%	2.18%	23.82%	6.36%
**Healthcare delivery**	**Completely Disagree**	**Disagree**	**Not Sure**	**Agree**	**Completely Agree**
Staff examine pregnant and post partum women well	0.91%	2.00%	1.82%	72.00%	23.27%
Staff very capable of finding out what is wrong with patients	0.73%	1.64%	2.18%	71.09%	24.36%
Staff prescribe drugs that are needed	0.00%	2.91%	37.45%	42.91%	16.73%
Drugs supplied by health facility are good	0.36%	1.45%	39.82%	42.55%	15.82%
Patients can obtain drugs from health facility easily	1.45%	5.64%	25.64%	52.00%	15.27%
Facility provided privacy very much during VE and delivery	3.82%	9.64%	6.73%	63.82%	16.00%
Felt very much of necessary procedure during ANC and delivery	3.83%	8.38%	3.10%	65.39%	19.31%
Adequate Information on danger signs of delivery and postpartum	1.45%	0.24%	3.27%	49.45%	21.82%
**Interpersonal Aspects**	**Completely Disagree**	**Disagree**	**Not Sure**	**Agree**	**Completely Agree**
Staff very open with the patients	0.18%	3.83%	1.64%	68.61%	25.73%
Staff very compasionate towards the patients	1.27%	5.45%	2.73%	66.00%	24.58%

For peer review only

Staff are respectful towards the patients	0.18%	2.73%	1.64%	69.64%	25.82%
Time staff devete to the patients is adequate	0.36%	4.36%	1.09%	67.27%	26.91%
Staff are very honest	0.00%	2.36%	5.64%	65.64%	26.36%

For peer review only

BMJ Open

Examining the quality of care across the continuum of maternal care (antenatal, perinatal, and postnatal care) under the expanded free maternity policy (Linda Mama Policy) in Kenya: a mixed-methods study

Journal:	*BMJ Open*
Manuscript ID	bmjopen-2023-082011.R2
Article Type:	Original research
Date Submitted by the Author:	04-Apr-2024

Complete List of Authors:	Oyugi, Boniface; M and E Advisory Group, P.O. Box 6523 - 00200, Western Heights, The Mint Nairobi; University of Kent, Centre for Health Services Studies<br>Audi-Poquillon, Zilper; The London School of Economics and Political Science, Department of Health Policy<br>Kendall, Sally; University of Kent, Centre for Health Services Studies<br>Peckham, Stephen; University of Kent, Centre for Health Services Studies

**Primary Subject Heading**:	Health services research
Secondary Subject Heading:	Evidence based practice, Health economics, Health policy, Health services research, Global health
Keywords:	Health economics < HEALTH SERVICES ADMINISTRATION & MANAGEMENT, Health policy < HEALTH SERVICES ADMINISTRATION & MANAGEMENT, Maternal medicine < OBSTETRICS, Quality in health care < HEALTH SERVICES ADMINISTRATION & MANAGEMENT, Community child health < PAEDIATRICS, HEALTH ECONOMICS

SCHOLARONE™
Manuscripts

I, the Submitting Author has the right to grant and does grant on behalf of all authors of the Work (as defined in the below author licence), an exclusive licence and/or a non-exclusive licence for contributions from authors who are: i) UK Crown employees; ii) where BMJ has agreed a CC-BY licence shall apply, and/or iii) in accordance with the terms applicable for US Federal Government officers or employees acting as part of their official duties; on a worldwide, perpetual, irrevocable, royalty-free basis to BMJ Publishing Group Ltd ("BMJ") its licensees and where the relevant Journal is co-owned by BMJ to the co-owners of the Journal, to publish the Work in this journal and any other BMJ products and to exploit all rights, as set out in our licence.

The Submitting Author accepts and understands that any supply made under these terms is made by BMJ to the Submitting Author unless you are acting as an employee on behalf of your employer or a postgraduate student of an affiliated institution which is paying any applicable article publishing charge ("APC") for Open Access articles. Where the Submitting Author wishes to make the Work available on an Open Access basis (and intends to pay the relevant APC), the terms of reuse of such Open Access shall be governed by a Creative Commons licence – details of these licences and which Creative Commons licence will apply to this Work are set out in our licence referred to above.

Other than as permitted in any relevant BMJ Author's Self Archiving Policies, I confirm this Work has not been accepted for publication elsewhere, is not being considered for publication elsewhere and does not duplicate material already published. I confirm all authors consent to publication of this Work and authorise the granting of this licence.

For peer review only

Examining the quality of care across the continuum of maternal care (antenatal, perinatal, and postnatal care) under the expanded free maternity policy (Linda Mama Policy) in Kenya: a mixed-methods study

Authors

Boniface Oyugi[1,2], **Zilper Audi-Poquillon**[3*], **Sally Kendall**[2], **Stephen Peckham**[2]

1. M and E Advisory Group, P.O. Box 6523 - 00200, Western Heights, The Mint Nairobi, Kenya.
2. Centre for Health Services Studies (CHSS), University of Kent, George Allen Wing, Canterbury CT2 7NF
3. Department of Health Policy, London School of Economics and Political Science

***Corresponding Author: Zilper Audi-Poquillon (ZAP)** – z.a.audi-poquillon@lse.ac.uk

Email addresses:
BO: b.oyugi@kent.ac.uk
SK: S.Kendall-608@kent.ac.uk
SP: S.Peckham@kent.ac.uk

ORCID IDs
Boniface Oyugi: https://orcid.org/0000-0002-9550-9138
Zilper Audi-Poquillon: https://orcid.org/0009-0002-6901-7348
Sally Kendall: https://orcid.org/0000-0002-2507-0350
Stephen Peckham: https://orcid.org/0000-0002-7002-2614

Qualification
BO: PhD (Dr)
ZAP: MSc (Ms)
SK: PhD (Professor)
SP: (Professor)

For peer review only

ABSTRACT

Background

Kenya still faces the challenge of mothers and neonates dying from preventable pregnancy-related complications. The free maternity policy (FMP), implemented in 2013 and expanded in 2017 (Linda Mama Policy (LMP)), sought to address the challenge. This study examines the quality of care (QoC) across the continuum of maternal care under the LMP in Kenya.

Methods

We conducted a convergent parallel mixed-methods study across multiple levels of the Kenyan health system, involving key informant interviews (KIIs) with national stakeholders (n=15), in-depth interviews (IDIs) with county officials and healthcare workers (HCWs) (n=21), exit interview survey with mothers (n=553) who utilised the LMP delivery services, and focus group discussions (FGDs) (n=9) with mothers who returned for postnatal visits (at 6, 10, and 14 weeks). Quantitative data was analysed descriptively, while qualitative data was analysed thematically. All the data were triangulated at the analysis and discussion stage using a framework approach guided by the QoC for Maternal and Newborns.

Results

The results showed that the expanded FMP enhanced maternal care access: geographical, financial, and service utilisation. However, the facilities and HCWs bore the brunt of the increased workload and burnout. There was a longer waiting time for the initial visit by the pregnant women because of the enhanced antenatal care (ANC) package of the LMP. The availability and standards of equipment, supplies, and infrastructure still posed challenges. Nurses were multitasking and motivated despite the human resources challenge. Mothers were happy to have received care information; however, there were challenges regarding respect and dignity they received (inadequate food, over-crowding, bed-sharing and lack of privacy), and they experienced physical, verbal, and emotional abuse and a lack of attention/care.

Conclusions

Addressing the negative aspects of QoC while strengthening the positives is necessary to achieve the UHC goals through better quality service for every woman.

Keywords: quality of care, maternal and childcare, maternal care, Linda Mama, free maternity policy, Kenya

For peer review only - http://bmjopen.bmj.com/site/about/guidelines.xhtml

STRENGTH AND LIMITATIONS OF THIS STUDY

- This is the first study to explore the optimal quality of care (QoC) across the continuum of maternal care (antenatal, perinatal, and postnatal care) under the expanded free maternity policy (FMP) in Kenya using the QoC for Maternal and Newborn - a monitoring framework for network countries.
- The use of a mixed methods approach in this study permitted complementarity, convergence and triangulation of the qualitative and quantitative data to deepen the policy description and analysis, hence attenuating the weaknesses of the singular methods.
- While the results may not be generalisable beyond the study county (area) because of the heterogeneity of the counties, this study identifies significant contextual factors that may have influenced the patterns of implementation and the findings, which are transferable (enhanced transferability) to other 47 counties in the counties and can be used to interpret the implications of the results in other settings.
- There could be many other unidentified QoC elements from this study, particularly other county-specific issues, but the findings could be considered the first step in exploring and compiling the existing knowledge about the global situation.
- This study could be particularly informative for policymakers as a guide to effective evidence-based interventions that can be adopted to strengthen the implementation of the FMP in the country.

For peer review only

INTRODUCTION

There are nearly 287,000 maternal deaths due to preventable pregnancy and childbirth-related complications happening globally (translating to almost 800 maternal deaths every day or one every two minutes) (1). Low-and-middle-income countries (LMIC) and low-income countries (LIC), especially those in sub-Saharan Africa (SSA), such as Kenya, are the most affected because of barriers to accessing maternal services (such as low quality of care (QoC), poor socio-economic conditions, poor infrastructure, and lack of well-trained healthcare professionals) (2-4). While Kenya's maternal and child health status has significantly improved in the last decade, the current maternal mortality ratio (MMR) of 530 deaths per 100,00 live births is significantly higher than the world average of 223 maternal deaths per 100,000 live births,(1) as is the neonatal mortality rate (NMR) of 21 deaths per 1,000 live births which is higher than the world average of 18 deaths per 1,000 (5, 6). Approximately 7,300 women still die every year making up 15% of all deaths among women of reproductive age, with both mothers and neonates dying from preventable pregnancy-related complications (7). One in 76 women in Kenya is at risk of dying from pregnancy complications (8).

As such, reducing and eliminating pregnancy-related mortality, ending preventable newborn and child mortality, and achieving Universal Health Coverage (UHC) remain crucial targets and priorities for realising the Sustainable Development Goals (SDGs) in Kenya. Various reforms in the health sector in Kenya have sought to achieve the above SDG targets by reducing catastrophic expenditure on maternity care and improving the quality of healthcare service delivery (9-13). One such reform was initiated in Kenya in June 2013, when the government launched a user fee waiver for maternity and primary health care (PHC) services (9). However, its implementation faced challenges of poor service delivery due to inadequate preparation before the implementation and a lack of adequate systems to verify the QoC provided and the reimbursement claims from the hospitals to the government (14).

Subsequently, to overcome these challenges, the country transitioned to a new expanded free maternity policy (FMP) in 2017 to provide access to maternal services to all pregnant women in an expanded network of providers, including private, faith-based, and all level 3–6 public institutions (15). The expanded FMP was called Linda Mama (LM) (Swahili for *"caring for the mother"*), and was managed through the National Hospital Insurance Fund (NHIF) to overcome challenges from the previous policy by enhancing administrative efficiency, ameliorating the reimbursement logistical challenges, creating a longer-term financing sustainability, and easing legal hurdles (16). Besides, it aimed to improve access to quality maternal and child services and reduce inequalities, thereby advancing the country's agenda of UHC (15, 17). The benefits package of the expanded policy captured both inpatient and outpatient services (including more antenatal services, delivery, postnatal care, and referrals of emergencies of pregnancy-related conditions and complications) for the mother and the newborn up to a year (18, 19).

Being part of the reform linked to the UHC agenda, there were three facets targeted for improvements: population, services and direct costs (20), envisaging that every person would have access to the entire range of quality health services and care they needed, whenever and wherever they needed them, without financial hardship (21, 22). The LM policy was mainly implemented to achieve the three facets. However, following the implementation of the two free maternity policies, researchers have focussed on mostly understanding two facets (population and cost), through studies focused on the policy's immediate and trend effect (23), its impacts on mortality and utilisation of

For peer review only - http://bmjopen.bmj.com/site/about/guidelines.xhtml

services (24-27), out of pocket expenditure (28), policy formulation and implementation elements (15-17), and the cost-benefit analysis (27). While there has been an attempt to look at the services, the quality of services and care aspects from both policies have not been conclusive and a gap remains. For instance, one study evaluated satisfaction with the delivery services under FMP (29). It showed that the mothers who benefited from the services were satisfied with different components such as communication by the healthcare workers (HCWs), staff availability in the wards and delivery rooms, and supplies availability, but were also unsatisfied with cleanliness, consultation time, and privacy in the wards. Another study evaluating the utilisation of the free maternity services implemented in 2013 among women living in Kibera slums in Nairobi showed that mothers positively perceived the distance to the facility and shorter waiting time, in addition to patients facing bad providers' attitudes (30). Yet, another study that evaluated disrespectful maternal care under the policy in Kisii and Kilifi counties showed that mothers experienced disrespectful maternal care throughout the maternity process, and it appeared even more significant among women who were poor, young, or had children with disabilities (31). All three studies on quality have focused on one aspect of quality: the outcome (from the patient perspective), leaving out other quality dimensions that researchers (32, 33) have discussed: structure, process, and outcome.

Therefore, the quality-of-service facet is yet to be fully explored. One study evaluated the characteristics associated with the QoC of the initial assessment for pregnant mothers, intrapartum, and postpartum and newborn care (continuum of care) not under the FMP but in the country context using service provision data and the finding was that a sustained focus on the QoC along the maternity care continuum was imperative for the mothers and their newborns and policymakers (in distributing resources to improve the areas of the continuum (34). Increasing service coverage alone is unlikely to produce better health outcomes without attention to the quality of care provided. The LM policy seeks to be a high-quality health intervention that optimises maternal care in the Kenyan context by consistently delivering and giving care that enhances or maintains maternal and neonatal outcomes, and that is valued and trusted by everyone since it responds to a changing population's needs (35). Maternal care under LM policy envisages enhancing the degree to which maternal services received by clients increase the likelihood of desired health outcomes consistent with current professional knowledge and are effective, safe, people-centred, timely, equitable, integrated and efficient (36). Therefore, exploring the optimal quality of maternal care and outcomes from the LM policy would be imperative. This study examines the QoC across the continuum of maternal care (antenatal, perinatal, and postnatal care) under the LM Policy in Kenya.

METHODS

Study Design

We utilised the convergent mixed methods design, specifically the parallel-database variant in this study (37) using qualitative and quantitative data that were collected and analysed in tandem and then compared and combined to better understand the QoC across the continuum of maternal care (antenatal, perinatal, and postnatal care) under the expanded FMP in Kenya.

Framework for analysis

As quality cannot be measured by itself (38), in this study, we conceptualised quality from the Donabedian perspective, broadly classifying quality as structure, process, and outcome dimensions (32, 33), which can be identified, measured, and attributed to healthcare. Akachi and Kruk (39) provide

For peer review only - http://bmjopen.bmj.com/site/about/guidelines.xhtml

more details on measuring changes in the QoC and bring attention to including user experience as a measure of outcomes in the quality assessment. With these two refined aspects, we broadly defined the structure indicators as pointers which are inputs to or characteristics of health; process indicators as gauges to either appropriate or inappropriate care in a targeted population which are 'consistent with current professional knowledge'; and outcome indicators as the measures of both improved or deteriorated health and attributed to medical care (38, 39). (See, Figure 1). Data collection methods and tools were designed to collect and examine all aspects of QoC across the continuum of maternal care (antenatal, perinatal, and postnatal care) under the free maternity policy in Kenya. Broadly, the analysis converges all the concepts using the QoC for Maternal and Newborn – a monitoring framework for network countries (40), which draws concepts from the earlier framework as proposed by the World Health Organisation (WHO) (41).

[INSERT FIGURE 1 HERE]

Study setting

The study was conducted across multiple levels within the Kenyan health system. The Kenyan health system is pluralistic in the provision and financing of services and is organised into six levels of care. Level 1 forms the community units overseen by community health workers (CHWs) whose role is providing promotive services (health education, treating minor ailments, and identifying cases that require referral to health facilities) (42), and both level 2 (dispensaries) and level 3 (health centres) provide primary healthcare services in addition to coordinating the community in their areas of jurisdiction. Level 4 and 5 offer curative services as county secondary referral facilities, with some being training centres, while level 6 are semi-autonomous tertiary facilities offering specialised care and serving as training institutions.

At the national level, we included the Ministry of Health (MoH), the NHIF, and development partner agencies involved in the expanded FMP. At the county level, this study was conducted in Kiambu County in Kenya. While this study is part of a larger study, Kiambu County was purposefully chosen because of its sociodemographic characteristics, health indicators, and population size (43-45). It is the second-most populous county in Kenya after Nairobi City County, with a population of 2,417,735: 49.1% male and 50.59% female (43), 26.9% of the population in Kiambu are female of reproductive age (15-49 Years) (44), 89.2% of births in the county happen in a health facility, 98.2% of births provided by a skilled provider, 67% of women aged 15-49 who had a live birth had 4+ antenatal visits, and 89% of women aged 15-49 had a postnatal check during the first two days after birth (5). While these statistics are slightly higher than the national average, they have not translated to quality care. Research has shown that primary care facilities with a low delivery volume have very low-quality delivery care, indicating crucial deficiencies in infrastructure and staffing, routine and emergency care practices, and referral systems (46). A majority of the facilities providing care in Kiambu are primary care facilities (70 tier 2 - dispensaries and tier 3 - health centres) with low volumes compared to the secondary facilities (13 tier 4 - hospitals and 1 tier 5 - inter-county facility) with a high volume (47). With the secondary care facilities receiving a higher population for care from the neighbouring counties and the locals (48), they are bound to be stretched beyond the limit, hence the potential for challenges in the QoC. Kiambu faces challenges with the interventions to address maternal health, such as referral systems that work, family planning, access to safe abortion services, availability of skilled health workers, and accessible health facilities (49-51) despite being cosmopolitan. With the

For peer review only - http://bmjopen.bmj.com/site/about/guidelines.xhtml

majority of the population in Kiambu being urban (43), which is facing overcrowding, there is a potential for the urban averages of maternal mortality to become either closer or worse than rural averages. Further, Kiambu County was purposively selected for this study because the county has been shown to pose higher trends in maternal mortality compared to other counties around Nairobi from the Central Region (52).

We purposefully selected three study facilities: a level 3 (considered a low volume – few numbers of clients), a level 4 (medium volume), and a level 5 (high volume). The facilities were chosen in consultation with the county team to provide nuanced, unique sub-counties dynamics given their richness in information and characteristics. (See Table 1).

Study population, sampling, and data collection

The study population used in this study were in four categories, as summarised in Table 1. We collected data between November 2018 and September 2019 through exit interviews (EIs), focus group discussions (FGDs), in-depth interviews (IDIs), and key informant interviews (KIIs).

The first group was staff from the MoH, NHIF, and development partners, who were purposefully selected based on their level of involvement in the expanded FMP. These respondents participated in KIIs with one researcher (BO), which were done in English, using KII guides developed to capture the experience of the formulation and implementation of the expanded FMP. All the KIIs (n=15) were conducted in Nairobi and were audiotaped following participants' consent using audio recorders. Each KII lasted between 45-60 minutes.

The second category included purposively selected respondents with knowledge of and experience in the implementation of the expanded FMP at the county (meso) level (including county and sub-county level officials from the County Department of Health); and the facility (micro) level (including facility in-charge, HCWs in charge of /offering maternal care/services, and other cadres of hospital workers) (Table 1). These respondents participated in IDI with one researcher (BO). The IDIs (n=21) were conducted in English using two semi-structured guides (each for the county and health facility participants) developed to capture the experience of implementing the expanded FMP. The construct validity of the two semi-structured guides was tested in the non-participating facility to check for ambiguity and flow of the questions. All the IDIs (save for one conducted at the place of convenience for the participant) were conducted at the participants' places of work and were audiotaped using audio recorders after obtaining their consent. Each IDI lasted between 30-60 minutes. The KIIs and IDIs were stopped when no new information, further dimensions, nuances, or insights were forthcoming (i.e. when meaning saturation was attained) (53). At this point, we noted that we had fully understood the issue under discussion.

The third group comprised of EIs with mothers who had delivered in the three hospitals and were discharged home. The sample size of the mothers was estimated at 553 using the formula proposed by Gorstein et al. (54). A detailed discussion of the sample criteria and dynamics across the three selected facilities has been published elsewhere (55). Four trained data collectors, supervised by one researcher (BO), conducted the EIs with the women. The design of the EI utilised a structured questionnaire, adapted from Dalinjong et al.,(56) to elucidate the sociodemographic information of the women, health and related services received at the facility (perception of the quality of maternal

For peer review only - http://bmjopen.bmj.com/site/about/guidelines.xhtml

care that the mothers received during delivery and antenatal care (ANC) care, experiences with the FM policy). The conduct of the EIs ensured that one researcher (BO) introduced the data collectors to the administration and the maternity department heads of the three facilities; then, each morning of the interview, they identified the mothers who had been discharged (using bed numbers) and were waiting to return home. With the number of mothers identified per day, we generated a random sample using Stat Trek's Random number generator (57), which was used to identify mothers for the EI. The mothers were then invited to participate in the study, and interviews were conducted until we reached the intended sample size. We took each mother through the information sheet, and only when they were comfortable participating did we give them the consent forms. One mother declined to participate (and we eliminated two entries at the analysis stage for lacking complete information).

The final category included FGD with nine groups of mothers (ranging from 5-12 mothers) purposively selected based on a common interest: mothers who had had a skilled delivery in a hospital setting and had come to the study sites for the 6-, 10-, or 14- week postnatal visits. One researcher (BO) conducted all 9 FGDs in Swahili (given the different levels of knowledge of the participants) using an FGD guide developed in reference to the gaps that had arisen from the EIs. The mothers in the FGD were recruited from the child welfare clinic of the three facilities when they brought their children for routine vaccination. The FGDs in each facility were organised with the help of a nurse from the maternity department. We engaged the mothers as the children received their vaccinations and asked if they would participate in the study. All the FGDs were conducted in a pre-booked room at the facilities and were audiotaped following participants' consent using audio recorders. Each FGD lasted between 45-90 minutes.

Table 1: Hospital characteristics and study population

	**Level 3 Hospital (Hospital A)**	**Level 4 Hospital (Hospital B)**	**Level 5 Hospital (Hospital C)**
**Hospital characteristics**			
Bed and cots capacity[a]	10	46	289
Number of staff[b]	35	115	262
Estimated annual deliveries[c]	1,076	5,635	9,152
Estimated annual outpatient care[c]	88,829	156,108	281,379
Estimated annual inpatient care[c]	764	7,223	14,205
**Hospital participants in the study**			
EIs	42	170	338
FGDs	3	3	3
IDIs	7	5	6
Facility level managers	*1*	*3*	*2*
Department in charges	*1*	*1*	*1*
Nursing officers	*4*	*0*	*1*
Accounting/ clerical officers	*1*	*1*	*2*
**County participants (IDI)**			**3**
Senior level managers			1
Middle-level manager			2
**National participants (KIIs)**			**15**
Ministry of Health officials			5

For peer review only

NHIF officials	3
Development partners	7
Notes: *Estimates for annual delivery, outpatient care and inpatient care were for the financial year July 2018 – June 2019; The outpatient total is an aggregate of both new and revisits.* ***EIs:*** *Exit Interviews;* ***FGDs:*** *Focus Group Discussions;* ***IDIs:*** *In-depth Interviews; and* ***KIIs:*** *Key Informant Interviews*	

Source: [a]Kenya Master Health Facility List(58), [b]In-depth interview with health facility in-charges of the individual facilities; [c]Kenya Health Information System (KHIS) for aggregate reporting(59).

Data management and analysis

Quantitative data from the EI was manually entered from the structured questionnaire into the Excel software by one researcher (BO), cleaned, checked for completeness, and then exported to STATA 15 for coding and analysis. The sociodemographic characteristics and the elements of quality were analysed descriptively using proportions.

All recorded FGDs were translated from Swahili to English, while the IDIs were transcribed verbatim in English. All transcripts were compared against their respective audio files by BO for transcription and translation accuracy. All the validated transcripts were imported into NVivo 12 for coding guided by the topic areas of quality of maternal healthcare. We used a framework approach to analyse the data guided by the QoC for Maternal and Newborn – a monitoring framework for network countries (40). This approach included systematic sifting, sorting, coding, and charting data into key issues and themes (60). One researcher (BO) familiarised himself with the data through immersion and repeatedly read and reread the transcripts. He then developed codes deductively from the conceptual framework and applied the codes to interpret segments in the transcripts that were important. The study team members (SK and SP) reviewed and discussed the initial coding framework, and any discrepancies were appropriately reconciled. The final coding framework was applied by (BO) to the data and later charted the data to allow the emergence of themes through comparisons and interpretations.

To enhance the interpretive rigour, we ensured credibility (also referred to as internal validity) through the convergence of evidence of the two methods utilised and triangulation (investigator, theoretical, and methodological) of data at the interpretive stage (61).

Ethics consideration

This study was part of a larger study (55) whose ethical approval was obtained from the University of Kent, the SSPSSR Students Ethics Committee and the AMREF Scientific and Ethics Review Unit in Kenya (Ref: AMREF – ESRC P537/2018). Further, we received written permission from the county government and all the hospitals to conduct the study. Before starting the interviews, we obtained written and oral informed consent from the potential participants. All the study participants were presented with information sheets on the conduct of the study, the researchers involved, the purpose of the study, the right to withdraw, and measures of confidentiality ensured before they gave their written informed consent. Participants were informed that data would be reported in an aggregated format, and anonymity would be ensured in storing and publishing the study's findings.

Patient and public involvement

For peer review only

Patients and/or the public were not involved in the design, or conduct, or reporting, or dissemination plans of this research. We intend to disseminate these research findings to the public through a summarised press article or brief, social media, and the websites of authors' institutions.

RESULTS

The results on the quality of maternal care in this study were presented using the WHO-proposed monitoring logic model from the perspective of the policy implementers and users. Results are presented in four broad domains: access to care (equitable and timely), provision of care (safe and effective), management and organisation, and care experience. A summary of the results is presented in

Table *2*.

Table 2: Summary of the quality of maternal care results

Domain	Sub-domain	Positive result	Negative result
**Element 1: Access to maternal care services under the expanded FMP (equitable and timely)**	***Minimised access barriers (cultural, financial, geographic)***	The expanded FMP enhanced maternal care access elements (geographical, financial, or utilisation of services).	However, the facilities and HCWs were bearing the brunt of the burden of increased numbers of mothers seeking LM care (workload and burnout)
			There was an altered perception among women, leading to a preference for higher-level facilities.
	Additional maternal determinants of care and the timeliness of care	The distance to the hospital was perceived as normal (okay for the patients) and the preferred choice of transport to the facility was public transport	There was a longer waiting time for the initial visit by the pregnant women due to the enhanced ANC package of the expanded FMP.
		All the three hospitals had a proper waiting area.	
		There was a positive perception about the time to seek care and the waiting time.	
	Provider availability		There were problems of struggling to employ specialists and other HCWs staffing challenges.
**Element 2: Provision of care (safe and effective)**	***Functional referral system***		Fewer women were being referred, but they had a better perception of services received during referral.
			The facilities' lack of equipment, theatre, NBU and blood were the main reasons for referrals.
	Safety	Because of the policy, the facilities were managing complications better	HCWs were reducing the time they allocate per mother.

For peer review only

**Element 3: Management and organisation**	***Availability of essential physical resources***	The policy has improved the availability and standards of equipment and supplies.	Despite progress, some infrastructure, commodities, and supplies are still a challenge to some facilities.
		The facilities had improved infrastructure due to LM.	
		Enhanced facility resources and facility characteristics.	
	Competent and motivated staff	Mothers have a strong positive perception of healthcare delivery characteristics by the HCWs.	There were some causes of demotivation and dissatisfaction among HCWs.
		Nurses are multitasking and handling many roles amidst the challenge of human resources.	
		HCWs are adequately motivated to work despite the challenges.	
		HCWs' source of motivation was more than just money.	
	Monitoring and continuous quality improvement	Nurses monitor the quality of care provided through partographing and charting labour progress, though they face challenges.	
**Element 4: Experience of care**	***Effective communication with the patients***	Mothers perceived and experienced the positive interpersonal qualities of the HCWs.	Inadequate preparation for birth by the HCWs.
		Mothers were happy to have received information about emergency/ procedures and training on breastfeeding, family planning, and baby care.	The lack of proper 11ducationn and communication on expectations.
	Respect and dignity		Food was perceived as inadequate in some hospitals.
			Overcrowding and bed-sharing led to a lack of privacy (congestion) and a lack of essential equipment and supplies, altering the QoC.
	Emotional support		Women were experiencing physical, verbal, and emotional abuse.
			Some mothers experienced a lack of attention/care, negligence, and unhygienic practices from the HCWs and support staff.

Element 1: Access to maternal care services under the expanded FMP (equitable and timely)

Minimised access barriers (cultural, financial, geographic)

For peer review only

The expanded FMP enhanced maternal care access elements (geographical, financial, or utilisation of services). For instance, due to the policy, there was an increase in the utilisation of maternal services (delivery and ANC). Findings from Eis showed that most mothers across the study sites (99.09%, n=545) visited a hospital for maternal health services during their pregnancy (Appendix 1). Further, IDI showed that more mothers (than previously) were confident in seeking skilled services rather than remaining at home.

> *'…mothers who could not come, now they are coming. And there is also a change in the number of deliveries we used to have before and now' –* ***(R009, Nursing officer).***

Equally, the respondents noted that with the enhanced identification strategies for the mothers, the expanded FMP saw increased access to services among vulnerable populations such as street children, orphans, and adolescents. Besides, they averred that the policy enhanced equity (as those in the rural and urban areas received uniform services), and the women had better financial access to the services (free services).

However, the facilities and HCWs were bearing the burden of increased numbers of mothers seeking LM care. As noted by most respondents, facilities were bearing the brunt of the increased number of mothers due to LM, which resulted in space shortages and increased workload. The workload was further exacerbated by the nature of work in the public facilities where the HCWs had no choice but to serve the mothers and meet the required utilisation targets. However, the facilities were working way beyond their abilities to manage the workload, and it resulted in HCWs experiencing some burnout:

> *'We work extra hours…you will find each care provider is serving more than they should, so the issue of burnout is also coming up' –* ***(R019, Facility Level Manager)***

There was an altered perception among women, leading to a preference for higher-level facilities. There was an increased workload in higher-level facilities caused by the mother's perception of there being specialist health care professionals that the lower-level dispensaries or community centres lack. As a result, the women believed that higher-level facilities had a higher chance of dealing with complications than the lower-level hospitals:

> *'…sometimes you ask them, "Why have you decided to come here?" "Because here, people who will attend to me are qualified."…But they say outside there, anybody can attend you.' –* ***(R014, Nursing officer).***

Additional maternal determinants of care and the timeliness of care

There was a positive perception about the time taken to seek care and the waiting time. A majority of the women visited a public facility (92%); and had a positive perception about the time taken to the facility and the distance to the hospital. Women who visited hospitals A (45.24%), B (51.18%), C (46.75%), and overall (48.00%)) noted that they took 30 minutes to 1 hour to seek delivery services and they perceived the time to be short (Appendix 1).

A majority (61.64%) perceived the distance to the hospital was normal (okay for the patients), and the preferred choice of transport to the facility was public transport (40.73%) (Appendix 1). Also, all three hospitals had a proper waiting area. Most of the women were happy with the time the facilities were

For peer review only - http://bmjopen.bmj.com/site/about/guidelines.xhtml

being opened and perceived the waiting time before being attended to as short (43.09%) (Appendix 1).

There was a longer waiting time for the initial visit by the pregnant women due to the enhanced ANC package of the expanded FMP. The initial ANC profile included blood tests (for haemoglobin levels, blood group, rhesus, serology), Screening for tuberculosis, HIV testing and counselling, urinalysis, preventive services (such as deworming, intermittent preventive treatment for malaria, iron and folate supplementation) and prevention of mother to child transmission. All these were done at the same laboratory as other patients in the hospitals; hence, they had to wait for longer to get results:

> *'…for the first visit [they] will report here at 8:00[am] and…get out of this place as late as 3:00[pm]…because when they come…if it's lab everybody is there, the people who are coming for outpatient services are queuing there [too]…the rebate for the first visit [ANC]…covers up a lot'* – ***(R002, Clerical Officer).***

Provider availability

There were problems of struggling to employ specialists and other HCWs staffing challenges. The facility in-charges noted that they had a challenge of hiring specialist nurses to take care of the growing numbers, which had been exacerbated by the lack of specialised units in some facilities:

> *'…we could not set up a neonatal ward [for lack of] a neonatal nurse...[yet] we get so many babies, and with that influx, we could still get some babies...'* – ***(R020, Facility level manager)***

One facility in-charge noted that while the facilities had installed an ultrasound machine to meet the needs of the pregnant mothers, there was a gap in trying to identify the person to operate it and sustainably pay the staff.

The staffing challenge, particularly in the lower-level facilities, was hard to deal with because of the rules of staffing where, despite the high number of mothers, the number of staff cannot go beyond a certain number:

> *'…I think it's not because of Linda Mama, I think it's because of how it has been, we have been a level 3, although they said they would add us people. But you see they cannot exceed the number of staff in a level 3. If it were a level 4, they would increase.'* – ***(R007, Department in-charge)***

Element 2: Provision of care (safe and effective)

Functional referral system

Fewer women are being referred, but they have a better perception of services received during referral. While referral of emergency cases is essential in preventing complications, results from EI showed that only 10.73% (n=59) of all the women interviewed, had been referred for additional care. Most had been referred from level 3 facilities (n=26), using an ambulance (n=22) or public means (n=15), and were mainly accompanied by their husbands (n=27), relatives (n=23) or health workers (n=21) either as an individual or both at the same time (Appendix 2). A majority of the mothers' companions had knowledge of emergency management (n=47), were allowed to stay in the hospitals (n=33) and were warmly received at the hospitals (n=19) during the referral (Appendix 2).

For peer review only - http://bmjopen.bmj.com/site/about/guidelines.xhtml

The women in the FGDs perceived that the maternal services provided by the mothers had improved because of the LM policy, leading to a reduction in referrals:

> *'**R3:** I can say the services are good because nowadays we don't run to [referral hospital] the way we used to. So, this hospital has been good, it has been helpful to us.'* – ***(Woman in FGD003).***

The lack of equipment was the main reason for referral, and most women sought their own referral means from the hospital. From the EI, the referred mothers noted that lack of equipment, theatre, NBU and blood (n=16) were the leading cause of referral, followed by foetal distress (n=7) (Appendix 3). Whereas HCWs indicated that the county and facilities provide some form of referral transport for mothers, the referred mothers reported seeking their transport means for referral. These mothers perceived this to be dangerous for their health and safety and expensive, especially in unplanned emergencies.

> *'**R5:** …they [health workers] told me there's no vehicle, and they insist, "Look for a vehicle quickly so she can be referred" …now to do it fast and you don't have money…I really suffered; R8:…if a mother delivers now, [and]…is going to [a referral facility] and you know the road there is not good and someone has been stitched up down there [episiotomy]…when going there the stitches might be undone…'* – ***(Women in FGD009)***

Safety

Because of the policy, the facilities were managing complications better. HCWs and hospital administrations acknowledged that the policy improved the facilities' management of complications. The policy objectives incentivised them:

> *'…for example, she [patient] comes up with a chronic infection, which means the administration will spend more money buying an expensive drug for her. But you see, the moment she comes on time, early enough, she knows, "I went to the clinic, I was told I cannot deliver normally." She will come here on time. So, she will be told, "The moment you have reached 40 weeks, go to the hospital," she will be here. We do her C-section very safely; it is very simple she goes home. NBU decongested here…also the chorioamnionitis are no longer there.'* – ***(R012, Department In-charge)***

HCWs were reducing the time they allocate per mother. Given the workload that the HCWs were facing, they were reducing the time they allocated to providing each mother with care, and even some lower-level facilities were sending away mothers for they had higher numbers of patients:

> *'Owing to the fact that the patient numbers are higher than the health workers, the burden on the health worker is greater. Meaning the time allocated per patient is less than required'* – ***(R005, Facility Level Manager)***

Element 3: Management and organisation

Availability of essential physical resources

The policy has improved the availability and standards of equipment and supplies. With the help of reimbursements from the free policy, the facilities reported to have had improvements in the availability of supplies and medical equipment. In fact, the facilities have kept reordering supplies to keep up with the demand:

For peer review only - http://bmjopen.bmj.com/site/about/guidelines.xhtml

'…we've not actually gone out of stock. But you find we have to keep reordering because the demand is more.' – ***(R020, Facility Level Manager)***

Further, it was shown that with the availability of equipment and supplies, the HCWs did not have to utilise substandard care or equipment. For instance, one facility has shown how they had now departmentalised the sterilisation process of the equipment rather than using the hospital steriliser. With this came the availability of delivery packs, and they are no longer using ordinary blades as before:

'…we have so many like delivery packs which we used not to have. Sometimes we used to…. Use a blade instead of a delivery pack or the scissors because these things were not there…. There are people who are employed to cater for washing those things…and take…them [to] utility for preparation for next use.' – ***(R014, Nursing Officer)***

The facilities had improved infrastructure due to LM. Some facilities had used the reimbursements from the policy to improve infrastructure (such as theatre and ultrasound areas). Additionally, some were expanding their buildings to reduce congestion. For instance, one facility had been able to complete a section of an incomplete building and transfer mothers to it from the congested postnatal ward:

'…when our mothers are many in this maternity [in facility C]…those without complications or those who had delivered yesterday, we transfer them to that department, so there is that decongestion. And we have another building there, the reproductive health, it is only that it is not yet over [complete]…but now the patients who are being attended…were transferred to that department and…we got the extension.' – ***(R014, Nursing Officer)***

Other facilities even renovated older buildings that were no longer in use and converted them into maternity clinics to ease congestion. For instance, in facility B, one building constructed five years ago to be a mortuary that was only used to store patients' records has now been refurbished and used as an outpatient clinic. The downside was that the mothers had a negative attitude towards it as they believed it was still a mortuary.

Additionally, the policy reimbursements helped facilities meet their essential services, which were critical in easing the burden of work. As noted by HCWs, they could incentivise mothers by using elements such as transport that would help improve quality. However, with more patients came more workload:

'…sometimes that money will help to fuel the vehicle and…to maintain the ambulance…[and] sometimes it can support…staffs to go for seminars and…to conduct those in-reach…and also outreach services' – ***(R008, Nursing Officer)***

'….in a way it's a pusher to more quality service to the client…because you want…to attract more…because the more, the better. But…that also has brought the issue of us bursting through the seams.' – ***(R019, Facility Level Manager)***

Enhanced facility resources and facility characteristics. The women in the EI ascertained that there was an enhancement of the resources in the facilities due to the policy. The facilities were shown to have adequate waiting and examination rooms (51.60%); adequate hand washing facilities (91.82%);

For peer review only - http://bmjopen.bmj.com/site/about/guidelines.xhtml

adequate bathing facilities (67.46%); adequate toilet facilities (71.45%); well-suited equipment for detecting women's problems (90.91%); had an adequate number of staff (76.37%) who are well suited to treat women (96.55%); and had an overall clean environment (93.45%) (Appendix 4). However, the mothers showed some concern about the adequacy of the facility providing clean drinking water, as indicated by 46.18% of mothers (Appendix 4).

Despite progress, some infrastructure, commodities, and supplies are still a challenge to some facilities. Some respondents noted that some facilities still have inadequate medical equipment (such as ultrasounds), space and supplies. The lack of these basic elements, such as a basic laboratory, was demotivating the women from using the services in the hospital and preventing HCWs from completely following up with the mothers as they would have wished to.

> *'…we don't have a very vibrant laboratory…as a clinician, I believe you want the patient tested, drugs availed, that patient will not come back to you after two days [said with wry humour]. You can give them a prescription, and they tell you they bought half a dose because they didn't have money, now, how will you help them? You see, it demotivates…… Yes. Even the ultrasound, the scans, we don't have the scans, so they have to do the scans outside [the facility]…About the [ward] it's not an ideal labour ward. We don't even have an ideal resuscitaire, you know, the improvised one?...you have to be extra cautious not to shake that thing, so the heater falls on the baby. Imagine, you have three mothers delivering, and you deliver as you put there...In the process you can burn those babies as you go to pick the other one...so, you have to be extra cautious... Even IPC [infection prevention and control] becomes an issue.'* – ***(R018, Facility level manager)***

The noted challenge regarding the supplies was that the county government was focusing on improving infrastructure, which was visible to the women, and perceived it as a better investment, rather than supplies and medication. The HCWs posited that the medication posed the biggest headache, whose potential cause was the drug ordering protocol. The facilities had to wait for a certain number of days before receiving top up for their orders:

> *'…there is a protocol…because like our drugs are ordered through KEMSA for a certain period, by any chance those drugs are not enough…they get finished before that period, we have to wait for the other order. But usually, in a hospital like ours [high-level facility], sometimes we are given extra money like miscellaneous where you can purchase emergency. But even when you purchase emergency like drugs, we are able to purchase a start dose or a prophylaxis, for continuity, you find now you have to involve maybe the patient.'* – ***(R020, Facility level manager)***

Competent and motivated staff

Mothers have a strong positive perception of healthcare delivery characteristics by the HCWs. A majority of the mothers in the EI had a positive perception of the healthcare delivery characteristics. For instance, 95.27% perceived that the staff examined pregnant and postpartum women well; 95.45% noted that the staff were very capable of finding out what is wrong with mothers; 59.64% noted that staff prescribed drugs that are needed and that the drugs supplied by the health facility were good (58.37%) and the mothers could obtain the drugs from health facility easily (67.27%) (Appendix 4). In addition, 71,27% perceived that they received adequate Information on danger signs of delivery and postpartum (Appendix 4). Interestingly, 79.82% perceived that the facility provided privacy during vaginal examination and delivery and 84.70% believed that the procedure they received during ANC and delivery felt very much necessary (Appendix 4).

For peer review only - http://bmjopen.bmj.com/site/about/guidelines.xhtml

Nurses multitask and handle many roles amidst human resource challenges. Nurses, especially in the lower-level facility, were shown to go over and above in their work. They covered night and day shifts, handling other hospital consultations at night, and still accompanied the pregnant women during referrals. Despite the tasks being their roles, the constrained number of nurses was making the staff rotation allocation challenging, and hence, they had to multitask amidst the challenges.

Besides, because of the challenges of the increased workload from the LM policy, even the nurses in charge of both department and hospital administrations were forced to do the actual hands-on nursing practice rather than just stay in the office doing administrative work to ensure that the services are timely provided. Also, the nurses in the maternity wing asked for help from other departments when the work became overwhelming:

> *'…there is also the issue of shortage. Like today, we are so many, but at least we have covered all areas. But other times we report like three people, so…we have to work here and go to that place [to work in the wards]'* – ***(R007, Department in-charge)***

> *'We call help from other departments when it's so much.'* – ***(R001, Department in-charge)***

HCWs are adequately motivated to work despite the challenges. The HCWs reported being motivated to work more because they perceived that the more efforts that they put into providing service, the more the LM reimbursement funds the facility would make, which would subsequently translate to better services and additional hands (through locum nurses):

> *'…the policy of Linda mama has motivated the staff. At least we know that if you put more effort, there will be more funds on the facility, we will get more commodities, we will be compensated for escort [referral] and lunch…it will be more comfortable for us.'* – ***(R003, Nursing Officer)***

The hospital in-charges noted that despite the high workload, they feel that the HCWs are motivated and that they presented a perfect picture during supervision. For example, they noted that some were even comfortable running the wards alone without the support of other nurses and forfeiting their lunch time:

> *'…they go overboard [HCWs]…you would find two nurses on night duty, conducting 15-17 deliveries…alone. And finding this nurse has to monitor this mother from admission, delivery and postnatal and also the baby, you find they go overboard…like our nurses in maternity, they would not even break for lunch. They would wait until now the shift is over.'* – ***(R020, Facility level manager)***

Some mothers reported that the HCWs served them even when it was not their working shifts, which signified dedication to work:

> ***'R4:*** *I came here at 2:00 pm, and I got a doctor who was on the morning shift and the other one was changing. So, I told him to serve me, I wanted to deliver. He dressed in a hurry and came to help me.'* – ***(Woman in FGD003)***

In fact, the other cadre of HCWs, such as department clerical officers, noted that amidst the challenges, they worked beyond the stipulated hours either to support the provision of LM services or to batch the claims and ensure that the hospitals receive timely reimbursement. However, they faced a challenge with inadequate and insufficient infrastructure (such as computers to ease work)

For peer review only - http://bmjopen.bmj.com/site/about/guidelines.xhtml

and salaries. However, despite the challenges, they perceived that they knew how to plan their days and work.

HCWs' source of motivation was more than just money. Some of the factors that the HCWs indicated as a source of motivation, rather than monetary values, were the kind acts and listening ear of the county administration and facility in-charges. For instance, in one facility, the department in charge felt that the administration provided them with a listening ear and acted on their grievances, including renovating the theatre and expanding the admission area. Others also felt that it resulted in the provision of adequate equipment and supplies to the facilities without having to improvise the old equipment:

> *'…at least we are listened to when we at least raise something…at least we get better service operating because of that. I mean theatre…was moved from here the squeezed area to that place, and then there wasn't bed, it was brought.'* – ***(R001, Department In-Charge)***

> *'…once in a while, we call them, have breakfast meetings with them, listen to their issues, discuss with them'* – ***(R016, County Senior Level Manager)***

The other source of motivation was that HCWs were happy when their burden of work was eased and department in-charges were doing so by employing additional people on locums, providing training opportunities, and recognising them for risking their lives at night during referrals to other facilities. Further, the nurses felt that they were involved in decision making and they perceived that it gave them a voice to raise an opinion on how the work needs to be done:

> *'So that one I see at least they could have involved us the people on the ground'* – ***(R014, Nursing Officer)***

There were some causes of demotivation and dissatisfaction among HCWs. For instance, HCWs noted that they felt inadequately remunerated despite the increased workload from the policy. With the workload, others felt that they had to multitask (for instance, handle referrals at all hours of the night and still had to come back to the facility after referral to carry out their duties which were waiting for them, and which they felt they were not adequately motivated for):

> *'We are underpaid, yeah let me say that without fear because we do a lot of work. You see like the time you came into the office; I was so buried there. I have been sitting there since 7:30 am'* – ***(R011, Clerical Officer)***

Similarly, the in-charges of the maternity departments, who were also HCWs, noted that the lack of timely reimbursements from the LM policy demotivated them. With such delays, the in-charges were having a strained working relationship with the hospital suppliers and even banks:

> *'You are doing your services, and you are claiming, but you are not getting the benefit of your work, so it renders even demoralising the people [HCWs in] the maternity…the same might demoralise even the suppliers who do supply us with the goods…some of them do cut off deals with dealing with the facility. Because we do pay them very late, and sometimes, they attract interest in their banks.'* – ***(R006, Nursing Officer)***

Monitoring and continuous quality improvement

For peer review only - http://bmjopen.bmj.com/site/about/guidelines.xhtml

Nurses monitor the QoC provided through partographing and charting labour progress, though they face challenges. The nurses showed awareness of proper documentation of labour progress using a partograph to enhance quality care. However, they noted that they sometimes faced additional scenarios (presentations/ conditions from the patients, e.g., those from referrals or mothers who came in at the second phases of labour and delivered within a few minutes of admission) that they did not know how to document.

> *'although once in a while a file maybe there is a problem, but they try…..because you know a partograph is very important…I know maybe you have found challenges in those partographs when you were going through.'* – ***(R007, Department In-Charge)*****.**

Despite the challenges, the nursing in-charges and facility managers were organising additional education for staff on the monitoring processes for pregnant women. The university students, who were posted to the facilities for training, or even nurses who had had more recent training, were tasked to provide additional education to the nurses as they had more recent knowledge.

Element 4: Experience of care

Overall, a majority of the mothers (84.2%) from the EI were completely satisfied with the services they received (hospital A (85.1%), B (80.9%) and C (85.2%) were completely satisfied with the services provided). A higher proportion of mothers in hospital C (74.4%) than B (66.7%) and A (74.1%), would consider future delivery in the same health facility (Figure 2).

[INSERT FIGURE 2 HERE]

Effective communication with the patients

Mothers perceived and experienced the positive interpersonal qualities of the HCWs. A majority of the mothers in the EI had a positive perception (agreeing and completely agreeing) about the HCWs as being very open (94.34%); compassionate (90.58%); respectful (95.46%); devoted adequate time to the mothers (94.18%); and are very honest (92.00%) (Appendix 4). Some mothers noted that the HCWs were empathetic, friendly, and reassuring. They appreciated the additional good treatment and sacrifices the HCWs made, such as warming food and additional support (such as bathing the baby and changing bedsheets and stained beddings) following the exhausting birth experience.

Some mothers appreciated being given priority in treatment, especially during emergencies by the doctors. In such circumstances, the firmness and decisiveness of the nurses were also perceived positively as being intent on preserving the lives of both the mother and baby. One mother was particularly impressed with the doctors who called for assistance in emergency scenarios when they were not able to handle them at the time:

> ***'R1:*** *when I came once I got a certain doctor and I think there was an emergency, and I was forced to wait but I did not take offence because…he called another doctor who came here and I saw they have experience because they just serve you.'* – ***(Woman in FGD003).***

Mothers were happy to have received information about emergency/ procedures, and training on breastfeeding, family planning, and baby care. Some mothers highlighted that since some doctors

For peer review only - http://bmjopen.bmj.com/site/about/guidelines.xhtml

explained to them the medical procedures they were to undergo, they were able to relieve some anxiety around birth especially:

> '**R2:** *The doctor was good, he told me how it [procedure] would be done, and I was good.'* – ***(Woman in FGD004).***

The nurses supported the mothers during breastfeeding, taught them how to breastfeed and even encouraged those with difficulties. Some hospitals even went further by demonstrating to the mothers through *YouTube* videos, the procedure of breastfeeding, which they (mothers) perceived as very useful and helpful. The facility in-charges acknowledged that they trained and empowered the nurses with breastfeeding knowledge to ensure that they in turn train the mothers:

> *'And once this nurse trains in the breastfeeding, she'll go back, we make it as a duty for her to be educating the mother on those…on breastfeeding'* – **(R020, Facility Level Manager).**

Besides breastfeeding, the mothers acknowledged being taught about family planning, how to wash the baby's cord, and what to do if the baby faced some complications, which they considered reassuring.

Inadequate preparation for birth by the HCWs. Some HCWs were perceived as not being well prepared to handle the birth of the baby, given that they never had the birth equipment readily laid or that some materials and supplies were not readily available. This ultimately resulted in birth complications such as amniotic fluid aspiration.

The lack of proper education and communication on expectations. Some mothers felt there was no clear communication on the immediate care after delivery, which created a knowledge gap and potentially made mothers make mistakes with medications that resulted in medical emergencies. For instance, one mother indicated:

> '**R6:** *For my child there was a time I put the Hexi-cord [cord cleaning medication] on their nose. I did not know; I asked my husband to pass me the medicine at night thinking it was a nose drip. So, we thought that was it and we administered to him, we were forced to bring the baby here at night.'* – ***(Woman in FGD001).***

Some HCWs were perceived as not being reassuring and unable to provide mothers with the expected reassurance:

> '**R12:** *"We have examined you; the baby is not close." You know, sometimes you feel the baby is close, and when it's time to deliver, many doctors and nurses came and told me, "Why are you disturbing us, you are standing on the floor. Climb the bed." I could not climb. They said, "We are referring you to [a referral hospital]." Now I said, oh my god what will I do? At that time, they started to insult me and told me, "Come here, you are going to deliver in the ward."'* – ***(Woman in FGD009).***

Respect and dignity

Food was perceived as inadequate in some hospitals. Some mothers revealed that despite having a good birth experience in the labour ward and not paying anything for the delivery, the food provided, particularly by the support staff post-delivery, was inadequate, untimely, and unwholesome. Some

For peer review only - http://bmjopen.bmj.com/site/about/guidelines.xhtml

mothers in some facilities highlighted that appetence for food could sometimes last for a whole night post-delivery, and, thus they resorted to having their relatives and family bring them food.

> *‘**R2:** I didn’t pay anything, though their food is too little for a pregnant woman. It’s true, it’s too little, a mother has delivered, that food…and then they serve it very early, when it reaches 9pm you are hungry again…Yes, I had to call home [for food] because I felt weak. R7: There was a day I stayed here without food the whole night. I wasn’t given.’ – **(Women in FGD009).***

However, the administration revealed that the instance of food inadequacy may have been caused by the support staff who, despite the facility planning for adequate food for the whole hospital patients, may have rationed the food further. Despite the inadequacy of food, some mothers acknowledged that the food was actually good:

> *‘**R3:** Yeah, it was good, I ate good things, and even the bathroom was clean. The services there are good.’ – **(woman in FGD003)***

There was overcrowding and bed-sharing, leading to a lack of privacy (congestion) and essential equipment and supplies, altering the QoC. Congestion in the maternity department because of the expanded FMP was a cross-cutting theme, especially in the higher-level facilities. The lower-level facilities equally faced an increase in the number of mothers, particularly for ANC and delivery, but the mothers did not share beds:

> *‘**R3:** but the problem I found here is congestion…. The first three hours [following CS] … I slept on a bed alone, after three hours we were two people on the bed. And from there the room we were taken too we would sleep four people with children, six people like that in one bed…. Because I left there with a back problem because I cannot sleep, you are forced to sit, you sit for the child to sleep.’ – **(woman in FGD005).***

Nonetheless, the hospitals gave bed priority to mothers who had had a caesarean section (CS) over normal delivery and allowed them to sleep on the bed alone in space-permitting instances in addition to having a special monitoring room. In contrast, mothers who had given birth normally were forced to share beds with other mothers or sleep on the floor, with only the babies sleeping on the beds. The congestion in the public facilities forced the mothers to seek care elsewhere.

Also, despite there being the expanded FMP, the lack of basic essential equipment and space was also noted to be a key driver to poor QoC even in maternal and child health clinics for PNC:

> *‘Go to MCH [maternal and child health clinic]… and see how babies are weighed naked outside, in this harsh weather. It is at times very cold in the morning but what do we do, we have to weigh them….but we are glad that we are still able to offer services’ – **(R018, Facility Level Manager).***

Emotional support

Women experienced physical, verbal, and emotional abuse. Some mothers experienced both physical and verbal abuse from HCWs and support staff. The abuse was exacerbated by the lack of clarity in communication with HCWs. For instance, one woman reported that the nurses had slapped her for being stubborn and uncooperative during birth, another woman mentioned that the nurse had tried to suture her episiotomy without using anaesthesia, and still another received abuse in return from either support staff or HCWs for requesting support:

For peer review only - http://bmjopen.bmj.com/site/about/guidelines.xhtml

*'**R6:** I was slapped here…. For being stubborn; R3: you see someone is still in pain, they do not inject you with anaesthesia and they want to stitch you. Things like that are not good, this is also a human being, and they still feel pain. R5: I saw someone who had gone through a CS, and they told the nurse, they wanted to rise up, you know there is pain while rising up…but I saw her telling that nurse to help her get up, I saw [heard] the nurse insult her and I did not like that' – **(Women in FGD001)***

Equally important was one mother's testimony showing how she was wheeled to the theatre in a rather uncaring manner that lacked dignity:

*'**R5:** What I saw, what he did to me, when I was experiencing labour pains, I was told to go to theatre, and I told him I cannot walk. He pushed me like a cart up to the theatre. I told him I could not walk; he pushed me like a lorry.' – **(Woman in FGD008)***

Some mothers experienced a lack of attention/care, negligence and unhygienic practices from the HCWs and support staff. For instance, in one case, a doctor was shown to have forgotten to remove cotton wool used in packing blood after delivery:

*'**R6:** Like in my case they did not remove that thing [cotton wool] and then I went home with it.' – **(Woman in FGD002)***

Additionally, some mothers perceived that some HCWs were not giving them and their babies proper attention while attending to them and they felt ignored. For instance, one respondent whose baby required medical oxygen felt a lack of support:

*'**R6:** the baby came out fine. But I saw that by the time the nurse received him, he wasn't breathing well and then the nurses did not care because when I woke up after six hours I had to go look after my baby, when the oxygen came out, I would put it back, I changed everything. So, this time round I did not like them.' – **(Woman in FGD006)***

Some mothers were subjected to unhygienic practices by some HCWs, including being examined on an unclean bed previously used by another patient without wiping or being left unattended for long:

*'**R4:** Another thing that I didn't like there, you are examined on a bed that someone else had been examined on and it is damp. It wasn't good. Like for me I was examined on a bed that had some liquid substance; **R9:** I delivered at [a referral hospital]; I didn't like their services at all. Because when I delivered, I was cut down there [episiotomy] and the doctor left me for 30 minutes. On coming back, he stitched me with all that dirt, so I was not happy at all with their service.' – **(Women in FGD009,).***

Some support staff also exacerbated the unhygienic practices of the mothers. For instance, one mother noted:

*'**R6:** when I delivered here, I was asleep, when I woke up around 6.30. I found they [support staff] had opened windows as they wanted to clean. If you had put your bag on the floor, they ask you to pick it up and put it in bed and that bed is where you place the baby, and the ground is dirty.' – **(Woman in FGD001).***

DISCUSSION

For peer review only - http://bmjopen.bmj.com/site/about/guidelines.xhtml

To our knowledge, this is the first study to examine the QoC across the continuum of maternal care (antenatal, perinatal, and postnatal care) under the expanded FMP or LM policy in Kenya.

Our findings show that the LM policy has reduced geographical access barriers by harnessing more private sector and faith-based facilities to enhance service provision. Furthermore, it has eliminated financial access barriers through the incentives of free maternal care and increased utilisation of maternal services (more mothers seek SBA; hence, reduced home deliveries). These findings align with results from systematic reviews of maternal services under different free maternity policies, which showed increased maternal (ANC and delivery) services after removing user fees (62, 63). Dossou et al. (64) also showed a systematic increase in CS services after implementing the CS policy in Benin because of utilisation incentives. However, the reviews showed that the utilisation patterns under free policies were marred by geographical and temporal fluctuations in use, which differs from our study.

Further, despite the policy enhancing access, the facilities were using additional approaches and incentives to attract mothers, leading to a difference in perception of the services provided. The finding on factors leading to the choice of the delivery place is not new, as other authors have highlighted the difference in the preference for private or public facilities thus influencing perception (65-67). In fact, in a recent FGD with women in Nairobi's informal settlements in Kenya, exploring their experiences of the quality of maternity care under LM, Oluoch-Aridi et al. (68) present the facilitators and barriers to choosing health facilities, which are all similar to the findings of this study. Interestingly, the choice of delivery site was influenced by several factors that are not necessarily related to LM, such as personal choice, previous experience or treatment, and access, as shown in other studies (4, 69) or health system factors (70). This highlights a key gap because it raises the question of whether LM has influenced the choice of hospital for delivery. Escamilla et al. (71) showed that the need for free services in Kenya had influenced women to bypass nearer facilities for farther private facilities that offered free care, which is similar to the findings from Sierra Leone by Fleming et al. (72).

Interestingly, our finding shows that while there was an increase in the utilisation of free maternal services, the facilities and HCWs bore the burden of providing service to more mothers seeking LM care. This finding aligns with other authors' findings, which have shown a significant increase in the utilisation of maternal services following the implementation of the free policy in Kenya (14, 73, 74), which was attributable to the removal of cost barriers to women (75). Nonetheless, our study goes further to highlight that despite bearing the burden, the facilities and the HCWs were shown to be working beyond their capacity to provide care to the extent that the HCWs ended up experiencing burnout. The unintended consequence of the increased burden on the HCWs could be explained by the fact that the implemented policy did not translate to an equal investment in an increased number of HCWs, hence the burden. Previous studies have shown that the perennial lack of human resources has always been a problem in Kenya. For instance, Miseda et al. (76) reveal that out of the 138,266 HCWs required to fit the MoH Norms and Standards Guidelines for service delivery, only 31,412 are employed at the public sector, private facilities, and faith-based organisations (FBOs).

It was also shown that the HCWs went beyond their strengths to serve the increased number of mothers well as a way to maximise reimbursements from the LM policy, but this could cause burnout

For peer review only - http://bmjopen.bmj.com/site/about/guidelines.xhtml

if not followed by an increased workforce, thus leading to poor QoC. Two meta-analysis studies have shown that HCWs burnout could lead to the provision of poor QoC (77, 78). HCWs are motivated by what Franco et al. (79) deriving from Herzberg et al. (80) refer to as 'hygiene factors' (determining HCWs dissatisfaction) in this case the interpersonal relationship with the county and the administration, and 'motivating factor' (determining HCWs motivation and satisfaction) in this case being listened to. However, the facilities struggled to employ specialists, and there were other HCWs staffing challenges.

Our study has highlighted the enhanced identification strategies for vulnerable populations (such as street children, orphans, and adolescents) that had initially been excluded from the policy on paper and are now using the policy. The findings align with the results of implementing the Safe Motherhood programme in Nigeria (Abiye initiative), which equally showed that removing user fees, particularly for the most vulnerable population, enhanced access and utility of service (81). However, in a different study in Kenya, researchers showed that the enhancement of the reach of the vulnerable population was mainly done by HCWs who, bound by ethics and professionalism, provided expanded FMP services to those excluded from the policy, such as foreigners, and those without IDs, such as street children who had no parents, refugees without IDs, or schoolgirls who were underage and pregnant. Hence, there is a need for official policy correction (82). While our results further show that there has been enhanced equity and financial access to the services by the women as those in the rural and urban areas received uniform services for free, in Benin, the CS policy exacerbated the inequalities as the policy reached the predominantly rich, exacerbating social exclusion (64).

Besides, from our findings, there is a positive perception of the policy despite the longer waiting times, particularly in the initial visits where mothers are accessing ANC additional benefit packages that were not in the previous policy. In contrast, a mixed-method study in Nigeria showed that mothers were dissatisfied with the waiting time under the free policy, but the authors did not link it to any particular service (83).

A rather interesting finding is the mothers' preference for higher-level facilities due to the perception of better services. Higher-level facilities are significantly burdened due to LM policy, leading to a ripple effect (where the facilities are left with a resource gap, as they use more resources to meet the mothers' specialised needs and manage deliveries that can be done at the periphery). However, it could also be argued that having more mothers in higher-level facilities means more claims and reimbursements. However, literature has attributed this preference to factors such as cleanliness, interpersonal skills, and other perceptions of better services;(84) and not the LM policy. A discrete choice experiment in Nigeria showed that the women chose to give birth in places with good condition of the health system, and absence of sexual, physical and verbal abuse, and that an unclean environment of birth without privacy and unclear user fees policy drove the women away (85). The mother's choice of higher-level facilities has led to QoC concerns such as indifference in the treatment based on the type of delivery and parity (partly because of overburdening higher facilities and the need for prioritisation). In Kenya, other studies have shown that mothers bypass lower-level facilities due to the perception of better quality (86, 87). Same case has been shown in Sri Lanka (88).

Interestingly, fewer mothers are being referred from lower to higher facilities than before the LM policy. While in the previous policy, complications were being referred to higher-level hospitals from lower-level health centres to seek better services (89), it could be argued that, through the LM policy,

For peer review only - http://bmjopen.bmj.com/site/about/guidelines.xhtml

lower-level facilities are making adequate investments using the LM policy reimbursements and are thus able to handle complications. That may nevertheless not be true as another finding in our study showed that the fewer referrals are mainly due to the lack of equipment, theatre, and NBU in the lower-level facilities. Thus, it could be that the policy confusion in the reimbursements of the services is somewhat hampering the positive quality effects of the policy. Other literature from Ghana concurs with this assumption. For instance, Witter et al.'s (90) exploration of the policy showed that the uncoordinated and unreimbursed referral strategy (particularly at referring hospitals) hampered the positive effect of the policy, while Ganle et al. (91) showed that Ghana's referral system was ineffective and the care was substandard because of a lack of critical care staff to handle healthcare emergencies.

The mothers who are referred have a positive perception of the referral process. This perception could be because the HCWs went above and beyond to provide referral elements, such as allowing the mothers to have companions at referral time and in the hospital. However, the lack of transport for referral could hamper the referral gains by either making the mother pay or risk their life looking for transport systems at the tail end of delivery. For example, Burkina Faso included transport in their subsidy policy to enhance mothers' referrals to health facilities (92). Through its well-organised rapid response to emergency and evacuation, mothers were positively satisfied with the referral system under the policy; however, IDIs with HCWs revealed no adequate follow-up to ensure the evacuated mothers received care as intended (93). Interestingly, Kenyan nurses under the LM policy went above and beyond to refer and follow up mothers, which was a compensatory mechanism for improving QoC.

In addition, through the LM policy, there has been some improved availability of equipment, supply, and infrastructure. The improvement could be due to the provider and facility in-charges using Streel Level Beureacrat tacts (such as renovations) to improve the facility and hence to attract more mothers who are the source of reimbursement funds (82). However, despite progress, some commodities, infrastructure, and supplies remain a challenge. The lack of supplies, equipment, and infrastructure contravenes the WHO statement number eight on quality, which shows that positive birth outcomes rely on their availability (41). A recent review showed that inadequacy is a global phenomenon compromising the quality of maternal care (94). Evidently, in all the facilities, the mothers revealed that they were satisfied with the characteristics of the facilities, such as having adequate rooms, adequate hand washing, bathing, and toilet facilities; in addition to equipment well suited for detecting women's problems. As is in this study, a mixed-methods study in Ghana showed that, despite the inadequate infrastructure in the facilities and lack of basic supplies, 89% of the mothers who participated in the EI, and those in the FGDs were satisfied with the quality of maternal care during childbirth (95) as is in this study. This postulates that mothers are more concerned about the interpersonal care received and the basic amenities provided if they can have live births and remain alive. The absence of or inadequacy of equipment and supplies compromises the QoC.

Equally interesting was that the good experience of care received by the women was based on the level of support provided by the HCWs and the facilities. Research shows that a good relationship between patients and HCWs could help improve trust, diffuse patients' anxieties, and create open communication (96). The majority of the mothers in both the FGDs and the EI attributed the good experience of care to the interpersonal skills exhibited by the HCWs, such as empathy, being friendly,

For peer review only - http://bmjopen.bmj.com/site/about/guidelines.xhtml

kindness, respect, devoting time, and honesty. The good care experiences the women receive influences their future delivery in the same facility. However, the findings could not show whether such experiences were due to LM policy, except that it incentivised the HCWs to provide FP and breastfeeding education. The finding shows that despite the challenges of the policy, the mothers appreciated and perceived the HCWs and health facility characteristics positively. This shows that HCWs have significantly contributed to the provision of care, but this may not lead to improved outcomes if the technical aspects of quality are not met. Similar findings have been reported elsewhere where, for instance, in Ghana, 77% of the mothers who participated in the EI noted that they were content with the HCWs service provision as they were patient and empathetic (95) or in Ethiopia, where 79.1% of the mothers interviewed were happy with the overall services provided (97).

The poor experience of care by the mothers hampers the technical QoC received. By sharing the beds due to overcrowding, the mothers are exposed to unhygienic practices that could eventually lead to nosocomial infection in the maternity facilities, which hampers QoC. A review of quality elements in facilities in the 14 counties in Kenya linked the introduction of LM services with poor hygiene and low privacy (29) Such findings are expected because investments in hospital infrastructure have not subsequently followed the increase in the number of mothers utilising maternal care. Other literature has shown similar findings in other settings with FM services (98-100).

The other finding of poor QoC experienced by the mothers, such as lack of attention, negligence and physical abuse, has been shown in other Kenyan literature. For instance, the beneficiaries of FM services in a study in Kakamega provincial hospital in Kenya noted that the HCWs negligence and use of vulgar language were demeaning to the patients (101). Food is an important component in the birth process and for mothers to report that the food they received during delivery is inadequate is as surprising as it is demeaning. Also, as is in this study, poor communication with the mothers or lack thereof may create an ethical dilemma, especially in contexts where patients do not consent to or are not explained for procedures (102). Mothers should play a role in the decisions of the care provided.

A key limitation of this study is that the EIs were conducted in one county, and it is plausible that there could be varied practices across other counties. The implication of this study is that it may be difficult to generalise the findings to all the other 47 counties in Kenya. Nonetheless, using IDIs and FGDs in this study provides an opportunity to unpack the issue at hand (quality of maternal care under LM policy) within its context and be analytically generalisable. The meta-issues identified by the study are likely to be found in other counties, even though they might manifest in different ways.

CONCLUSION

This study has demonstrated that LM policy has provided positive results of quality across all the broad quality domains: access to care (equitable and timely), provision of care (safe and effective), management and organisation, and the experience of care. There were positive elements such as minimised access barriers (cultural, financial, geographic), timeliness of care, and provider availability that have created functional referral systems and safety, and availability of essential physical resources and competent and motivated staff. The women in the study had a good care experience, which included reception of prompt maternal services, good care for the baby after birth, teaching about birth procedures, breastfeeding, and family planning. Further, the results have shown negative results from the policy hampering maternal care, such as the lack of supplies, equipment and

For peer review only - http://bmjopen.bmj.com/site/about/guidelines.xhtml

infrastructure, and referral challenges. Cross-cutting poor experiences from the women exist, such as overcrowding of the healthcare facilities, inadequate food supply, the lack of communication of treatment plans, and experiencing both physical and verbal abuse. There is a need to address the negative aspects of the study while strengthening the positives to achieve the SDG and UHC goals that seek to ensure reduced maternal morbidities and mortalities through access to quality service for every woman.

Figure 1: Combined frameworks used in this study for examining the quality of care across the continuum of maternal care (antenatal, perinatal, and postnatal care) under the free maternity policy in Kenya.

Figure 2: Overall satisfaction and future delivery

List of abbreviations: ANC: Antenatal care; **CS:** Caesarean Section; **FBO:** faith-based organisations; **FGD:** Focus group discussions; **FMP:** Free maternity policy; **HCWs:** healthcare workers; **IDI:** Indepth interviews; **LM:** Linda mama; **LMIC:** Low-and-middle-income countries; **LIC:** low-income countries; **MCH:** maternal and child health; **QoC:** quality of care; **SBA:** Skilled birth attendance; **SSA:** sub-Saharan Africa; **UHC:** Universal Health Coverage; **WHO:** World Health Organization

Twitter: @bonnyoyugi_snr

Acknowledgements: The authors acknowledge the data collection support of the EI data from research assistants: Rachel Murigu, Justus Miran, Billy Bortich, Valentine Olunga, Janet Moraa, Winnie Kaitany, and Shillar Jeptoo.

Contributors: BO: conceptualised the study, curated and analysed the data, and drafted the initial manuscript which was subsequently revised for important intellectual content by all authors. ZAP: reviewed and edited all the drafts. SK and SP: contributed to the design and supervised the study. All authors read and approved the final manuscript.

Funding: This study was funded by the Commonwealth Scholarship Commission (KECS-2017-266), which supported BO's PhD study. The funding agency did not play any role in the analysis, interpretation of results, and manuscript writing.

Competing interests: None declared.

Patient consent for publication: Consent obtained directly from patient(s).

Patient and public involvement: Patients or the public were not involved in the design, or conduct, or reporting, or dissemination plans of our research.

Ethics approval: Ethical approval was obtained from the University of Kent, SSPSSR Students Ethics Committee and AMREF Scientific and Ethics Review Unit in Kenya (Ref: AMREF – ESRC P537/2018). Further written permission to conduct the study was received from the county government of Kiambu, and all the participating hospitals. Written and oral informed consent was obtained from the potential participants before starting the interviews.

For peer review only

Data availability statement: The datasets used in this study are available from BO on b.oyugi@kent.ac.uk upon reasonable request.

Supplementary material: The authors have supplied this content.

For peer review only

REFERENCES

1. World Health Organization. Trends in maternal mortality 2000 to 2020: estimates by WHO, UNICEF, UNFPA, World Bank Group and UNDESA/Population Division Geveva: World Health Organization; 2023 [Available from: https://www.who.int/publications/i/item/9789240068759.
2. Black RE, Walker N, Laxminarayan R, Temmerman M. Reproductive, Maternal, Newborn, and Child Health: Disease Control Priorities Washington (DC): The International Bank for Reconstruction and Development / The World Bank; 2016 [Third Edition (Volume 2):[1]. Available from: https://www.ncbi.nlm.nih.gov/books/NBK361907/.
3. Kyei-Nimakoh M, Carolan-Olah M, McCann TV. Access barriers to obstetric care at health facilities in sub-Saharan Africa—a systematic review. Systematic reviews. 2017;6:1-16.
4. Gabrysch S, Campbell OM. Still too far to walk: literature review of the determinants of delivery service use. BMC pregnancy and childbirth. 2009;9(1):34.
5. Kenya National Bureau of Statistics, ICF. Kenya Demographic and Health Survey 2022. Key Indicators Report Nairobi, Kenya, and Rockville, Maryland, USA: KNBS and ICF; 2023 [Available from: https://dhsprogram.com/publications/publication-PR143-Preliminary-Reports-Key-Indicators-Reports.cfm.
6. UNICEF. Neonatal mortality: UNICEF; 2020 [Available from: https://data.unicef.org/topic/child-survival/neonatal-mortality/.
7. Kenya Ministry of Health. Reducing Maternal and Neonatal Mortality in Kenya: Scaling up Effective Interventions in Maternal and Newborn Health, An Implementation Plan for the period 2016 - 2018. In: Reproductive and Maternal Health Services Unit, Neonatal Child and Adolescent Health, Community Health Services Unit, Maternal and Newborn Health Technical Working Group, editors. Nairobi: Kenya Ministry of Health; 2016.
8. World Health Organisation. Trends in Maternal Mortality 2000 to 2017: Estimates by WHO, UNICEF, UNFPA, World Bank Group and the United Nations Population Division. Geneva: World Health Organization; 2019.
9. Chuma J, Maina T. Free Maternal Care and Removal of User Fees at Primary-Level Facilities in Kenya. Washington, DC: Health Policy Project, Futures Group; 2013.
10. Janisch C, Albrecht M, Wolfschuetz A, Kundu F, Klein S. Vouchers for health: a demand side output-based aid approach to reproductive health services in Kenya. Global Public Health. 2010;5(6):578-94.
11. Chuma J, Musimbi J, Okungu V, Goodman C, Molyneux C. Reducing user fees for primary health care in Kenya: policy on paper or policy in practice? International Journal for Equity in Health. 2009;8:15.
12. Chuma J, Okungu V. Viewing the Kenyan health system through an equity lens: implications for universal coverage. International Journal for Equity in Health. 2011;10(1):22.
13. Opwora A, Kabare M, Molyneux S, Goodman C. Direct facility funding as a response to user fee reduction: implementation and perceived impact among Kenyan health centres and dispensaries. Health Policy and Planning. 2010;25(5):406-18.
14. Tama E, Molyneux S, Waweru E, Tsofa B, Chuma J, Barasa E. Examining the implementation of the free maternity services policy in Kenya: a mixed methods process evaluation. International Journal of Health Policy and Management. 2018;7(7):603-13.
15. Orangi S, Kairu A, Ondera J, Mbuthia B, Koduah A, Oyugi B, et al. Examining the implementation of the Linda Mama free maternity program in Kenya. The International Journal of Health Planning and Management. 2021.
16. Oyugi B, Audi-Poquillon Z, Kendall S, Peckham S, Barasa E. The policy formulation process, and the role of actors in the policy formulation and implementation process: A policy analysis of the Kenyan free maternity policy. medRxiv. 2024:2024.01.26.23300268.
17. Ombere OS, Nyambedha OE, Haller T, Merten S. Local perspectives on policy implementation of free maternity health services in Kenya: Implications for universal health coverage. 2023.

For peer review only - http://bmjopen.bmj.com/site/about/guidelines.xhtml

18. National health Insurance Fund. Linda Mama Services Nairobi: National health Insurance Fund; 2017 [Available from: http://www.nhif.or.ke/healthinsurance/lindamamaServices.
19. Kenya Ministry of Health, National Hospital Insurance Fund. Implementation Manual for Programme Managers Nairobi: National Hospital Insurance Fund; 2016 [Available from: https://www.health.go.ke/wp-content/uploads/2018/11/implementation-manual-softy-copy-sample.pdf.
20. The Executive Office of the President. The Big 4 Agenda Nairobi: President's Delivery Unit; 2017 [Available from: https://big4.delivery.go.ke/.
21. World Health Organisation. The World Health Report 2010 Health Systems Financing: The path to universal coverage. Geneva: World Health Organization; 2010.
22. World Health Organisation. Arguing for Universal Health coverage. 2013.
23. Orangi S, Kairu A, Malla L, Ondera J, Mbuthia B, Ravishankar N, et al. Impact of free maternity policies in Kenya: an interrupted time-series analysis. BMJ Global Health. 2021;6(6):e003649.
24. Gitobu C, Gichangi P, Mwanda W. Patterns in maternal mortality following the implementation of a free maternal health care policy in Kenyan public health facilities. East African Medical Journal. 2017;94(6):433-44.
25. Gitobu C, Gichangi P, Mwanda W. Causes of neonatal mortality two years before and after the implementation of a free maternal health care policy in Kenyan public health facilities. East African Medical Journal. 2017;94(5):323-35.
26. Lang'at E, Mwanri L, Temmerman M. Effects of implementing free maternity service policy in Kenya: an interrupted time series analysis. BMC Health Service Research. 2019;19(1):645.
27. Oyugi B, Nizalova O, Kendall S, Peckham S. Does a free maternity policy in Kenya work? Impact and cost-benefit consideration based on demographic health survey data. Eur J Health Econ. 2023.
28. Oyugi B, Kendall S, Peckham S, Barasa E. Out of pocket payments during childbirth in Kenya under the free maternity services: Perspectives of mothers, healthcare workers and county officials. Wellcome Open Research. 2023.
29. Gitobu CM, Gichangi PB, Mwanda WO. Satisfaction with delivery services offered under the free maternal healthcare policy in Kenyan public health facilities. Journal of Environmental and Public Health. 2018;2018:4902864.
30. Owiti A, Oyugi J, Essink D. Utilization of Kenya's free maternal health services among women living in Kibera slums: a cross-sectional study. Pan Afr Med J. 2018;30:86.
31. Lusambili AM, Naanyu V, Wade TJ, Mossman L, Mantel M, Pell R, et al. Deliver on Your Own: Disrespectful Maternity Care in rural Kenya. PLOS ONE. 2020;15(1):e0214836.
32. Donabedian A. The quality of care: how can it be assessed? The Journal of the American Medical Association. 1988;260(12):1743-8.
33. Donabedian A. The seven pillars of quality. Archives of Pathology and Laboratory Medicine. 1990;114(11):1115-8.
34. Owili PO, Muga MA, Mendez BR, Chen B. Quality of maternity care and its determinants along the continuum in Kenya: A structural equation modeling analysis. PLoS One. 2017;12(5):e0177756.
35. Kruk ME, Gage AD, Arsenault C, Jordan K, Leslie HH, Roder-DeWan S, et al. High-quality health systems in the Sustainable Development Goals era: time for a revolution. The Lancet Global Health. 2018;6(11):e1196-e252.
36. World Health Organization. The network for improving quality of care for maternal, newborn and child health: evolution, implementation and progress: 2017-2020 report. 2021.
37. Creswell JW, Clark VLP. Designing and Conducting Mixed Methods Research. Third edition ed. Los Angeles: Sage publications, Inc; 2017.
38. Kelley E, Hurst J. Health Care Quality Indicators Project: Conceptual Framework Paper: Organisation for Economic Co-operation and Development 2006 [cited 2018 13th May]. Available from:
http://thuvien.thanglong.edu.vn:8081/dspace/bitstream/DHTL_123456789/3992/1/No.%2023%20H

For peer review only

For peer review only - http://bmjopen.bmj.com/site/about/guidelines.xhtml

ealthcare%20quality%20indicators%20project%20%E2%80%93%20Conceptual%20framework%20paper.pdf.
39. Akachi Y, Kruk ME. Quality of care: measuring a neglected driver of improved health. Bulletin of the World Health Organization. 2017;95(6):465.
40. World Health Organization. Quality of Care for Maternal and Newborn Health: A Monitoring Framework for Network Countries Avenue Appia 20, 1211 Genève, Switzerland: Department of Maternal, Newborn, Child and Adolescent Health (MCA), World Health Organization HQ; 2019 [Available from: https://www.who.int/publications/m/item/quality-of-care-for-maternal-and-newborn--a-monitoring-framework-for-network-countries.
41. World Health Organisation. Standards for Improving Quality of Maternal and Newborn Care in Health Facilities 20 Avenue Appia, 1211 Geneva 27, Switzerland: World Health Organisation; 2016 [Available from: https://apps.who.int/iris/bitstream/handle/10665/249155/9789241511216-per.pdf.
42. Kenya Ministry of Health. Kenya Community Health Policy 2020-2030. In: Division of Community Health, editor. Nairobi, Kenya: Kenya Ministry of Health; 2020.
43. Kenya National Bureau of Statistics. 2019 Kenya Population and Housing Census Volume I: Population by County and Sub-County Nairobi: Kenya National Bureau of Statistics; 2019 [cited 2020 27 March]. Available from: https://www.knbs.or.ke/?wpdmpro=2019-kenya-population-and-housing-census-volume-i-population-by-county-and-sub-county.
44. County Government of Kiambu. About Kiambu County 2018 [cited 2020 27 March]. Available from: https://kiambu.go.ke/about-us/#2.
45. Kenya National Bureau of Statistics, Kenya Ministry of Health, National AIDS Control Council, Kenya Medical Research institute, National Council for Population and Development, ICF international. Kenya Demographic and Health Survey 2014 Calverton, MD: Kenya National Bureau of Statistics & ICF international; 2014 [cited 2020 5th May]. Available from: https://dhsprogram.com/pubs/pdf/fr308/fr308.pdf.
46. Kruk ME, Leslie HH, Verguet S, Mbaruku GM, Adanu RMK, Langer A. Quality of basic maternal care functions in health facilities of five African countries: an analysis of national health system surveys. The Lancet Global Health. 2016;4(11):e845-e55.
47. Kiambu County Government. Health services Kiambu: Midas Africa; 2024 [Available from: https://kiambu.go.ke/departments/health-services/.
48. Kahenda M. They live in Nairobi, but give birth in Kiambu Nairobi: The Standard; 2023 [Available from: https://www.standardmedia.co.ke/health/health-science/article/2001442841/they-live-in-nairobi-but-give-birth-in-kiambu.
49. Kamau KJ, Osuga BO, Njuguna S. Challenges facing implementation of referral system for quality health care services in Kiambu county, Kenya. 2017.
50. Okoroafor SC, Kwesiga B, Ogato J, Gura Z, Gondi J, Jumba N, et al. Investing in the health workforce in Kenya: trends in size, composition and distribution from a descriptive health labour market analysis. BMJ Global Health. 2022;7(Suppl 1):e009748.
51. Perfomance Monitoring for Action. Kenya (Kiambu): Results from phase 2 cross-sectional survey - (section 1: contraceptive use, dynamic, and demand) 2020 [Available from: https://www.pmadata.org/sites/default/files/data_product_results/Kenya%20Kiambu_Phase%202%20XS%20Results_Brief_final.pdf.
52. Muchemi OM, Gichogo AW, Mungai JG, Roka ZG. Trends in health facility based maternal mortality in Central Region, Kenya: 2008-2012. Pan African Medical Journal. 2016;23(1).
53. Hennink MM, Kaiser BN, Marconi VC. Code saturation versus meaning saturation: how many interviews are enough? Qualitative Health Research. 2017;27(4):591-608.
54. Gorstein J, Sullivan K, Parvanta I, Begin F. Indicators and Methods for Cross-Sectional Surveys of Vitamin and Mineral Status of Populations: The Micronutrient Initiative (Ottawa) and the Centers for Disease Control and Prevention (Atlanta); 2007 [Available from: https://www.who.int/vmnis/toolkit/mcn-micronutrient-surveys.pdf.

For peer review only

55. Oyugi B. The Policy Process, Quality and Cost of Free Maternal Healthcare in Kenya: A Mixed Methods Analysis of Maternity Policy: University of Kent, Centre for Health Services Studies, University of Kent; 2021.
56. Dalinjong PA, Wang AY, Homer CSE. The operations of the free maternal care policy and out-of-pocket payments during childbirth in rural Northern Ghana. Health Economics Review. 2017;7(1):41.
57. StatTrek.com. Random Number Generator: Stat Trek; 2021 [cited 2019 20 October]. Available from: https://stattrek.com/statistics/random-number-generator.aspx.
58. Kenya Ministry of Health. Kenya Master Health Facility List (KMHFL): Kenya Ministry of Health,; 2020 [Available from: http://kmhfl.health.go.ke/#/home.
59. DHIS2. Kenya Health Information System (KHIS) for Aggregate Reporting: DHIS2; 2020 [cited 2020 16 March]. Available from: https://hiskenya.org/dhis-web-commons/security/login.action.
60. Ritchie J, Spencer L. Qualitative data analysis for applied policy research. In: Bryman A, Burgess RG, editors. Analysing Qualitative Data. New York, NY: Routledge, Taylor & Francis Group; 2002. p. 173-94.
61. Yin KR. Case Study Research and Applications: Design and Methods. 6th ed. Thousand Oaks, CA: SAGE Publications; 2018.
62. Dzakpasu S, Powell-Jackson T, Campbell OM. Impact of user fees on maternal health service utilization and related health outcomes: a systematic review. Health Policy and Planning. 2014;29(2):137-50.
63. Oyugi B, Kendall S, Peckham S. Effects of free maternal policies on quality and cost of care and outcomes: an integrative review. Primary health care research & development. 2021;22.
64. Dossou J-P, Cresswell JA, Makoutodé P, De Brouwere V, Witter S, Filippi V, et al. 'Rowing against the current': the policy process and effects of removing user fees for caesarean sections in Benin. BMJ Global Health. 2018;3(1):e000537.
65. Okumu C, Oyugi B. Clients' satisfaction with quality of childbirth services: a comparative study between public and private facilities in Limuru Sub-County, Kiambu, Kenya. PLoS One. 2018;13(3):e0193593.
66. Chirdan O, Lar L, Afolaranmi T, Inalegwu E, Igoh C, Adah G. Client satisfaction with maternal health services comparism between public and private hospitals in Jos Nigeria. Jos Journal of Medicine. 2013;7(1):1-9.
67. Khan REA, Noreen S. Household choice of public versus private health institution for maternal health-care: a case study of Bahawalpur (Pakistan). Pakistan Journal of Commerce and Social Sciences. 2016;10(3):444-60.
68. Oluoch-Aridi J, Wafula F, Kokwaro G, Adam MB. 'We just look at the well-being of the baby and not the money required': a qualitative study exploring experiences of quality of maternity care among women in Nairobi's informal settlements in Kenya. BMJ Open. 2020;10(9):e036966.
69. Amooti-Kaguna B, Nuwaha F. Factors influencing choice of delivery sites in Rakai district of Uganda. Social Science & Medicine. 2000;50(2):203-13.
70. Parkhurst JO, Penn-Kekana L, Blaauw D, Balabanova D, Danishevski K, Rahman SA, et al. Health systems factors influencing maternal health services: a four-country comparison. Health Policy. 2005;73(2):127-38.
71. Escamilla V, Calhoun L, Winston J, Speizer IS. The role of distance and quality on facility selection for maternal and child health services in urban Kenya. Journal of Urban Health. 2018;95(1):1-12.
72. Fleming LC, Ansumana R, Bockarie A, Alejandre J, Bangura U, Jimmy DH, et al. Inpatient healthcare provider bypassing by women and their children in urban Bo, Sierra Leone. The Pan African medical journal. 2016;23:146.
73. Pyone T, Smith H, van dB. Implementation of the free maternity services policy and its implications for health system governance in Kenya. BMJ Global Health. 2017;2(4):e000249.

For peer review only - http://bmjopen.bmj.com/site/about/guidelines.xhtml

74. Lang'at E, Mwanri L. Healthcare service providers' and facility administrators' perspectives of the free maternal healthcare services policy in Malindi District, Kenya: a qualitative study. Reproductive Health. 2015;12:59.
75. Njuguna J, Kamau N, Muruka C. Impact of free delivery policy on utilization of maternal health services in county referral hospitals in Kenya. BMC Health Services Research. 2017;17(1):429.
76. Miseda MH, Were SO, Murianki CA, Mutuku MP, Mutwiwa SN. The implication of the shortage of health workforce specialist on universal health coverage in Kenya. Human Resources for Health. 2017;15(1):80.
77. Tawfik DS, Scheid A, Profit J, Shanafelt T, Trockel M, Adair KC, et al. Evidence relating health care provider burnout and quality of care: a systematic review and meta-analysis. Annals of Internal Medicine. 2019;171(8):555-67.
78. Salyers MP, Bonfils KA, Luther L, Firmin RL, White DA, Adams EL, et al. The relationship between professional burnout and quality and safety in healthcare: a meta-analysis. Journal of General Internal Medicine. 2017;32(4):475-82.
79. Franco LM, Bennett S, Kanfer R. Health sector reform and public sector health worker motivation: a conceptual framework. Social Science & Medicine. 2002;54(8):1255-66.
80. Herzberg F, Mausner B, Snyderman B. The Motivation to Work. 2 ed. New York: John Wiley & Sons; 1959.
81. Ajayi AI, Akpan W. Maternal health care services utilisation in the context of 'Abiye' (safe motherhood) programme in Ondo State, Nigeria. BMC Public Health. 2020;20(1):362.
82. Oyugi B, Kendall S, Peckham S, Orangi S, Barasa E. Exploring the Adaptations of the Free Maternity Policy Implementation by Health Workers and County Officials in Kenya. Global Health: Science and Practice. 2023;11(5).
83. Ajayi AI. "I am alive; my baby is alive": understanding reasons for satisfaction and dissatisfaction with maternal health care services in the context of user fee removal policy in Nigeria. PLoS One. 2019;14(12).
84. Oyugi B, Kioko U, Kaboro SM, Okumu C, Ogola-Munene S, Kalsi S, et al. A facility-based study of women'satisfaction and perceived quality of reproductive and maternal health services in the Kenya output-based approach voucher program. BMC pregnancy and childbirth. 2018;18(1):310.
85. Umar N, Quaife M, Exley J, Shuaibu A, Hill Z, Marchant T. Toward improving respectful maternity care: a discrete choice experiment with rural women in northeast Nigeria. BMJ Glob Health. 2020;5(3):e002135.
86. Audo M, Ferguson A, Njoroge P. Quality of health care and its effects in the utilisation of maternal and child health services in Kenya. East African Medical Journal. 2005;82(11):547.
87. Cohen J, Golub G, Kruk ME, McConnell M. Do active patients seek higher quality prenatal care?: a panel data analysis from Nairobi, Kenya. Preventive Medicine. 2016;92:74-81.
88. Perera S, Weerasinghe M. Bypassing primary care in Sri Lanka: a comparative study on reasons and satisfaction. Vietnam Journal of Public Health. 2015;3(1):69-76.
89. Sidze EM, Fenenga C, Amendah DD, Maina TM, Mutua MK, Mulupi SK, et al. Are free maternal healthcare services programs an impediment to quality care? an examination of the Kenyan experience. African Population and Health Research Center. 2015;5(1):1-10.
90. Witter S, Arhinful DK, Kusi A, Zakariah-Akoto S. The experience of Ghana in implementing a user fee exemption policy to provide free delivery care. Reproductive Health Matters. 2007;15(30):61-71.
91. Ganle JK, Parker M, Fitzpatrick R, Otupiri E. A qualitative study of health system barriers to accessibility and utilization of maternal and newborn healthcare services in Ghana after user-fee abolition. BMC pregnancy and childbirth. 2014;14(1):434-62.
92. Ridde V, Queuille L, Kafando Y, Robert E. Transversal analysis of public policies on user fees exemptions in six West African countries. BMC Health Services Research. 2012;12:409.
93. Ridde V, Diarra A. A process evaluation of user fees abolition for pregnant women and children under five years in two districts in Niger (West Africa). BMC Health Services Research. 2009;9:1-16.

For peer review only - http://bmjopen.bmj.com/site/about/guidelines.xhtml

94. Bohren MA, Vogel JP, Hunter EC, Lutsiv O, Makh SK, Souza JP, et al. The mistreatment of women during childbirth in health facilities globally: a mixed-methods systematic review. PLoS Medicine. 2015;12(6):e1001847.
95. Dalinjong PA, Wang AY, Homer CS. Are health facilities well equipped to provide basic quality childbirth services under the free maternal health policy? findings from rural Northern Ghana. BMC Health Services Research. 2018;18(1):959.
96. Okoror CEM, Enabudoso EJ, Okoroh MI. Women's perception and satisfaction with the quality of antenatal care services in mission hospitals in Benin City, Nigeria. Pyramid Journal of Medicine. 2020;3(1).
97. Tesfaye R, Worku A, Godana W, Lindtjorn B. Client satisfaction with delivery care service and associated factors in the public health facilities of Gamo Gofa zone, Southwest Ethiopia: in a resource limited setting. Obstetrics and Gynecology International. 2016;2016:1-7.
98. Masaba BB, Mmusi-Phetoe RM. Free maternal health care policy in Kenya: level of utilization and barriers. International Journal of Africa Nursing Sciences. 2020;13:100234.
99. Chesumei EJ. Factors associated with uptake of free maternity services at Baringo County Referral Hospital: Jomo Kenyatta University of Agriculture and Technology; 2019.
100. Witter S, Adjei S, Armar-Klemesu M, Graham W. Providing free maternal health care: ten lessons from an evaluation of the national delivery exemption policy in Ghana. Global Health Action. 2009;2(1):1881.
101. Asule BM, Kwena A, Wambui T. Effects of the free maternity care program on utilization of services at a county referral hospital in Kenya. Kenyan Journal of Nursing & Midwifery. 2017;1(2).
102. Khumalo N, Rwakaikara E. Patient satisfaction with peri-partum care at Bertha Gxowa district hospital, South Africa. African Journal of Primary Health Care & Family Medicine. 2020;12(1):a2409.

For peer review only

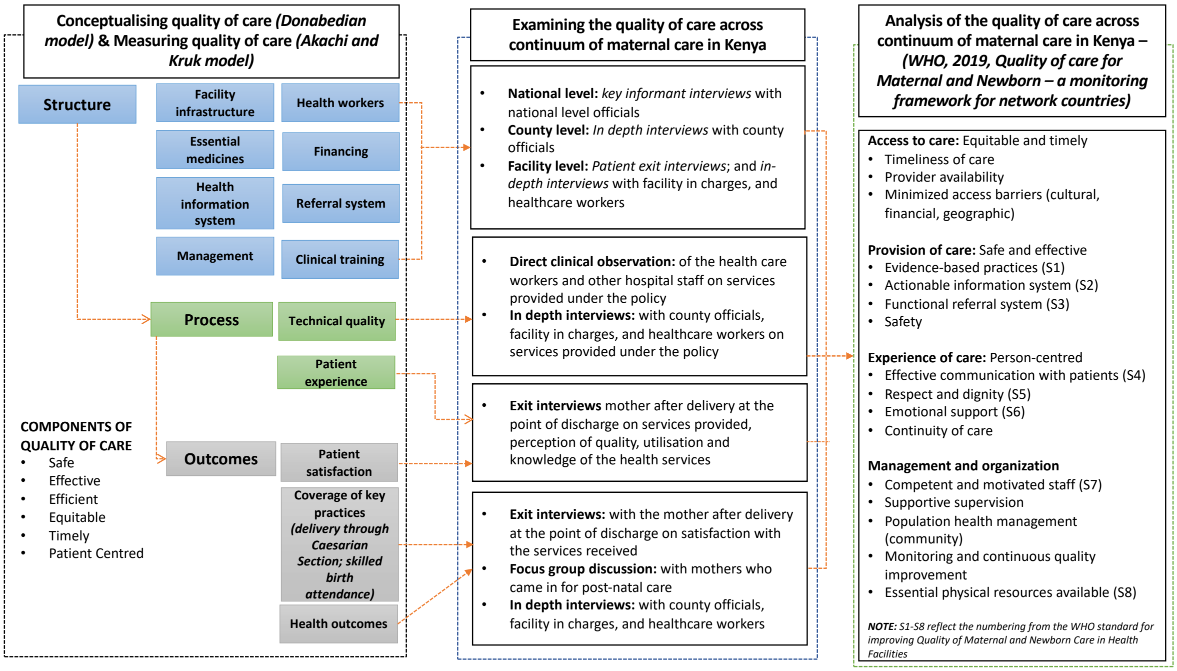

For peer review only - http://bmjopen.bmj.com/site/about/guidelines.xhtml

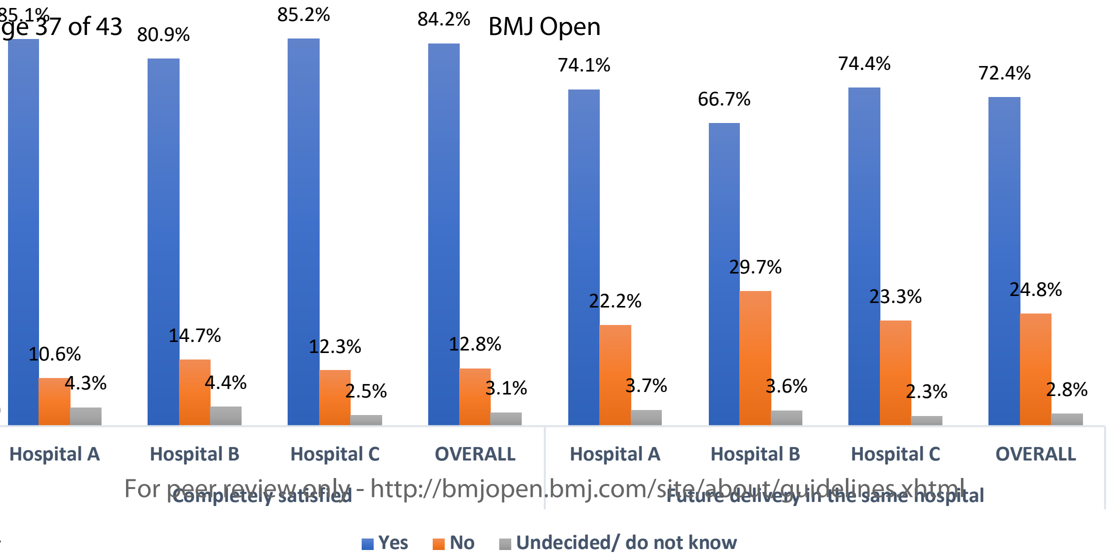

For peer review only - http://bmjopen.bmj.com/site/about/guidelines.xhtml

Appendix 1: Maternal healthcare access characteristics

**Variable**		**Total Frequency (%) n=550**	**Hospital A n (%) n=42**	**Hospital B n (%) n=170**	**Hospital C n (%) n=338**	**p-value**
Facility visited during pregnancy	Yes	545 (99.09)	42 (100)	169 (99.41)	334 (98.82)	0.650
	No	5 (0.91)	0	1 (0.59)	4 (1.18)	
Type of facility visited	Public facility	506 (92.00)	38 (90.48)	149 (87.65)	319 (94.38)	**P<0.001***
	Private facility	28 (5.09)	1 (2.38)	17 (10.00)	10 (2.96)	
	Faith based organization (Mission)	7 (1.27)	-	3 (1.76)	4 (1.18)	
	Other	9 (1.64)	3 (7.14)	1 (0.59)	5 (1.48)	
Time taken to reach hospital	Below 30 minutes	137 (24.91)	12 (28.57)	45 (26.47)	80 (23.67)	0.309
	30 minutes-1 hour	264 (48.00)	19 (45.24)	87 (51.18)	158 (46.75)	
	1 hour-2 hours	121 (22.00)	7 (16.67)	35 (20.59)	79 (23.37)	
	More than 2 hours	20 (3.64)	4 (9.52)	2 (1.18)	14 (4.14)	
	Don't know	8 (1.45)	-	1(0.59)	7 (1.96)	
Perception of the time take to reach the hospital	Very short	60 (10.91)	6 (14.29)	25 (14.71)	29 (8.58)	0.340
	Short	249 (45.27)	18 (42.86)	73 (42.94)	158 (46.75)	
	Normal	99 (18.00)	12 (28.57)	32 (18.82)	55 (16.27)	
	Long	107 (19.45)	5 (11.90)	29 (17.06)	73 (21.60)	
	Very long	32 (5.82)	1 (2.38)	11 (6.47)	20 (5.92)	
	Don't know	3 (0.54)	-	-	3 (0.89)	
	Very near	69 (12.55)	16 (38.10)	22 (12.94)	31 (9.17)	**P<0.001**

For peer review only

Variable		Total Frequency (%) n=550	Hospital A n (%) n=42	Hospital B n (%) n=170	Hospital C n (%) n=338	p-value
Perception about distance to the facility	Normal	339 (61.64)	17 (40.48)	107 (62.94)	215 (63.61)	
	Far	110 (20.00)	8 (19.05)	33 (19.41)	69 (20.41)	
	Very far	28 (5.09)	-	8 (4.71)	20 (5.92)	
	Don't know	4 (0.73)	1 (2.38)	-	3 (0.89)	
Means of transport to the facility	Walking	27 (4.91)	7 (16.67)	14 (8.24)	6 (1.78)	**P<0.001**
	Bi/Motorcycle	60 (10.91)	1 (2.38)	20 (11.76)	39 (11.54)	
	Public transport (matatu/tuk tuk)	224 (40.73)	8 (19.05)	55 (32.35)	161 (47.63)	
	Private car/taxi	211 (38.36)	24 (57.14)	78 (45.88)	109 (32.25)	
	Ambulance	22 (4.00)	-	1 (0.59)	21 (6.21)	
	Combined modes	6 (1.09)	2 (4.76)	2 (1.18)	2 (0.59)	
Does opening hour suit your time?	Yes	431 (78.36)	41 (97.62)	152 (89.41)	238 (70.41)	**P<0.001**
	No	9 (1.64)	1 (2.38)	1 (0.59)	7 (2.07)	
	Don't know	76 (13.82)	-	15 (8.82)	61 (18.05)	
	N/A	34 (6.18)	-	2 (1.18)	32 (9.47)	
Waiting time at the facility	Very short	80 (14.55)	12 (28.57)	26 (15.29)	42 (12.43)	**P<0.001**
	Short	237 (43.09)	16 (38.10)	72 (42.35)	149 (44.08)	
	Normal	70 (12.73)	11 (26.19)	28 (16.47)	31 (9.17)	
	Long	80 (14.55)	1 (2.38)	22 (12.94)	57 (16.86)	
	Very long	43 (7.82)	2 (4.76)	22 (12.94)	19 (5.62)	
	N/A	40 (7.27)	-	-	40 (11.83)	

For peer review only

Variable		Total Frequency (%) n=550	Hospital A n (%) n=42	Hospital B n (%) n=170	Hospital C n (%) n=338	p-value
Hospital have a proper waiting area	Yes	422 (76.73)	40 (95.24)	134 (78.82)	248 (73.37)	**0.005**
	No	85 (15.45)	1 (2.38)	29 (17.06)	55 (16.27)	
	Don't know	28 (5.09)	1 (2.38)	7 (4.12)	20 (5.92)	
	N/A	15(2.73)	-	-	15 (4.44)	

Note: Chi square test of proportion was used to test difference in overall proportions of maternal health access characteristics.
*There is a statistical difference in the type of facilities that the mothers visited (majority visited public facilities).
Bold means **p-value <0.05**

For peer review only

Appendix 2: Referral characteristics

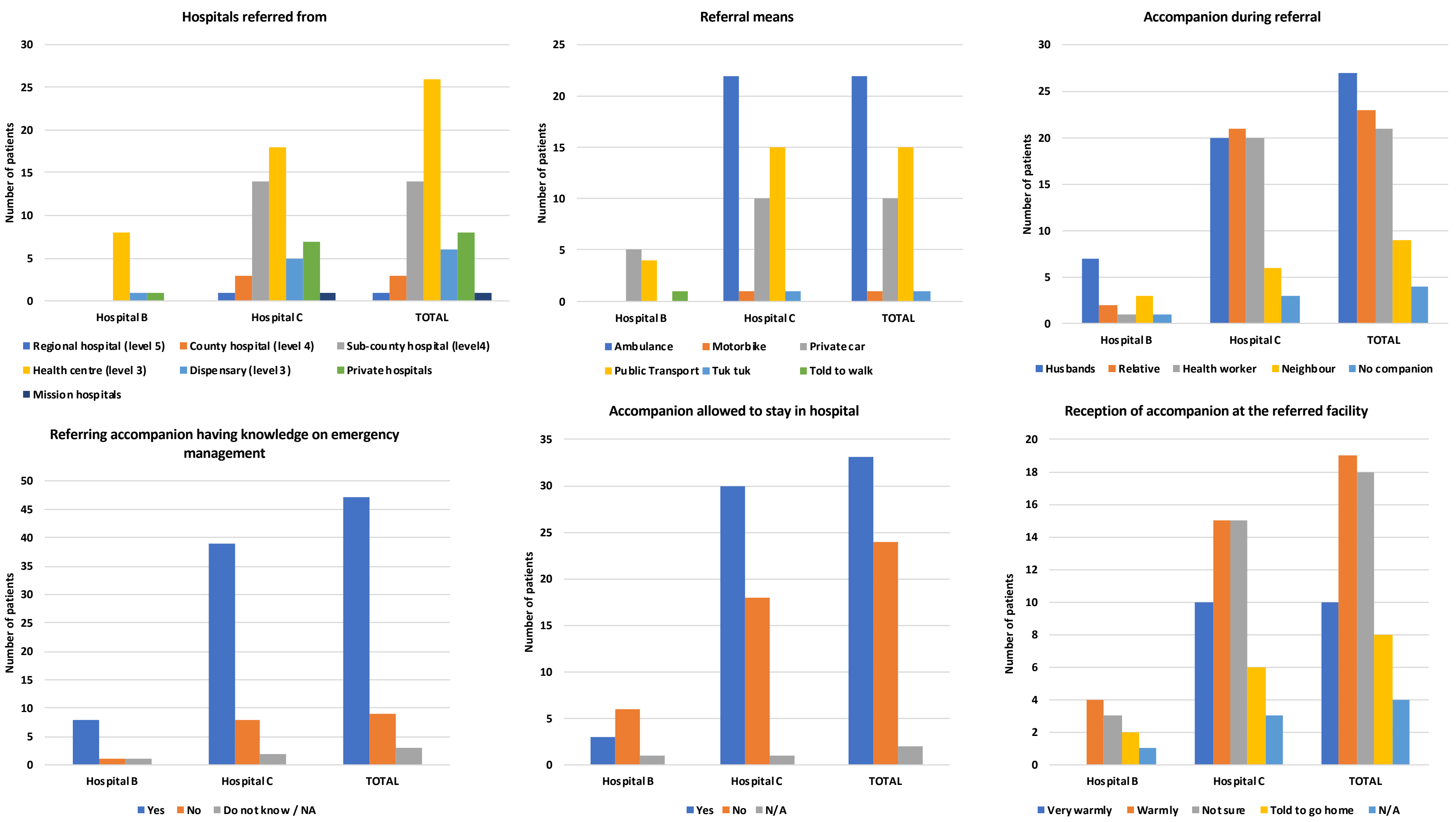

Appendix 3: Reasons for referral

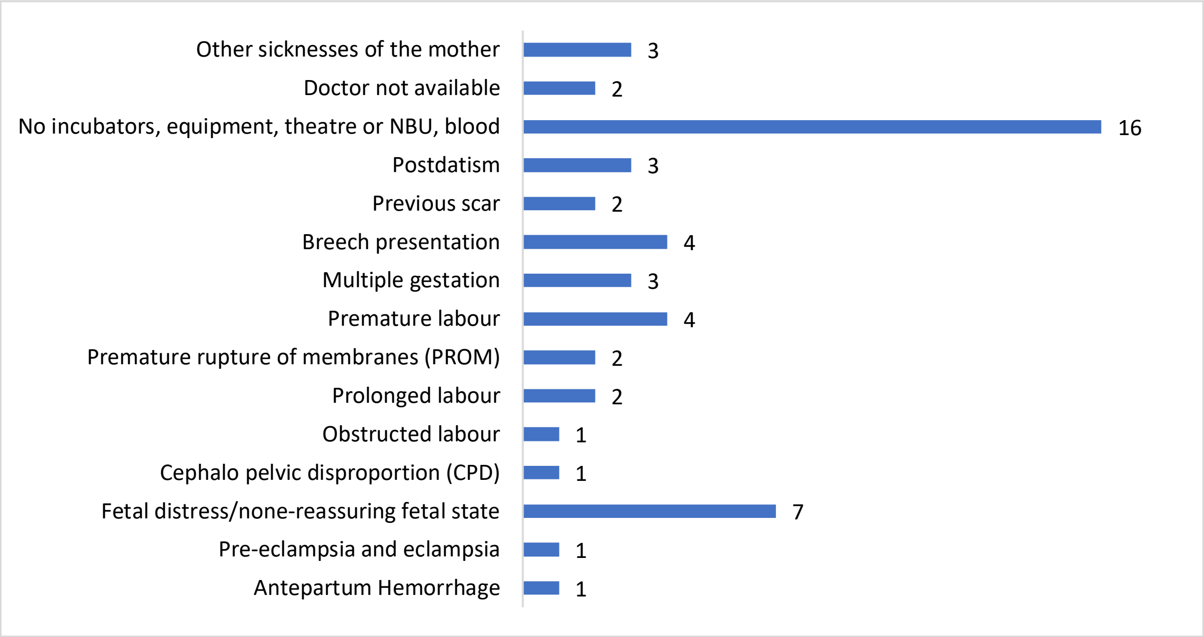

Appendix 4: Perception of quality of maternal care from the mothers exit interviews

Health Facility	Completely Disagree	Disagree	Not Sure	Agree	Completely Agree
Number of staff adequate	0.73%	19.09%	3.82%	57.82%	18.55%
Staff well suited to treat women	0.36%	1.82%	1.27%	70.73%	25.82%
Waiting and examination rooms adequate	5.82%	38.73%	3.82%	40.55%	11.05%
Provision of clean drinking water adequate	3.82%	38.36%	11.46%	34.00%	12.18%
Hand washing facilities adequate	1.45%	6.00%	0.73%	70.55%	21.27%
Bathing facilities adequate	3.82%	24.36%	4.36%	52.91%	14.55%
Toilet facilities adequate	2.91%	24.00%	1.64%	55.45%	16.00%
Overal facility environment very clean	0.91%	3.64%	2.00%	70.00%	23.45%
Well suited equipment for detecting women's problems	0.91%	3.45%	4.73%	69.82%	21.09%
Distance from home very far	8.55%	59.09%	2.18%	23.82%	6.36%
**Healthcare delivery**	**Completely Disagree**	**Disagree**	**Not Sure**	**Agree**	**Completely Agree**
Staff examine pregnant and post partum women well	0.91%	2.00%	1.82%	72.00%	23.27%
Staff very capable of finding out what is wrong with patients	0.73%	1.64%	2.18%	71.09%	24.36%
Staff prescribe drugs that are needed	0.00%	2.91%	37.45%	42.91%	16.73%
Drugs supplied by health facility are good	0.36%	1.45%	39.82%	42.55%	15.82%
Patients can obtain drugs from health facility easily	1.45%	5.64%	25.64%	52.00%	15.27%
Facility provided privacy very much during VE and delivery	3.82%	9.64%	6.73%	63.82%	16.00%
Felt very much of necessary procedure during ANC and delivery	3.83%	8.38%	3.10%	65.39%	19.31%
Adequate Information on danger signs of delivery and postpartum	1.45%	0.24%	3.27%	49.45%	21.82%
**Interpersonal Aspects**	**Completely Disagree**	**Disagree**	**Not Sure**	**Agree**	**Completely Agree**
Staff very open with the patients	0.18%	3.83%	1.64%	68.61%	25.73%
Staff very compasionate towards the patients	1.27%	5.45%	2.73%	66.00%	24.58%

Staff are respectful towards the patients	0.18%	2.73%	1.64%	69.64%	25.82%
Time staff devete to the patients is adequate	0.36%	4.36%	1.09%	67.27%	26.91%
Staff are very honest	0.00%	2.36%	5.64%	65.64%	26.36%

For peer review only
